# Cooperation of MLL1 and Jun in controlling H3K4me3 on enhancers in colorectal cancer

**DOI:** 10.1186/s13059-023-03108-3

**Published:** 2023-11-27

**Authors:** Xiang Lin, Ji-Dong Chen, Chen-Yu Wang, Zhen Cai, Rui Zhan, Chen Yang, La-Ying Zhang, Lian-Yun Li, Yong Xiao, Ming-Kai Chen, Min Wu

**Affiliations:** 1 Frontier Science Center for Immunology and Metabolism, Hubei Key Laboratory of Cell Homeostasis, Hubei Key Laboratory of Developmentally Originated Disease, College of Life Sciences, Taikang Center for Life and Medical Sciences, Renmin Hospital of Wuhan University, Wuhan University, Wuhan, Hubei 430072 China; 2 Department of Gastroenterology, Renmin Hospital of Wuhan University, Wuhan University, Wuhan, Hubei 430072 China

**Keywords:** Enhancers, H3K4me3, MLL1, JUN, Colorectal cancer

## Abstract

**Background:**

Enhancer dysregulation is one of the important features for cancer cells. Enhancers enriched with H3K4me3 have been implicated to play important roles in cancer. However, their detailed features and regulatory mechanisms have not been well characterized.

**Results:**

Here, we profile the landscape of H3K4me3-enriched enhancers (m3Es) in 43 pairs of colorectal cancer (CRC) samples. M3Es are widely distributed in CRC and averagely possess around 10% of total active enhancers. We identify 1322 gain variant m3Es and 367 lost variant m3Es in CRC. The target genes of the gain m3Es are enriched in immune response pathways. We experimentally prove that repression of CBX8 and RPS6KA5 m3Es inhibits target gene expression in CRC. Furthermore, we find histone methyltransferase MLL1 is responsible for depositing H3K4me3 on the identified Vm3Es. We demonstrate that the transcription factor AP1/JUN interacts with MLL1 and regulates m3E activity. Application of a small chemical inhibitor for MLL1 activity, OICR-9429, represses target gene expression of the identified Vm3Es, enhances anti-tumor immunity and inhibits CRC growth in an animal model.

**Conclusions:**

Taken together, our study illustrates the genome-wide landscape and the regulatory mechanisms of m3Es in CRC, and reveals potential novel strategies for cancer treatment.

**Supplementary Information:**

The online version contains supplementary material available at 10.1186/s13059-023-03108-3.

## Background

Epigenetic dysregulation is critical for chromatin stability, tumorigenesis and metastasis in cancer cells [1–3]. Currently, inhibitors for epigenetic enzymes are under clinical trials or laboratory development for histone methyltransferase, demethylases and histone modification readers [4–7]. Epigenetic marks on chromatin are important signatures for cell identity, which co-operate with transcription factors to regulate transcription [8–10]. Active and silent enhancers are marked with different patterns of histone modifications and recently widely studied in transcriptional regulation of cancer cells [10–14]. Patterns of histone modifications are considered as critical indicators for enhancer activity on chromatin. H3K4me1 marks primed enhancers; H3K27ac for active enhancers and H3K27me3 for poised enhancers [9, 11, 15]. Nowadays, H3K27ac ChIP-Seq has been widely used for identification of active enhancers in cells and tissues [8, 16, 17]. Transcribing genes are often regulated by multiple enhancers and their states vary in different cell types [11, 13]. Therefore, it is critical to determine how enhancer activity is regulated for signaling pathways and selective gene transcription.

Gain of enhancer activity has been hypothesized to occur in cancer cells [8, 18–20], supported by recent studies in patient and animal models [17, 21, 22]. However, it is still not clear whether it is a common feature for all the cancers or just a portion of them. Interestingly, many genes related with enhancer activity have been shown frequently mutated in cancer, such as *lysine methyltransferase 2C/D* (*KMT2C/D*), *E1A binding protein p300* (*EP300)*, *CREB binding protein* (*CREBBP*), *lysine demethylase 6A* (*KDM6A*, also named as *UTX*) and *lysine demethylase 5C* (*KDM5C*), which solidifies the importance of enhancer regulation in cancer [19, 23–27]. Classically, only H3K4me1 or me2 was believed to exist on enhancers; however, recently H3K4me3 has been repeatedly observed on active enhancers [11, 22, 23, 28]. Lysine methyltransferase 2B (KMT2B, also known as MLL2), has been reported to catalyze H3K4me3 on enhancers in breast cancer cells [28]. KDM5C, also known as JARID1C or SMCX, is responsible to remove H3K4me3 to keep the normal functions of enhancers [23, 29–31]. If KDM5C is mutated or repressed, H3K4me3 is enriched on enhancers and causes over activation of target genes [23, 32]. In breast cancer, KDM5C acts as a tumor suppressor through regulating enhancer function, which is targeted by an oncogenic ubiquitin E3 ligase, tripartite motif containing 11 (TRIM11) [23, 33]. However, it is still not clear whether H3K4me3-enriched enhancers (m3Es for short) exist in other cancer types, and whether other proteins are involved.

Colorectal cancer (CRC) is one of the most common cancers in the world. Recent studies have used aberrant DNA methylation of certain genes for CRC early diagnosis [34–39]. Several genome-wide studies have recently shown the importance of enhancer dynamics in CRC [40, 41]. The early studies used H3K4me1 as a mark which was not suitable to identify functional active enhancers [40]. Recently, we have systematically analyzed the distribution of active enhancers in CRC clinical samples using H3K27ac ChIP-Seq [42], and shown that enhancer reprogramming is critical for colitis-associated cancer in a mouse model [43]. Della Chiara et al*.* have studied the chromatin state in organoids derived from CRC patient tissues [44]. All these largely extended our understanding of enhancer biology in CRC. Here, to elucidate the roles of m3Es in CRC, we analyzed their distribution by combining H3K4me3 and H3K27ac ChIP-Seq data. We found that m3Es are widely distributed on the genome in human tissues and critical for controlling inflammatory gene expression in CRC.

## Results

### H3K4me3 enhancers are widely distributed in CRC tissues

**Fig. 1:**
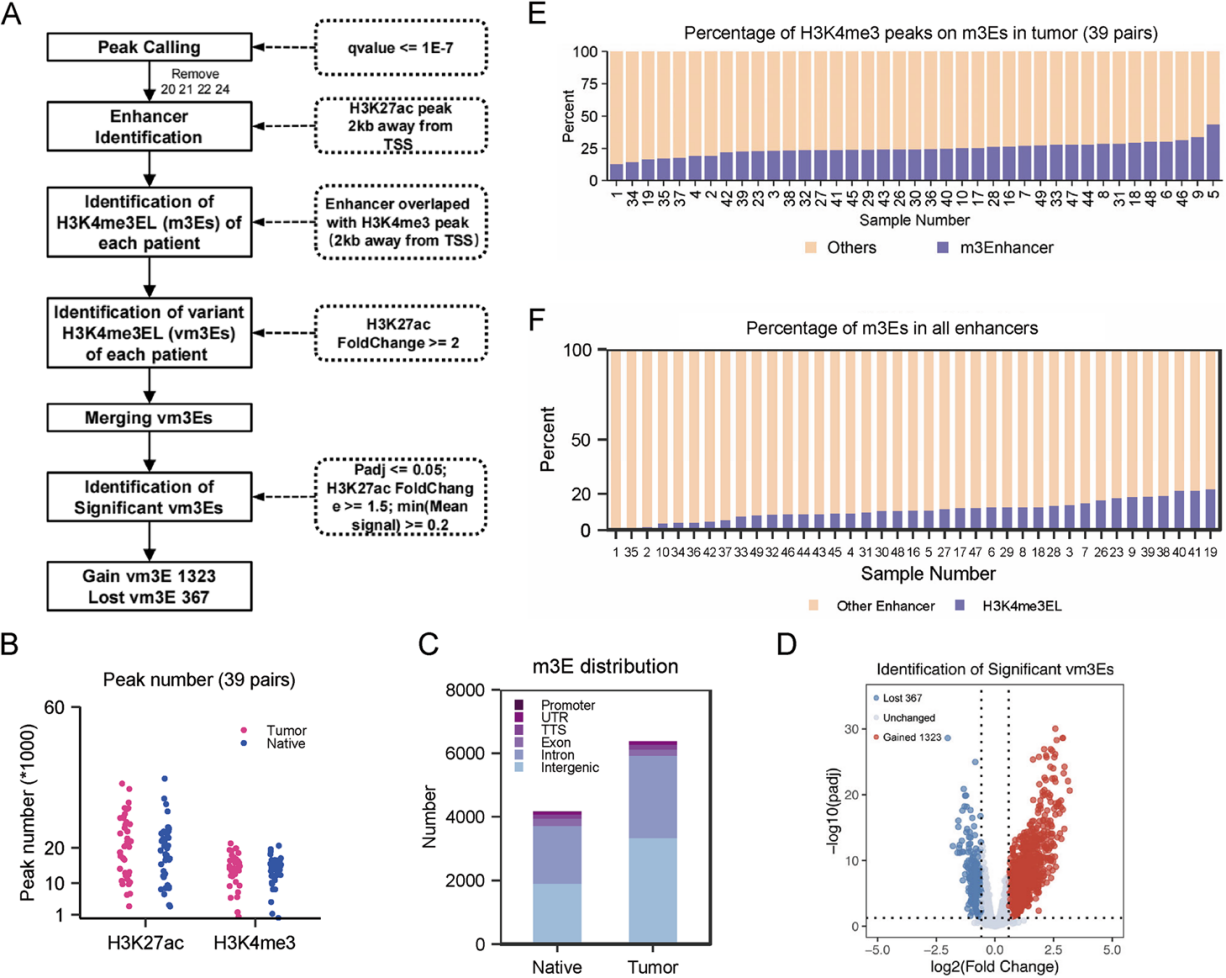
Identification of m3Es and variant m3Es. **A** The pipeline for identification of m3Es and variant m3Es in tumor tissues. **B** The peak numbers of H3K27ac and H3K4me3 in all native and tumor tissues. **C** The numbers and the genomic distribution of the identified m3Es in tumor and native tissues. **D** The numbers of the identified gain and lost m3Es in tumor tissues. **E** The percentage of H3K4me3 peaks on the identified m3Es in all H3K4me3 peaks of each tumor tissue. **F** The percentage of the identified m3Es in all enhancers of each tumor tissue

To investigate whether H3K4me3 enhancers exist in CRC, we performed ChIP-Seq analysis of H3K27ac and H3K4me3 with 43 pairs of CRC adjacent and tumor tissue samples (Additional file 1: Fig. S1A-C) [42]. We first identified active enhancers based on H3K27 peaks 2 kb away from transcription start site (TSS), and then overlapped them with H3K4me3 peaks 2 kb away from TSS (Fig. 1A). Totally we analyzed 43 samples. In 4 samples, their peak numbers were much less than the other, and we considered their sequencing data probably were not good enough, and excluded them in the following analysis (Fig. 1B). The total number of identified m3Es were 4165 in the adjacent tissues and 6385 in tumor tissues, and totally 7438 m3Es were identified (Fig. 1C, Additional file 1: Fig. S1D). Most of the m3Es were distributed in the intron and intergenic regions, like typical enhancers (Fig. 1C). The expression of target genes for m3Es is higher than those for typical enhancers (TEs) but lower than those for super enhancers (SEs) (Additional file 1: Fig. S1E). For most of the identified m3Es, their length is shorter than 1 kb, similar as TEs (Additional file 1: Fig. S1F). Comparing the tumor tissues with native tissues, totally 1689 variant m3Es (Vm3E for short) in CRC were identified, among which 1323 were gain variant m3Es (gain Vm3E) and 367 were lost variant m3Es (lost Vm3E) (Fig. 1A & D, Additional file 2: Table S1). The H3K4me3 peaks on enhancers were around 6% of total H3K4me3 peaks in all samples (Fig. 1E), and the amount of m3Es is around 10% of total enhancers in all cancer tissues (Fig. 1F). Considering the large amount of the identified m3Es, they may play important roles in CRC.

### Characterization of variant H3K4me3 enhancers in CRC

**Fig. 2:**
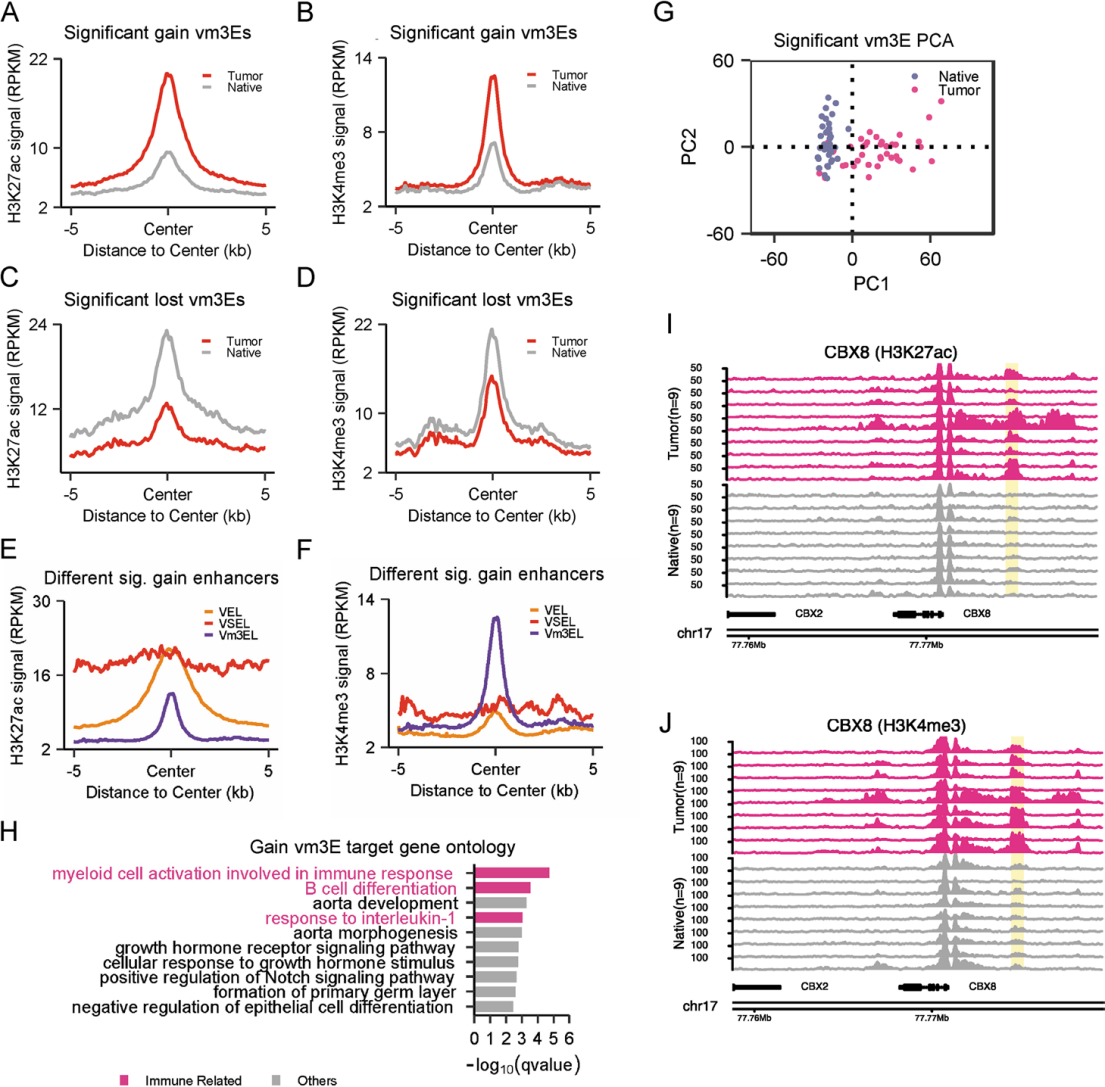
The variant m3Es in CRC are enriched in genes regulating immune response. **A** & **B** Relative H3K27ac (**A**) and H3K4me3 (**B**) signals of gain m3Es in all tumor tissues. **C** & **D** Relative H3K27ac (**C**) and H3K4me3 (**D**) signals of lost m3Es in all tumor tissues. **E** & **F** Relative H3K27ac (**E**) and H3K4me3 (**F**) signals of gain typical enhancers, super enhancers and m3Es in tumor tissues. **G** PCA analysis using all identified significant Vm3Es classified tumor and native tissue into distinct clusters. **H** Human disease ontology analysis for the target genes of gain Vm3Es using GREAT (version 3.0). The pink bar represents cancer related disease and the grey bar represents other disease. **I** & **J** Representative H3K27ac (**I**) and H3K4me3 (**J**) tracks of gain m3E close to *CBX8* gene. The Vm3E is highlighted in yellow

To investigate the roles of Vm3Es in CRC, we first analyzed the histone modifications on them. Both H3K27ac and H3K4me3 signals on the gain m3Es were higher in tumor than native tissues; while both signals on the lost m3Es were lower (Fig. 2A-D). Since we identified Vm3Es based on the difference of H3K27ac (Fig. 1A), our results suggest certain correlation exists between H3K4me3 and H3K27ac on these enhancers. Interestingly, when looking at the gain and lost Vm3Es in each patient, some patients almost do not have Vm3Es, implying Vm3Es are quite patient-specific (Additional file 1: Fig. S2A). Then we compared the signals of variant typical enhancers, super enhancers and H3K4me3 enhancers. The results indicated that the H3K27ac signal of Vm3Es was lower than that of variant typical enhancers and super enhancers; while the H3K4me3 signal of Vm3Es was higher than the other two (Fig. 2E & F), indicating that Vm3Es have distinct features and regulatory mechanisms. The target genes of gain Vm3Es showed significant higher fold changes than those of gain variant enhancers (Additional file 1: Fig. S2B), implying the gain Vm3Es may have higher activity in promoting transcription. We used all Vm3Es to perform PCA analysis and successfully distinguished tumor tissues from adjacent tissues, implying Vm3Es may be useful for tumor classification (Fig. 2G).

### Gain H3K4me3 enhancers in CRC are associated with immune genes

GO analysis for the target genes of gain Vm3Es showed that they were associated with colorectal cancer, and enriched in immune response and growth signaling pathway (Fig. 2H, Additional file 1: Fig. S2C). *CBX8*, encoding one subunit of PRC1 and involved in cancer and immune cell infiltration [45–47], is presented to show the elevated H3K4me3 and H3K27ac on its enhancer in tumor tissues (Fig. 2I & J). The m3Es for several well-known oncogenes, *CCND1*, *PTSG2*, *VEGFA* and *CNTN3*, are shown in Additional file 1: Fig. S3.

### Validation of gain H3K4me3 enhancers

**Fig. 3:**
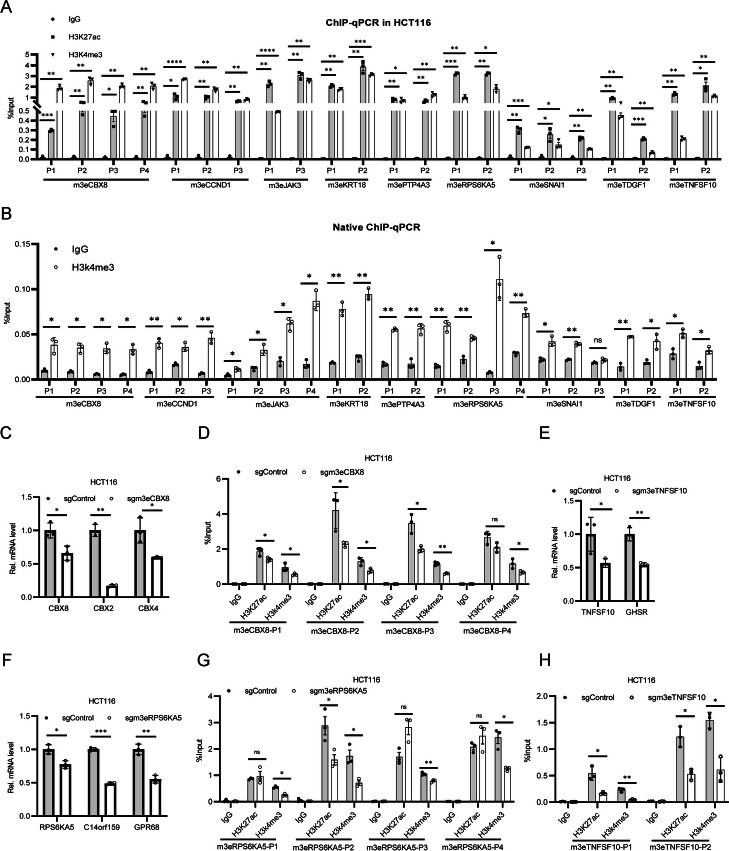
Validation of the identified Vm3Es and their roles in transcription regulation. **A** ChIP-qPCR showing H3K27ac and H3K4me3 levels at m3Es in HCT116. **B** Native ChIP-qPCR showing the H3K4me3 level at m3Es in HCT116. **C**-**E** Bar plot showing the relative mRNA level of the proximal genes for m3Es in control and sgRNA groups (*n* = 3). sgRNA targeting EGFP was used as the control in the following experiments. **F**–**H** ChIP-qPCR showing H3K27ac and H3K4me3 level at *CBX8*, *RPS6KA5*, *TNFSF10* enhancer loci in control and sgRNA groups. * means *p* value < 0.05, ** for *p* value < 0.01. Data are represented as mean ± SD or SEM. *p*-values are calculated by two-tailed unpaired t test

After identifying the Vm3Es in CRC, we then performed ChIP-PCR to validate them in colon cell lines. After comparing the ChIP-Seq data from tissues and cell lines, we selected m3Es for 9 genes (*CBX8*, *CCND1*, *JAK3*, *KRT18*, *PTP4A3*, *RPS6KA5*, *SNAI1*, *TDGF1* and *TNFSF10*), and examined H3K4me3 and H3K27ac on them in HCT116 and RKO colon cell lines. The ChIP-grade antibody was validated in the early study [22], and two or three sets of PCR primers were designed for each enhancer. The results confirmed that H3K27ac and H3K4me3 are enriched on these enhancers (Fig. 3A, Additional file 1: Fig. S4A). Considering that H3K4me3 on TSS may interfere the results via promoter-enhancer loops after crosslinking, native ChIP assays were performed. The results showed that H3K4me3 signal was significantly enriched on most of the sites examined (Fig. 3B, Additional file 1: Fig. S4B), supporting that these m3Es do exist in colon cancer cells.

### Repression of gain H3K4me3 enhancers inhibits target gene expression

To investigate the functions of the identified Vm3Es in CRC, we used a CRISPR-KRAB system to repress enhancer activity [48]. Two sgRNAs were designed for each enhancer and stable cell lines derived from HCT116 were constructed for the following experiments. Infection of viruses containing CRISPR-KRAB and sgRNA successfully repressed the expression of their target genes, and H3K27ac and H3K4me3 modifications were also reduced (Fig. 3C-H, Additional file 1: Fig. S5). Interestingly, we examined multiple proximal genes to the Vm3Es, and found that often most of the examined genes were repressed (Fig. 3C-H, Additional file 1: Fig. S5), suggesting that these Vm3Es can control the expression of multiple proximal genes.

### Repression of gain H3K4me3 enhancers inhibits CRC tumor growth

**Fig. 4:**
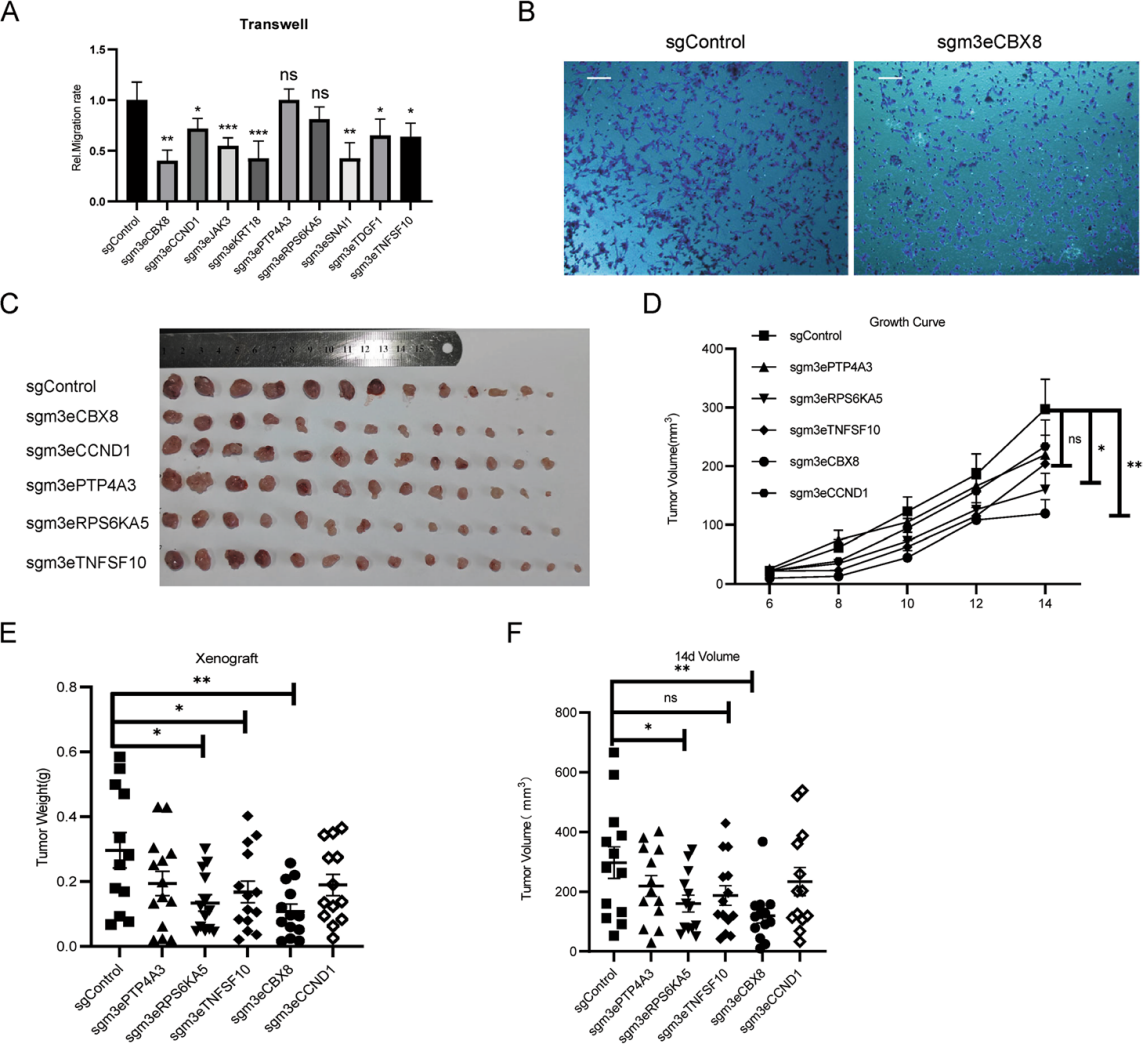
Roles of m3Es in CRC. **A** Transwell assays for HCT116 cell lines stably transfected with dCas9-KRAB sgRNAs targeting the indicated m3Es. **B** Representative images of transwell migration assay of HCT116 cell lines stably transfected with dCas9-KRAB m3eCBX8. Scale bar, 50um. **C**-**F** Xenograft experiments in nude mice were performed with HCT116 stable cells expressing the indicated sgRNAs. The tumors were pictured (**C**), and their growth curve (**D**), weight (**E**) and volume (**F**) are shown. *n* = 13 for all groups. Data are presented as mean values ± SEM. Statistical analysis was performed using a two-sided student t test. *p* value was labelled on the corresponding items

To investigate whether the above Vm3Es are important for CRC, we first examined the cell proliferation and migration ability in the above stable cell lines. The results of CKK-8 assay showed that repression of the above enhancers caused a subtle difference in cell proliferation, but not significant enough (Additional file 1: Fig. S6A & B). The cell cycle analysis using flow cytometry did not show significant difference among these cell lines either (Additional file 1: Fig. S6C & D). These indicate that repression of above Vm3Es did not cause significant difference in proliferation. Then we checked the migration ability using transwell assay. The results showed that repression of m3Es for *CBX8*, *CCND1*, *JAK3*, *KRT18*, *SNAI1*, *TDGF1* and *TNFSF10* caused a significant reduction in migration (Fig. 4A & B). Then we injected the stable cell lines containing m3E sgRNAs for *CBX8*, *CCND1*, *PTP4A3*, *RPS6KA5* and *TNFSF10* into nude mice and checked whether tumor growth was repressed. The results show that repression of m3Es proximal to *CBX8* (m3eCBX8) and *RPS6KA5* (m3eRPS6KA5) caused significant inhibition of tumor volume and weight; and repression of m3eTNFSF10 caused significant reduction of tumor weight but not tumor volume (Fig. 4C-F). All these indicate that m3eCBX8 and m3eRPS6KA5 play important functions in CRC.

### MLL1/KMT2A are required for H3K4me3 on enhancers

**Fig. 5:**
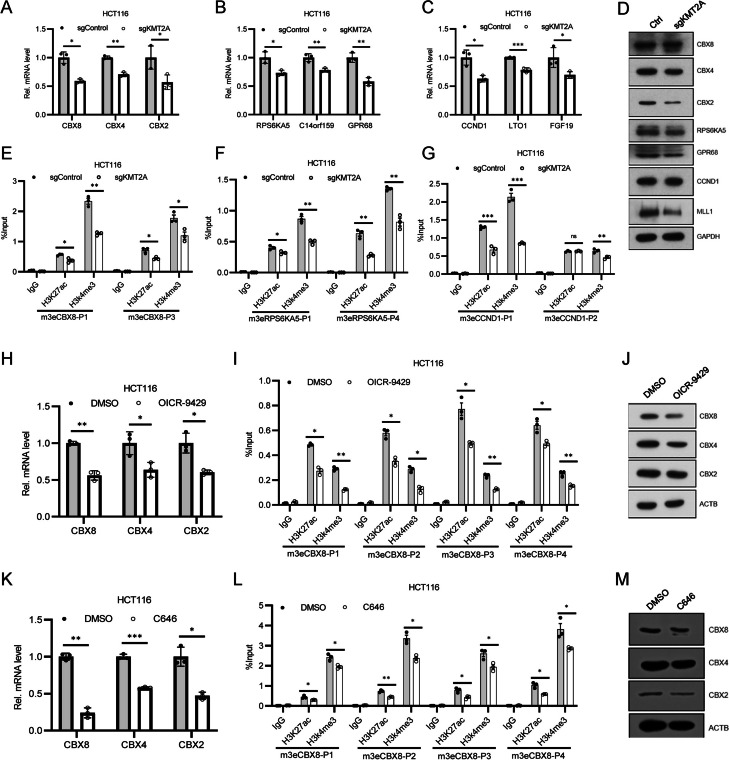
MLL1/KMT2A regulates H3K4me3 on m3Es. **A**-**C** Bar plot showing the relative mRNA levels of the proximal genes for CBX8, RPS6KA5, CCND1 m3Es in *KMT2A*-KD HCT116 cells. **D** Western blot detection of the protein level of the proximal genes for CBX8, RPS6KA5, CCND1 m3Es in *KMT2A*-KD HCT116 cells. **E**–**G** ChIP-qPCR assessing H3K27ac and H3K4me3 level at CBX8, RPS6KA5, CCND1 m3Es in *KMT2A*-KD cell line. **H** & **I** Bar plot showing the mRNA levels of the proximal genes and histone modifications of CBX8 m3E in HCT116 after OICR-9429 (10 µM-48 h) treatment. **J** Western blotting of the proximal genes for CBX8 m3E after OICR-9429 (10 µM, 48 h) treatment. **K** & **L** Bar plot showing the mRNA levels of the proximal genes and histone modifications of CBX8 m3E in HCT116 after C646 (10uM-12 h) treatment. **M** Western blotting of the proximal genes for CBX8 m3E after C646 (10 µM, 12 h) treatment. * means *p* value < 0.05, ** for *p* value < 0.01. Data are represented as mean ± SD or SEM. *p*-values are calculated by two-tailed unpaired t test

After proving the functions of m3Es in CRC, we further studied the mechanisms regulating their activity. To identify the methyltransferase responsible for H3K4me3 on enhancers, we screened seven major H3K4 methyltransferases, KMT2A-E and SETD1A/B. Stable knockdown cell lines for each gene were constructed with CRISPR/Cas9 system, and the expression of m3E target genes were examined. Among them, *MLL1/KMT2A* knockdown caused the reduction of almost all tested genes (Additional file 1: Fig. S7A). Knockdown of other genes caused changes of a portion of tested genes (Additional file 1: Fig. S7B-H), but none of them showed comparable effect to *MLL1/KMT2A* knockdown. The results of *CBX8*, *RPS5KA5* and *CCND1* were then confirmed with RT-PCR and western blotting (Fig. 5A-D); and ChIP-PCR results showed that MLL1 knockdown successfully decreased H3K4me3 on the corresponding m3Es (Fig. 5E-G). Thus, we concluded that MLL1/KMT2A should be the major enzyme for H3K4me3 on the tested m3Es.

### Enhancer repression by small molecule chemicals inhibits its activity

To further explore the role of histone modifications in regulating m3Es, we applied a small molecule inhibitor for MLL1, OICR-9429, which targets the interaction between MLL1 and WDR5 and disrupts MLL1 complex [49]. OICR-9429 treatment repressed the expression of *CBX8*, *CBX4* and *CBX2*, and decreased H3K4me3 level on m3eCBX8 (Fig. 5H-J). Interestingly, we found that H3K27ac was also decreased after drug treatment (Fig. 5I). Similar results were observed for other genes (Additional file 1: Fig. S8). Previously, we also observed that H3K27ac on m3Es decreased together with H3K4me3 after *MLL1* knockdown (Fig. 5E-G). These suggest that on m3Es, H3K4me3 change affects H3K27ac level. Then we treated the cells with C646, an inhibitor for H3K27 acetylase p300/CBP, and investigate the effect of H3K27ac on H3K4me3. C646 treatment also inhibited the expression of *CBX8*, *CBX4* and *CBX2* (Fig. 5K-M), and repressed H3K27ac on the enhancers, which led to H3K4me3 reduction (Fig. 5L). Similar results were observed for other genes (Additional file 1: Fig. S9).

### AP-1/JUN transcription factor is enriched on H3K4me3 enhancers

**Fig. 6:**
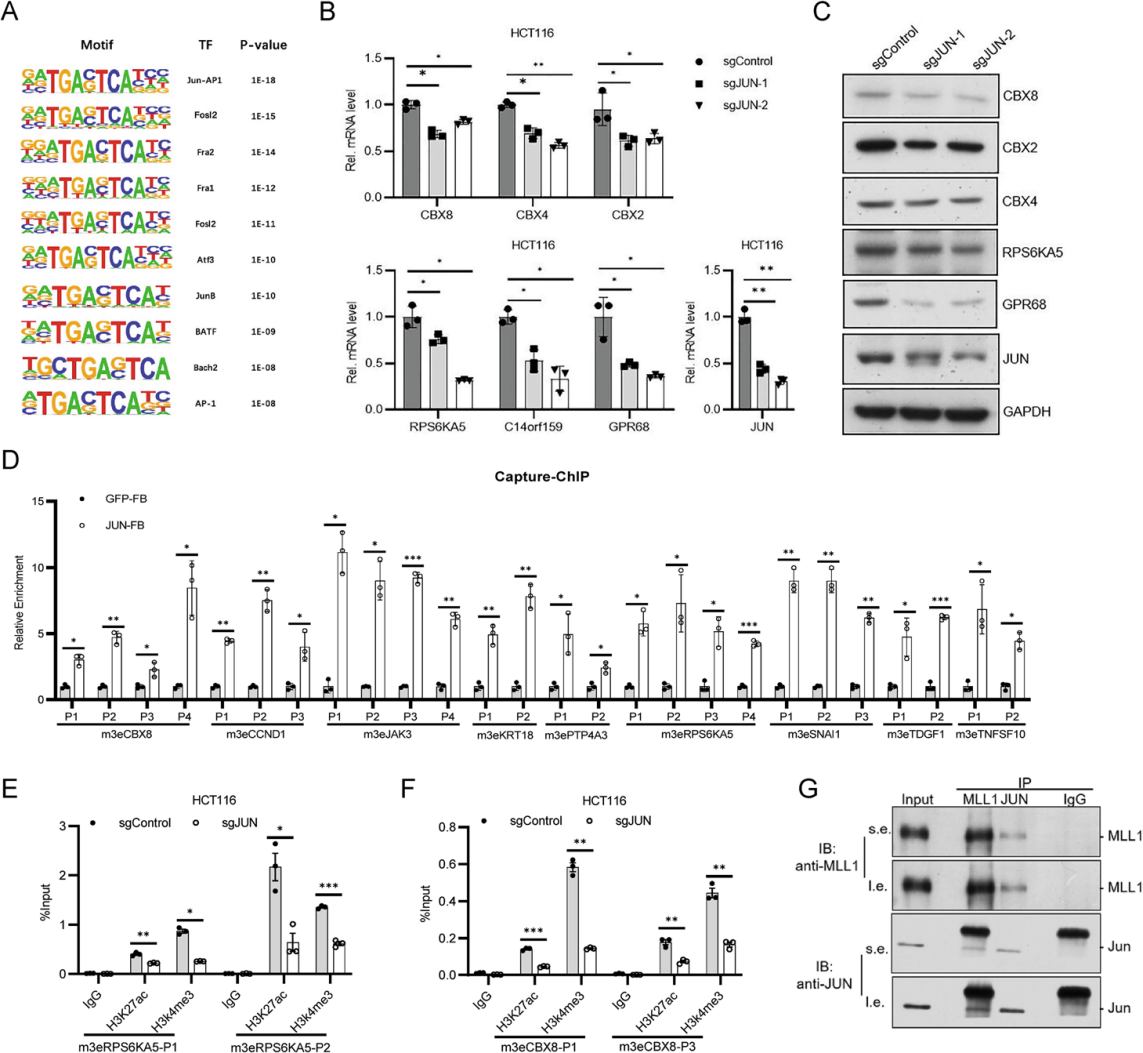
JUN regulates m3Es and interacts with MLL1. **A** DNA motifs enriched within nucleosome-free regions (NFRs) of gain Vm3Es determined by HOMER motif analysis. **B** Bar plot showing the relative mRNA level of the proximal genes for *RPS6KA5* and *CBX8* enhancers in *JUN*-KD HCT116 cell line. **C** Western blot of the proximal genes for *RPS6KA5* and *CBX8* m3Es in HCT116 with *JUN* knockdown. **D** Capture-ChIP qPCR showing the enrichment of JUN at m3Es in *JUN*-overexpressed HCT116. **E** & **F** ChIP-qPCR assessing H3K27ac and H3K4me3 level at RPS6KA5, CBX8, CCND1 m3Es in *JUN*-KD HCT116 cell line. **G** Western blot of MLL1 or JUN after co-immunoprecipitation (co-IP) with indicated antibodies in HCT116. * means *p* value < 0.05, ** for *p* value < 0.01. Data are represented as mean ± SD or SEM. *p*-values are calculated by two-tailed unpaired t test

To investigate the transcription factors enriched on Vm3Es, we performed motif analysis and found the top hits were mostly AP-1 family members (Fig. 6A). To verify the function of AP-1 on m3Es, we knocked down *JUN* with shRNA in HCT116 cells, and found that *JUN* deficiency caused down-regulation of the genes targeted by the nine Vm3Es (Fig. 6B, C & Additional file 1: Fig. S10A-D). To study whether JUN binds to the identified m3Es, we constructed a HCT116-derived stable cell line with FB-tagged JUN. The results of Capture-ChIP showed that FB-JUN was enriched on the m3Es for *CBX8*, *CCND1*, *JAK3*, *KRT18*, *PTP4A3*, *RPS6KA5*, *SNAI1*, *TDGF1* and *TNFSF10* (Fig. 6D). *JUN* knockdown caused H3K4me3 down-regulation on the enhancers, and decreased H3K27ac on most of examined sites (Fig. 6E & F, Additional file 1: Fig. S10E-J). Since both JUN and MLL1 regulate H3K4me3 on the above m3Es, we investigated whether the two proteins interacted with each other. Co-immunoprecipitation was performed with anti-MLL1 or anti-JUN antibodies, which showed that JUN interacts with MLL1 (Fig. 6G). These together indicate that JUN and MLL1 interact with each other and regulate histone modifications on m3Es.

### Correlation of JUN and m3Es in a CRC animal model

To further investigate the relationship of JUN and m3Es, we took the epigenomic dataset from an azoxymethane (AOM)-dextran sodium sulfate (DSS) induced CRC mouse model [43], which contained H3K4me3 and H3K27ac ChIP-Seq data at five time points, including control, 2-week, 4-week, 7-week and 10-week (Fig. S11A). 2-week and 4-week time points represented the inflammation stage, and 7-week and 10-week for tumor stage [43]. The numbers of enhancers and m3Es were shown in Fig. S11B. We analyzed the mRNA level for *Jun* from the corresponding RNA-Seq data (Fig. S11C), and obtained information of Jun binding sites from GEO database (GSE73372). Then we analyzed the presence of Jun around m3Es, and found that Jun was distributed on most of the identified m3Es at 10-week (Fig. S11D). We also found that the percentage of m3Es bound by Jun in total m3Es showed the similar trend as Jun expression; as comparison, the percentage of typical enhancers bound by Jun in total enhancers showed a different trend (Fig. S11E). These implicated that Jun is localized on m3Es in the mouse model and it is associated with m3Es during tumorigenesis.

### Regulation of anti-tumor immunity by MLL1 inhibitor OICR-9429

**Fig. 7:**
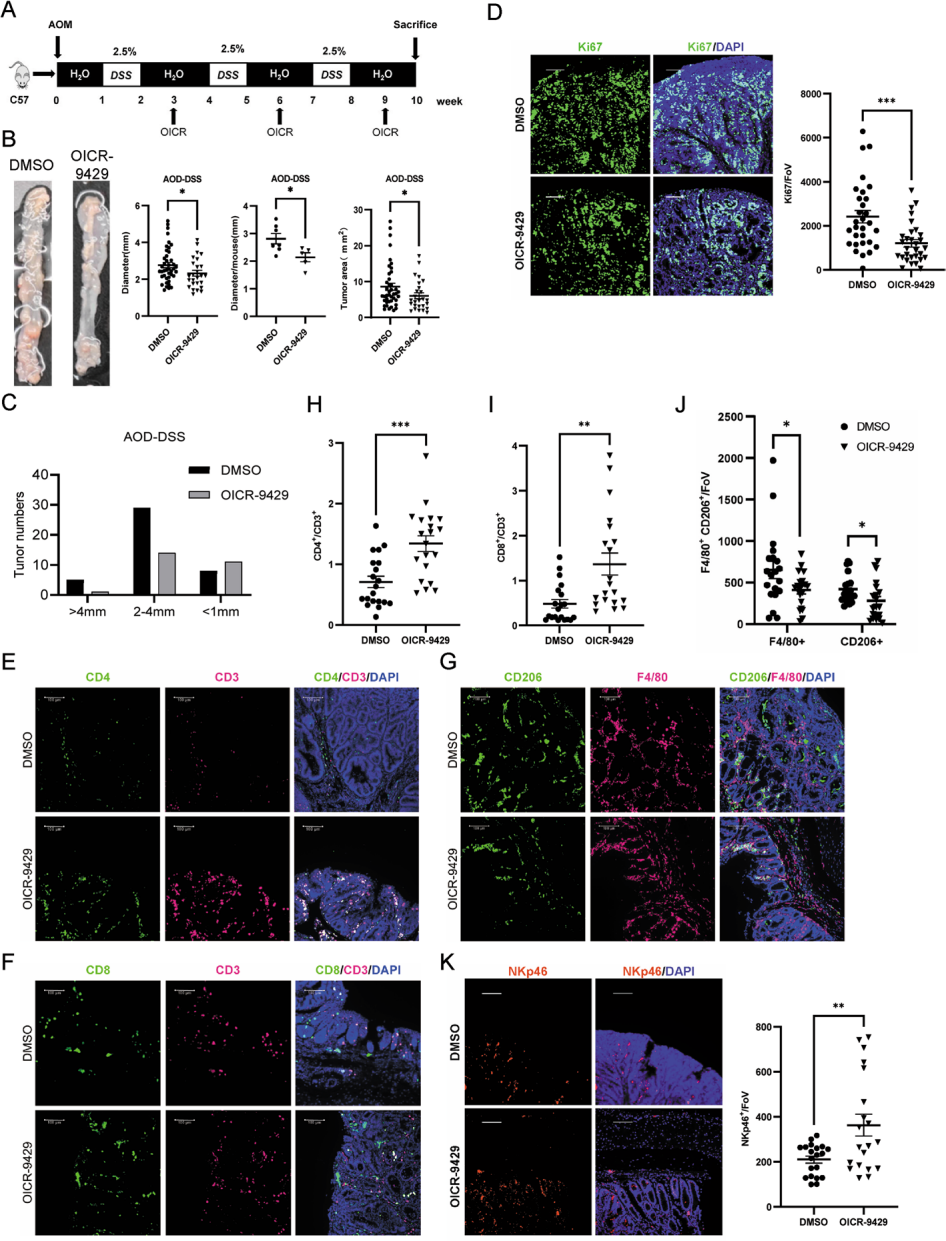
Inhibition of MLL1 by OICR-9429 repressed CRC and regulated tumor immunity. **A** The sketch for CRC animal model treated with OICR-9429. **B** The representative image for colons with/without OICR-9429. Tumors were measured, and the total tumor number and size for each animal was plotted in a line for the DMSO and OICR-9429 groups. **C** The histogram showing the tumor size distribution. **D** Representative confocal images and quantitation of tumor stained with Ki67 in AOM-DSS induced CRC tumors. *n* = 30 FoV. **E**–**G** Representative confocal micrographs of CD3^+^CD4^+^ T helper (**E**), CD3^+^CD8^+^ T cytotoxic lymphocytes (**F**) and F4/80^+^CD206^+^ M2 type of TAM (tumor-associated macrophage) (**G**). **H**-**J** Quantification of CD3^+^CD4^+^ T helper (**H**), CD3^+^CD8^+^ T cytotoxic lymphocytes (**I**), and F4/80^+^CD206^+^ M2 TAM (**J**). *n* = 20 FoV. **K** Representative confocal micrographs and quantification of NK cell marker NKp46 in AOM-DSS induced CRC tumors. *n* = 20 FoV. * means *p* value < 0.05, ** for *p* value < 0.01. Data are represented as mean ± SEM. *p*-values are calculated by two-tailed unpaired t test

To further explore whether it is feasible to treat CRC through manipulating m3E activity, we used OICR-9429, an inhibitor of MLL1 complex, to treat CRC cells. OICR-9429 treatment significantly repressed CRC cell proliferation and caused G1/S cell cycle arrest (Fig. S12A-C). OICR-9429 also repressed the growth of organoids derived from CRC patient tissues (Fig. S12D & E). Then we applied OICR-9429 in an AOM/DSS-induced CRC animal model (Fig. 7A). The body weight of each mice was measured during the whole process and no significant difference was observed between control and drug groups (Fig. S12F). OICR-9429 significantly repressed the size and number of AOM/DSS-induced tumor (Fig. 7B & C). Ki-67 staining also indicated that OICR-9429 inhibited tumor cell proliferation (Fig. 7D). RT-PCR results indicated that OICR-9429 treatment significantly repressed the expression of most of the genes targeted by the identified Vm3Es in the intestine and spleen tissues (Fig. S12G & H). Since the target genes of the identified Vm3Es were enriched in immune response, we investigated whether OICR-9429 affected immune cell infiltration in tumors. The results of immune staining indicated that the amount of CD4 + and CD8 + T cells increased in the OICR-9429-treated CRC tissues, as well as the NK cells labelled by NKp46, while CD206-labelled M2 macrophage decreased (Fig. 7E-J). The results suggested that OICR-9429 can enhance anti-tumor immunity in CRC tumor tissues, which is probably associated with its activity in regulating m3Es.

## Discussion

It has been quite a few years since it was reported that H3K4me3 was found on enhancers. However, many scientists worried that the result might be artificial because of antibody cross activity or contamination from H3K4me3 signal on promoters. The features and functions of m3Es have not been well characterized because of the existent debate. In our previous studies, we tested multiple H3K4me3 antibodies from different companies [22], and in the current study, we used native ChIP approach to avoid interfering signal from promoter H3K4me3. All these results indicate that enhancers enriched with H3K4me3 exist in mammalian cells. We have further shown that m3Es possess around 10% of the total enhancers, indicating that m3Es are widely distributed in the genome and play important functions in the cells.

Our functional analysis of gain Vm3Es in CRC showed that their target genes are mostly enriched in genes related with immune response. Evasion from immune survey is one of the important characters of tumor cells and immune therapy has shown promising effects in multiple cancer types [50]. Our data indicate that tumor cells alter the expression pattern of immune genes through m3Es, and promote tumor growth and development. It may be useful to combine immune therapy together with enhancer manipulation in cancer treatment. We have also found that AP-1/JUN transcription factor is involved in the regulation of m3E activity. It is well known that AP-1/JUN is tightly associated with immune pathways and acts as an oncogene in multiple cancers. Recently, multiple epigenomic studies and motif analyses based on enhancer profiling have predicted and proved AP-1 family as one of the most critical families of oncogenic transcription factors [42, 51–53]. Considering the important functions of AP-1 members in cancer, immunity and stress response, our study has established the potential links among AP-1, tumor-specific Vm3Es, inflammation and cancer which might be critical for tumorigenesis and metastasis.

Our results showed that repression of m3eCBX8 and m3eRPS6KA5 inhibited CRC tumor growth in the animal model, suggesting m3Es play important roles in corectal cancer, and probably in other cancer. They exhibit relative higher activity in comparison with typical enhancers, and some of them are capable to regulate the expression of multiple genes. The tumor-specific m3Es may become new targets for diagnosis and drug treatment. It has been reported recently that CBX family members are associated with immune cell infiltration in CRC [45, 46]. *RPS6KA5* has been implicated to be involved in immune response of lung cancer [54]. Then the two m3Es are possibly involved in the immune response regulated by OICR-9429. m3eCBX8 controls the expression of three CBX family members, CBX2/4/8. Other identified Vm3Es are also capable to regulate multiple proximal genes. Usually it is difficult to manipulate multiple genes in vivo. Our data suggest that it might be feasible to control multiple gene expression through targeting one enhancer, which might be useful for experiment and drug design.

It is known that MLL1 is involved in the transcription of E2F transcription factors and regulates cell cycle. Then the effects of OICR-9429 on cells and organoids may rely on MLL1’s function in cell cycle. Our further study about OICR-9429 in the AOM/DSS-induced CRC model indicated that repression of Mll1 complex up-regulated anti-tumor immune response in CRC, supporting that MLL1 activity on m3Es is associated with tumor immunity in CRC. We also examined the effects of JNK/JUN inhibitors; however, we did not get consistent results. It was maybe due to the complicated functions of JNK/JUN in multiple pathways. Nevertheless, we have discovered that it is useful to manipulate m3E activity in treating cancer, and it will be interesting for the future studies to reveal the exact function of each m3E identified in the current analysis.

## Conclusions

In the current study, we analyze the genome-wide landscape of m3Es in CRC and show that they are widely distributed on the chromatin. The CRC specific gain m3Es are tightly associated with cancer and immune pathways, and repression of m3Es close to *CBX8* (m3eCBX8) and *RPS6KA5* (m3eRPS6KA5) significantly impairs the growth of the xenograft tumors. MLL1 and AP-1 co-operate to regulate the activity of the gain Vm3Es in CRC, and OICR-9429, an inhibitor of MLL1, can repress CRC via regulate tumor immunity. Thus, our study illustrates the genome-wide landscape and important functions of m3Es in CRC, and reveals potential novel strategies for CRC treatment.

## Methods

### Reagents and cell lines

Antibodies recognizing H3K4me3 (Millipore, 04–745, RRID: AB_1163444; CST, 9751, RRID:AB_2616028), H3K27ac (Abcam, ab4729, RRID: AB_2118291), MLL1 (CST, 14,197, RRID:AB_2688010), JUN (CST, 9165, RRID:AB_2130165), CD3 (Abcam, ab16669, RRID:AB_443425), CD4 (Abcam, ab183685, RRID:AB_2686917), CD8 (Abcam, ab217344, RRID:AB_2890649), F4/80(CST, 70,076, RRID:AB_2799771), CD206 (Proteintech, 60143–1-Ig, RRID:AB_2144924), NKp46 (Abcam, ab233558, RRID:AB_2904203), Ki67(Abcam, ab15580, RRID:AB_443209) were purchased from indicated commercial sources. Protein G-Sepharose beads (GE Healthcare), AOM (MP Biomedicals, 183971), DSS (MP Biomedicals, 160110), CBX8 (ABclonal Cat# A6222, RRID:AB_2766831), CBX2 (ABclonal Cat# A3294, RRID:AB_2863036), CBX4 (ABclonal Cat# A5109, RRID:AB_2863448), RPS6KA5 (ABclonal Cat# A5699, RRID:AB_2766458), GPR68 (ABclonal Cat# A7348, RRID:AB_2767885), CCND1 (ABclonal Cat# A19038, RRID:AB_2862530), JAK3 (ABclonal Cat# A0748, RRID:AB_2757376), KRT18 (ABclonal Cat# A19778), PTP4A3(ABclonal Cat# A2004, RRID:AB_2764028), SNAI1 (ABclonal Cat# A11794, RRID:AB_2758760), TNFSF10 (ABclonal Cat# A2138, RRID:AB_2764157), ACTB ( ABclonal Cat# AC038, RRID:AB_2863784), GAPDH (ABclonal Cat# AC033, RRID:AB_2769570) were purchased from the indicated companies. PCR primers were custom synthesized by Tsingke Biotechnology and siRNAs by GenePharma. HEK293T, HCT116 and RKO Cell lines were purchased from Cell Bank of Chinese Academy and cultured under recommended conditions according to the manufacturer’s instruction with 10% FBS. Cell lines were verified by the merchant before shipping, and no mycoplasma was detected.

### Animal housing and ethics approval

The BALB/C nude mice and C57BL/6 J mice were purchased from GemPharmatech Co., Ltd. All the mice were born and maintained under pathogen-free condition at 20 ~ 24℃ with a humidity of 40 ~ 70% and a 12/12-h dark/light cycle (lights on at 7:00 AM, lights off at 7:00 PM), with free access of water and food (Animal Center of College of Life Sciences, Wuhan University).

### Generation of colitis-associated colorectal cancer mice model

The 8-week-old C57BL/6 J mice were randomly divided into two experimental groups. Mice in the experimental groups were given a single intraperitoneal injection of AOM (10 mg/kg body weight). Seven days after the AOM injection, the mice were given 2.5% DSS (w/v) in drinking water for 7 days, and then fed with distilled water for 14 days, which was repeated at 4-week and 7-week. The mice were intraperitoneally injected with DMSO or OICR-9429 (5 mg/kg) at 3-week, 6-week, and 9-week (three time per week). All the mice were sacrificed at 10-week. The colorectum tissues were divided to distal, middle, proximal fragments to anus, and only the proximal fragments were collected for experiments.

### ChIP assay

ChIP assay was performed as previously described [55, 56]. Briefly, cells were cross-linked with 1% formaldehyde for 10 min at room temperature and quenched with 0.125 M glycine for 5 min. Cross-linked cells were washed twice with PBS, then collected by centrifugation. Then cells were re-suspended with the ChIP digestion buffer (50 mM of Tris–HCl, pH 8.0, 1 mM of CaCl2, 0.2% Triton X-100). Chromatin was digested to 150- 300 bp by MNase (M0247S, NEB) at 37 °C for 20 min and quenched with EDTA. Supernatant was collected and equally divided after diluted with five times of dilution buffer (20 mM of Tris–HCl, pH 8.0, 150 mM of NaCl, 2 mM of EDTA, 1% Triton X-100, 0.1% SDS). Samples were then incubated with protein G beads and antibodies at 4 °C overnight. Next day, the beads were washed once with wash buffer I (20 mM Tris–HCl, pH 8.0, 150 mM NaCl, 2 mM EDTA, 1% Triton X-100, 0.1% SDS), once with wash buffer II (20 mM Tris–HCl pH 8.0, 500 mM NaCl, 2 mM EDTA, 1% Triton X-100, 0.1% SDS), once with wash buffer III (10 mM Tris–HCl pH 8.0, 250 mM LiCl, 1 mM EDTA, 1% Na-deoxycholate, 1% NP-40) and twice with TE (10 mM Tris–HCl pH 8.0, 1 mM EDTA). The beads were eluted twice with 100 μL elution buffer (1% SDS, 0.1 M NaHCO3, 0.2 mg/ml Proteinase K (Biosharp)) at room temperature. The elution was incubated at 65 °C for 6 h and then purified with DNA purification kit (TIANGEN DP214-03). The purified DNA was assayed by quantitative PCR with quantitative PCR. Primers for ChIP-qPCR were in Additional file 3: Table S2.

### Native ChIP

Briefly, 2 × 10^7^ cells were centrifuged at 3,000 rpm for 5 min at 4℃. After washing them once with cold PBS, re-suspend the cells in nuclear extraction buffer (10 mM of Tris, pH 8.0, 10 mM NaCl, 0.2% NP-40, proteinase inhibitor cocktail (1x), 1 mM DTT and fresh PMSF) for 30 min at 4℃. Nuclei was collected by centrifuge and resuspended in MNase digestion buffer (50 mM Tris–HCl, pH7.6, 1 mM CaCl2, 0.2% Triton X-100 or NP-40, proteinase inhibitor cocktail (1x), 1 mM DTT and fresh PMSF). Chromatin was digested by MNase (M0247S, NEB) at 37℃ for 20 min and quenched with EDTA to a final concentration of 5 mM. Supernatant was collected and equally divided after diluted with five times of dilution buffer (20 mM of Tris–HCl, pH 8.0, 150 mM of NaCl, 2 mM of EDTA, 1% Triton X-100, 0.1% SDS). Samples were then incubated with protein G beads and antibodies at 4℃ overnight. Next day, the beads were washed once with wash buffer I, II, III, and the following procedures were performed as described in ChIP assay. Primer information was in Additional file 3: Table S2.

### Capture-ChIP

2 × 10^7^ FB-EGFP or FB-JUN/BirA-expressing HCT116 or RKO stable cells were harvested, cross-linked with 2 mM SMCC (MCE) for 45 min and 0.2% formaldehyde for 10 min at room temperature, and then quenched with 0.125 M glycine for 5 min. Cross-linked cells were washed twice with PBS, then collected by centrifugation. Cells were lysed in 1 mL RIPA buffer (10 mM Tris–HCl pH 8.0, 1 mM EDTA, 0.1% sodium deoxycholate, 0.1% SDS, 1% Triton X-100), and rotated for 15 min at 4℃. Cell lysates were centrifuged at 2,300 g for 5 min at 4℃ to isolate the nuclei. Nuclei were suspended in 500 μl of 0.5% SDS lysis buffer (0.5% SDS, 10 mM EDTA, 50 mM Tris–HCl, pH 8.0) and subjected for sonication to shear chromatin fragments to 200—500 bp. Fragmented chromatin was centrifuged at 16,100 g for 10 min at 4℃. 450 mL of supernatant was transferred to a new tube and NaCl solution was added to the final concentration of 300 mM. Supernatant was then incubated with 10 μl of Dynabeads MyOne streptavidin C1 (Thermo-Fisher Scientific) at 4 ℃ overnight. Then the Dynabeads were washed twice with 2% SDS, twice with RIPA buffer containing 0.5 M NaCl, twice with LiCl buffer (250 mM LiCl, 0.5% NP-40, 0.5% sodium deoxycholate, 1 mM EDTA and 10 mM Tris–HCl, pH 8.0), and twice with TE buffer (10 mM Tris–HCl, 1 mM EDTA, pH 8.0). The chromatin was eluted in SDS elution buffer (1% SDS, 10 mM EDTA, 50 mM Tris–HCl, pH 8.0) followed by incubation at 65℃ overnight. DNA was treated with RNase A (5 mg/ml, Sigma) and protease K (0.2 mg/ml, Biosharp) at 37℃ for 30 min, and extracted with DNA purification kit (TIANGEN). The purified DNA was assayed by quantitative PCR with quantitative PCR. Primers for ChIP-qPCR were listed in Additional file 3: Table S2.

### Library preparation for ChIP-sequencing

ChIP-seq libraries were constructed with ChIP and input DNA using VATHS Universal DNA Library Prep Kit for Illumina (Vazyme ND606). Briefly, 50 μL of DNA (8–10 ng) was end-repaired for dA tailing, followed by adaptor ligation. Each adaptor was marked with a barcode of 8 bp DNA. Adaptor-ligated DNA was purified by AMPure XP beads (1:1) and then amplified by PCR of 9 cycles with the primer matching with adaptor universal part. Amplified DNA was purified again using AMPure XP beads (1:1) in 35 μL EB elution buffer. For multiplexing, libraries with different barcodes were mixed with equal molar quantities (30–50 million reads per library). Libraries were sequenced by Illumina Nova-seq platform with pair-end reads of 150 bp.

### RNA-sequencing

RNA extraction was performed using Ultrapure RNA Kit (CWBIO, CW0581M). Briefly, around 40 mg tissues were triturated for 30 s in 1 mL TRIzon provided in the kit, incubated at room temperature for 5 min, added with 200 μL chloroform and shaken drastically. After centrifugation at 12,000 rpm, 4 °C for 10 min, the upper water phase was moved into an adsorption column provided by the kit. The column was then eluted with 50 μL RNase-free water. RNA-seq libraries were constructed by NEBNext Poly(A) mRNA Magnetic Isolation Module (NEB E7490) and NEBNext Ultra II Non-Directional RNA Second Strand Synthesis Module (NEB E6111). mRNA was purified with poly-T magnetic beads and first and second strand cDNA was synthesized. The resulted cDNA was purified by AMPure XP beads (1:1) and eluted in 50 μL nucleotide-free water. The subsequent procedures were the same as described in ChIP-seq library construction, except that the sequencing depth was 20 million reads per library. RNA-seq libraries were sequenced by Illumina Nova-seq platform with pair-end reads of 150 bp.

### ChIP-seq data analysis

ChIP-seq raw fastq data were quality controlled using FastQC (version 0.11.9, https://www.bioinformatics.babraham.ac.uk/projects/fastqc/). Clean data were obtained by removing adapter with fastp (version 0.21.0, https://github.com/OpenGene/fastp, parameters were “-f 5 -t 20 -F 5 -T 20 -l 30”). Cleaned reads were aligned in paired-end mode to homo sapiens UCSC reference genome hg19 with BWA mem (version 0.7.12, http://bio-bwa.sourceforge.net) [57]. Duplicate reads were removed by picard Markduplicates (version 2.18.29, https://broadinstitute.github.io/picard/). All mapped reads were used in the analysis. Peaks were called by MACS2 (version 2.1.1, https://github.com/taoliu/MACS, parameters “–nomodel -p 1E-7 -B –broad –extsize 147”) [58]. For the comparison in enhancers, signal of modification in each enhancer was normalized by reads per kilobase per million mapped reads (RPKM).

### RNA-seq data analysis

RNA-seq clean data were obtained same as ChIP-seq data (parameters were “-f 5 -t -20 -F 5 -T -20 -l 30”). Quality control was done with FastQC (version 0.11.9, https://www.bioinformatics.babraham.ac.uk/projects/fastqc/) and Multiqc (version 1.10.1, https://multiqc.info). Clean reads were aligned to homo sapiens UCSC hg19 genome with STAR (version 2.7.9, https://github.com/alexdobin/STAR) [59]. Gene annotation was done using UCSC hg19 genome. The gene expression level was normalized as fragments per kilobase of bin per million mapped reads (FPKM).

### Identification of VELs (Variant enhancer loci)

To identify the significant variant enhancer loci between native and tumor tissues, we first identified all VELs in paired native and tumor tissues, defined as enhancers whose H3K27ac fold change (FC) >  = 2 between native and tumor tissues. We merged all VELs into one single coordinate file, and calculated the recurrence and significance (Benjamini & Hochberg corrected *p*-value) for all VELs. We used recurrence of 8 and 9 as significance threshold for gain and lost VELs, respectively, because gain and lost VELs achieved the significant percentage cut-off (0.90) when recurrence larger than these numbers.

### Identification of H3K4me3-enriched enhancers

To identify the significant v3mEs between native and tumor tissue, we first identified active enhancers based on H3K27 peaks 2 kb away from transcription start site (TSS), and then overlapped them with H3K4me3 peaks 2 kb away from TSS.

These enhancers were defined as m3Es. Then we identified all v3mEs in paired native and tumor tissues. V3mEs of Individual samples were defined as m3Es whose H3K27ac fold change (FC) >  = 2 between native and tumor tissues. We merged all m3Es into one single coordinate file, and calculated the mean H3K27ac signal and normalized to RPKM. We used fold change (FC) of 1.5 as significance threshold for Vm3Es.

### Identification of VSELs

For variant super-enhancer loci (VSEL), the identifying procedure was similar as described above in “Identification of VELs”. We used recurrence of 5 as significance threshold for both gain and lost VELs.

### Transcript factor enrichment

We used the findMotifsGenome.pl module in HOMER (version 4.11, http://homer.ucsd.edu/homer/, parameters were -size 200 -mask) to identify transcript factor motifs.

### Human disease ontology and Gene Ontology analysis

The coordinate file of gain and lost Vm3Es were submitted to GREAT website (version 3.0.0) and the results of human disease ontology and GO analysis (biological process) were obtained for plotting.

### ChIP-Seq and RNA-Seq data visualization

UCSC genomic track for histone marks, RNA expression and chromatin state beyond the RefSeq gene model were drew by karyoploteR [60], using the alignment file of chromatin markers and state annotation bed files produced by ChromHMM. Histone marker’s signal density panel across TSS and gene body were plotted by in house R script, using the density matrix data produced by deeptools (version 3.3.2) [61].

### Reverse transcription and quantitative PCR

Cells were scraped down and collected with centrifugation. For tissue RNA extraction, 20 mg of tissues were homogenized and collected with centrifugation. Total RNA was extracted with RNA extraction kit (Aidlab or CWBIO) according to the manufacturer’s manual. Approximately 1 μg of total RNA was used for reverse transcription with a first strand cDNA synthesis kit (Toyobo). The resulted cDNA was assayed with quantitative PCR, β-actin for normalization. The sequences of primers are in Additional file 3: Table S2. Assays were repeated at least three times. Data were shown as average values ± SD or SEM of at least three representative experiments. P value was calculated using student’s t test.

### Cell proliferation assay

The cell proliferation was measured using the CCK-8 assay. Briefly, 1,000 cells were seeded into 96-well plates. The cells were added with Cell Counting Kit-8 (CCK-8) solution (10 μL; Yeasen) in each well at the indicated time points before incubation for 1—4 h at 37℃, followed by analysis via microplate reader. The absorbance was measured at 450 nm. Assays were repeated at least three times. Data were shown as average values ± SD of at least three representative experiments and *p* value was calculated using student’s t test.

### Immunoprecipitation and immunoblot

Cells were harvested and lysed in NP40 lysis buffer (50 mM Tris, pH 7.4, 150 mM NaCl, 0.5% NP40) in the presence of proteinase inhibitors. After removing insoluble particles, the supernatant was incubated with protein G beads (GE Healthcare) and specific antibody at 4℃ for 4 h. The beads were spin down and washed three times with lysis buffer. Then SDS loading buffer was added to the beads to release proteins for SDS-PAGE and Western blotting. To prepare cell lysates for western blotting, cells lysates were prepared with SDS lysis buffer (50 mM Tris–HCl pH 6.8, 4%SDS). Lysates were separated by SDS-PAGE and transferred to nitrocellulose filter membranes blocked with 5% milk and incubated with primary antibody overnight at 4℃. Then the blots were washed three times in TBS-T, incubated with secondary antibodies at room temperature for 1 h, and detected by Clarity Western ECL Substrate (BIO-RAD).

### Cell cycle analysis with flow cytometry

Cells at the logarithmic phase were harvested after digestion with 0.05% Trypsin–EDTA, washed twice with cold PBS and immobilized with ice-cold 70% ethanol overnight. Fixed cells were washed twice with PBS and stained in PBS containing propidium iodide (PI, 50 μg/mL, MCE) and RNase (100 μg/mL, Sigma) for 30 min at 37 °C. Data was generated using cytoflex flow cytometer (Beckman Coulter) and analyzed using FlowJo V10.8.1 software.

### Transwell migration assay

1 × 10^5^ HCT116 cells were loaded per transwell insert (24-well insert; pore size, 8 µm; BD Biosciences) without serum or growth factors, and medium supplemented with 10% fetal bovine serum was used as a chemoattractant in the lower chamber. After 36 h of incubation, cells at the underside of the membrane were fixed in methanol for 10 min and stained with crystal violet (0.1% in 20% EtOH for 20 min). After washing, remaining cells in the insert were removed by a cotton swab. Migrated cells were visualized by light microscopy and counted with ImageJ. Assays were repeated at least three times. Data were shown as average values ± SD of at least three representative experiments and *p* value was calculated using student’s t test.

### Generation of knockout/knockdown cell line with CRISPR/ Cas9 system

The single guide RNA (sgRNA) sequences were designed by using the CRISPR Design Tool (http://tools.genomeengineering.org), provided by Feng Zhang lab. The target sequences sgRNAs were shown in Supplementary Table 2. The sgRNAs were cloned in lentiCRISPRv2-puro (Addgene, #98,290). To construct knockdown cell lines, the lentiviral particles were generated by transfecting HEK293T cells with CRISPR plasmid. Then the supernatant was used to infect the desired cells, and cells were selected by puromycin.

### CRISPR-Cas9-KRAB mediated repression of variant H3K4me3 enhancers

Site-specific sgRNAs targeting enhancer loci were designed with publicly available filtering tools (https://zlab.bio/guide-design-resources) to minimize off-target cleavage. The target sequences sgRNAs were shown in Supplementary Table 2. For CRISPR interference, sgRNAs were cloned into pLH-spsgRNA2 (Addgene, #64,114) through the BbsI site according to the protocol recommended by Addgene. Lentivirus was generated by transfecting HEK293T cells with sgRNA expression cocktails or pHAGE dCas9-KRAB-MeCP2, together with helper plasmids, psPAX and pMD2G. Medium containing virus was collected at 48 or 72 h after transfection, and filtered with 0.45 μm filters (Millipore). Stable cell lines were generated by infecting HCT116 or RKO with lentivirus expressing dCas9-KRAB-MeCP2 and sgRNAs, and then screened with puromycin and hygromycin for 48 h.

### Immunohistochemistry

CRC tissues were resected and fixed in 4% paraformaldehyde for overnight, dehydrated, embedded in paraffin, and sectioned. Tissue Sects. (5 μm) were used for staining. For immunohistochemistry (IHC) detection, tissue sections were deparaffinized, rehydrated, and treated for heat-mediated antigen retrieval. Tumor sections incubated with the primary antibodies against: anti-Ki67, anti-CD3, anti-CD4, anti-CD8, anti-F4/80, anti-CD206, and anti-NKp46 over night at 4 °C. After the primary incubation sections were washed and incubated with Alexa lour 488 or Cy3-labelled secondary antibodies for one hour at room temperature, tumor sections were washed three times and counter stained with DAPI and mount with Fluoromount G. Immunofluorescence images were pictured using Leica Thunder Image DMI8 microscope at 20X magnification and analyzed using ImageJ software.

### Isolation and culture of organoids

For derivation of tumoroids, surgically resected colorectal tissues were washed with ice-cold PBS-Abs buffer (phosphate-buffered saline with antibiotic, 1% penicillin/streptomycin, and 100 μg/ml Primocin), and then chopped into 1 mm pieces in ice-cold PBS. The fragments were transferred to 15 ml tube and resuspended with 10 ml PBS, then filtered through a 40 μm cell strainer on a 50 ml tube. The tissues from the strainer were transferred to a 15 mL tube, and digested in digestion medium (advanced DMEM/F12 with 2% FBS, collagenase type II (2.5 mg/ml), collagenase type IV (2.5 mg/ml), and DNaseI (0.1 mg/ml)) at 37 °C. After digestion, cells were filtered through 70 μm cell strainer on a 50 ml tube, crypt-containing fraction was collected by centrifugation, and embedded in Matrigel (R&D). After the Matrigel balls were polymerized, human isolation medium (HIM) was added. HIM is composed of advanced DMEM/F-12 (GIBCO) supplemented with Glutamax (GIBCO), HEPES (GIBCO), B-27 (GIBCO), N-2 (GIBCO), 10 mM nicotinamide (Sigma), 1 mM N-acetyl-L-cysteine (Sigma), 2 μM A8301 (Tocris), 10 nM [Leu15]-gastrin (Sigma), Y-27632 (10 μM, MCE), 100 ng/mL human noggin (MCE), 50 ng/mL human EGF (MCE), 500 ng/ml R-Spondin1 (MCE), SB202190 (10 μM, MCE), CHIR99021(5 μM, MCE) and Blebbistatin (10 μM, MCE). After the first 7 days, isolation medium was changed with human expansion medium (HEM), containing HIM but not Noggin, R-Spondin1 and Y-27632. For passage of organoids, organoids were released from the Matrigel by cell-recovery solution (Corning), dissociated into single cells using TrypLE express (Gibco), and resuspended with Matrigel and divided into a 24-well plate (50 μl per well). After the Matrigel balls were polymerized, 500 μl of HEM was added, and media was changed on organoids every 2–3 days.

### Xenograft experiments in mice

Five-week-old male BALB/C nude mice were purchased from GemPharmatech Co., Ltd. Colon cancer model was established by injecting subcutaneously 7.5 × 10^5^ HCT116 cells per site into the flank regions of mice. Tumor volumes were measured every 2 days using calipers. Tumor volumes were calculated as V = 0.5 × length × width^2^. After 14 days of injection, tumors were harvested and weighed. Data were shown as mean ± SEM and *p* values were calculated by the student’s t test.

### Statistics and reproducibility

For experiments other than NGS sequencing, at least three biological replicates for each experiment were performed. Data are presented as mean values ± SEM. Statistical analysis was performed using a two-sided Student t test. *p* value was either labelled on the corresponding items or listed in the legends.

### Supplementary Information

**Additional file 1: Supplementary Figure S1-S12. Fig. S1.** Quality control of sequencing data. **Fig. S2.** Functional analysis of gain and lost Vm3Es in CRC. **Fig. S3.** Gene browser view of representative gain Vm3Es in CRC. **Fig. S4.** Validation of identified Vm3Es. **Fig. S5.** Inhibition of m3Es represses target gene expression. **Fig. S6.** Functions of the identified Vm3Es on cell proliferation and cell cycle. **Fig. S7.** Screen of methyltransferases regulating m3E target genes. **Fig. S8.** OICR-9429 treatment repressed the identified m3Es. **Fig. S9.** C646 treatment repressed histone modifications on the identified Vm3Es. **Fig. S10.** JUN regulates the identified Vm3Es in CRC. **Fig. S11.** JUN binds to H3K4me3 enhancer loci in the AOM-DSS induced CRC model. **Fig. S12.** OICR-9429 treatment repressed CRC.

**Additional file 2: Table S1.** Information of gain and lost vm3Es in CRC.

**Additional file 3: Table S2.** List of primers and oligonucleotides used in this study.

**Additional file 4.** Uncropped images for the blots in Figure 5-6, Fig S5 and Fig S7-10.

**Additional file 5.** Review history.

## Data Availability

Publicly available ChIP-Seq data for CRC patients used in this study were obtained from GEO with the accession number GSE156614 (https://www.ncbi.nlm.nih.gov/geo/query/acc.cgi?acc=GSE156614) [62], and the epigenomic datasets for AOM/DSS-induced CRC mouse model were obtained from GEO with the accession number GSE178145 (https://www.ncbi.nlm.nih.gov/geo/query/acc.cgi?acc=GSE178145) [63] and the accession number GSE178144 (https://www.ncbi.nlm.nih.gov/geo/query/acc.cgi?acc=GSE178144) [64], the Jun ChIP-seq data was obtained from GEO with the accession number GSE73372 (https://www.ncbi.nlm.nih.gov/geo/query/acc.cgi?acc=GSE73372) [65]. The source code and the scripts used for data analysis are deposited in Github https://github.com/Wangchenyu5005/crc_h3k4m3Enhancer_1.git under a GPL-3 license [66] and are freely available from Zenodo (https://doi.org/10.5281/zenodo.10117358) [67]. The microscopy images used for quantification in Fig. 7 are deposited at Figshare (https://doi.org/10.6084/m9.figshare.24523762.v1) [68]. All the reagents and materials presented in this paper are available from the authors upon request.

## References

[1] Morgan MA, Shilatifard A (2015). Chromatin signatures of cancer. Genes Dev.

[2] Brookes E, Shi Y (2014). Diverse epigenetic mechanisms of human disease. Annu Rev Genet.

[3] Sharma S, Kelly TK, Jones PA (2010). Epigenetics in cancer. Carcinogenesis.

[4] Ellis L, Atadja PW, Johnstone RW (2009). Epigenetics in cancer: targeting chromatin modifications. Mol Cancer Ther.

[5] Jones PA, Issa JP, Baylin S (2016). Targeting the cancer epigenome for therapy. Nat Rev Genet.

[6] Morel D, Jeffery D, Aspeslagh S, Almouzni G, Postel-Vinay S (2020). Combining epigenetic drugs with other therapies for solid tumours - past lessons and future promise. Nat Rev Clin Oncol.

[7] Dawson MA, Kouzarides T (2012). Cancer epigenetics: from mechanism to therapy. Cell.

[8] Hnisz D, Abraham BJ, Lee TI, Lau A, Saint-Andre V, Sigova AA, Hoke HA, Young RA (2013). Super-enhancers in the control of cell identity and disease. Cell.

[9] Herz HM, Mohan M, Garruss AS, Liang K, Takahashi YH, Mickey K, Voets O, Verrijzer CP, Shilatifard A (2012). Enhancer-associated H3K4 monomethylation by Trithorax-related, the Drosophila homolog of mammalian Mll3/Mll4. Genes Dev.

[10] Calo E, Wysocka J (2013). Modification of enhancer chromatin: what, how, and why?. Mol Cell.

[11] Shlyueva D, Stampfel G, Stark A (2014). Transcriptional enhancers: from properties to genome-wide predictions. Nat Rev Genet.

[12] Nizovtseva EV, Todolli S, Olson WK, Studitsky VM (2017). Towards quantitative analysis of gene regulation by enhancers. Epigenomics.

[13] Rickels R, Shilatifard A (2018). Enhancer logic and mechanics in development and disease. Trends Cell Biol.

[14] Medina-Rivera A, Santiago-Algarra D, Puthier D, Spicuglia S (2018). Widespread enhancer activity from core promoters. Trends Biochem Sci.

[15] Wang C, Lee JE, Lai B, Macfarlan TS, Xu S, Zhuang L, Liu C, Peng W, Ge K (2016). Enhancer priming by H3K4 methyltransferase MLL4 controls cell fate transition. Proc Natl Acad Sci U S A.

[16] Dowen JM, Fan ZP, Hnisz D, Ren G, Abraham BJ, Zhang LN, Weintraub AS, Schujiers J, Lee TI, Zhao K, Young RA (2014). Control of cell identity genes occurs in insulated neighborhoods in mammalian chromosomes. Cell.

[17] Roe JS, Hwang CI, Somerville TDD, Milazzo JP, Lee EJ, Da Silva B, Maiorino L, Tiriac H, Young CM, Miyabayashi K (2017). Enhancer reprogramming promotes pancreatic cancer metastasis. Cell.

[18] Murakawa Y, Yoshihara M, Kawaji H, Nishikawa M, Zayed H, Suzuki H, Fantom C, Hayashizaki Y (2016). Enhanced identification of transcriptional enhancers provides mechanistic insights into diseases. Trends Genet.

[19] Yao J, Chen J, Li LY, Wu M (2020). Epigenetic plasticity of enhancers in cancer. Transcription.

[20] Flavahan WA, Gaskell E, Bernstein BE (2017). Epigenetic plasticity and the hallmarks of cancer. Science.

[21] Yuan J, Jiang YY, Mayakonda A, Huang M, Ding LW, Lin H, Yu F, Lu Y, Loh TKS, Chow M (2017). Super-enhancers promote transcriptional dysregulation in nasopharyngeal carcinoma. Cancer Res.

[22] Li QL, Wang DY, Ju LG, Yao J, Gao C, Lei PJ, Li LY, Zhao XL, Wu M (2019). The hyper-activation of transcriptional enhancers in breast cancer. Clin Epigenetics.

[23] Shen H, Xu W, Guo R, Rong B, Gu L, Wang Z, He C, Zheng L, Hu X, Hu Z (2016). Suppression of enhancer overactivation by a RACK7-histone demethylase complex. Cell.

[24] Wang L, Shilatifard A (2019). UTX mutations in human cancer. Cancer Cell.

[25] Fagan RJ, Dingwall AK (2019). COMPASS Ascending: Emerging clues regarding the roles of MLL3/KMT2C and MLL2/KMT2D proteins in cancer. Cancer Lett.

[26] Sze CC, Shilatifard A (2016). MLL3/MLL4/COMPASS family on epigenetic regulation of enhancer function and cancer. Cold Spring Harb Perspect Med.

[27] van Haaften G, Dalgliesh GL, Davies H, Chen L, Bignell G, Greenman C, Edkins S, Hardy C, O'Meara S, Teague J (2009). Somatic mutations of the histone H3K27 demethylase gene UTX in human cancer. Nat Genet.

[28] Hu D, Gao X, Cao K, Morgan MA, Mas G, Smith ER, Volk AG, Bartom ET, Crispino JD, Di Croce L, Shilatifard A (2017). Not all H3K4 methylations are created equal: Mll2/COMPASS dependency in primordial germ cell specification. Mol Cell.

[29] Iwase S, Lan F, Bayliss P, de la Torre-Ubieta L, Huarte M, Qi HH, Whetstine JR, Bonni A, Roberts TM, Shi Y (2007). The X-linked mental retardation gene SMCX/JARID1C defines a family of histone H3 lysine 4 demethylases. Cell.

[30] Outchkourov NS, Muino JM, Kaufmann K, van Ijcken WF, Groot Koerkamp MJ, van Leenen D, de Graaf P, Holstege FC, Grosveld FG, Timmers HT (2013). Balancing of histone H3K4 methylation states by the Kdm5c/SMCX histone demethylase modulates promoter and enhancer function. Cell Rep.

[31] Scandaglia M, Lopez-Atalaya JP, Medrano-Fernandez A, Lopez-Cascales MT, Del Blanco B, Lipinski M, Benito E, Olivares R, Iwase S, Shi Y, Barco A (2017). Loss of Kdm5c causes spurious transcription and prevents the fine-tuning of activity-regulated enhancers in neurons. Cell Rep.

[32] Chen X, Loo JX, Shi X, Xiong W, Guo Y, Ke H, Yang M, Jiang Y, Xia S, Zhao M (2018). E6 protein expressed by high-risk HPV activates super-enhancers of the EGFR and c-MET oncogenes by destabilizing the histone demethylase KDM5C. Cancer Res.

[33] Xiao Q, Wang CY, Gao C, Chen JD, Chen JJ, Wang Z, Ju LG, Tang SB, Yao J, Li F (2022). Regulation of KDM5C stability and enhancer reprogramming in breast cancer. Cell Death Dis.

[34] Tse JWT, Jenkins LJ, Chionh F, Mariadason JM (2017). Aberrant DNA methylation in colorectal cancer: what should we target?. Trends Cancer.

[35] Hinoue T, Weisenberger DJ, Lange CP, Shen H, Byun HM, Van Den Berg D, Malik S, Pan F, Noushmehr H, van Dijk CM (2012). Genome-scale analysis of aberrant DNA methylation in colorectal cancer. Genome Res.

[36] Sahnane N, Magnoli F, Bernasconi B, Tibiletti MG, Romualdi C, Pedroni M, de PonzLeon M, Magnani G, Reggiani-Bonetti L, Bertario L (2015). Aberrant DNA methylation profiles of inherited and sporadic colorectal cancer. Clin Epigenetics.

[37] Wang HY, Long QY, Tang SB, Xiao Q, Gao C, Zhao QY, Li QL, Ye M, Zhang L, Li LY, Wu M (2019). Histone demethylase KDM3A is required for enhancer activation of hippo target genes in colorectal cancer. Nucleic Acids Res.

[38] Yao J, Lei PJ, Li QL, Chen J, Tang SB, Xiao Q, Lin X, Wang X, Li LY, Wu M (2020). GLIS2 promotes colorectal cancer through repressing enhancer activation. Oncogenesis.

[39] Jagle S, Busch H, Freihen V, Beyes S, Schrempp M, Boerries M, Hecht A (2017). SNAIL1-mediated downregulation of FOXA proteins facilitates the inactivation of transcriptional enhancer elements at key epithelial genes in colorectal cancer cells. PLoS Genet.

[40] Akhtar-Zaidi B, Cowper-Sal-lari R, Corradin O, Saiakhova A, Bartels CF, Balasubramanian D, Myeroff L, Lutterbaugh J, Jarrar A, Kalady MF (2012). Epigenomic enhancer profiling defines a signature of colon cancer. Science.

[41] Cohen AJ, Saiakhova A, Corradin O, Luppino JM, Lovrenert K, Bartels CF, Morrow JJ, Mack SC, Dhillon G, Beard L (2017). Hotspots of aberrant enhancer activity punctuate the colorectal cancer epigenome. Nat Commun.

[42] Li QL, Lin X, Yu YL, Chen L, Hu QX, Chen M, Cao N, Zhao C, Wang CY, Huang CW (2021). Genome-wide profiling in colorectal cancer identifies PHF19 and TBC1D16 as oncogenic super enhancers. Nat Commun.

[43] Chen L, Luo Z, Zhao C, Li Q, Geng Y, Xiao Y, Chen MK, Li L, Chen ZX, Wu M (2022). Dynamic chromatin states coupling with key transcription factors in colitis-associated colorectal cancer. Adv Sci (Weinh).

[44] Della Chiara G, Gervasoni F, Fakiola M, Godano C, D'Oria C, Azzolin L, Bonnal RJP, Moreni G, Drufuca L, Rossetti G (2021). Epigenomic landscape of human colorectal cancer unveils an aberrant core of pan-cancer enhancers orchestrated by YAP/TAZ. Nat Commun.

[45] Li Q, Pan Y, Cao Z, Zhao S (2020). Comprehensive analysis of prognostic value and immune infiltration of chromobox family members in colorectal cancer. Front Oncol.

[46] Beguelin W, Teater M, Gearhart MD, Calvo Fernandez MT, Goldstein RL, Cardenas MG, Hatzi K, Rosen M, Shen H, Corcoran CM (2016). EZH2 and BCL6 cooperate to assemble CBX8-BCOR complex to repress bivalent promoters, mediate germinal center formation and lymphomagenesis. Cancer Cell.

[47] Tang J, Wang G, Zhang M, Li FY, Sang Y, Wang B, Hu K, Wu Y, Luo R, Liao D (2014). Paradoxical role of CBX8 in proliferation and metastasis of colorectal cancer. Oncotarget.

[48] Thakore PI, D'Ippolito AM, Song L, Safi A, Shivakumar NK, Kabadi AM, Reddy TE, Crawford GE, Gersbach CA (2015). Highly specific epigenome editing by CRISPR-Cas9 repressors for silencing of distal regulatory elements. Nat Methods.

[49] Vedadi M, Blazer L, Eram MS, Barsyte-Lovejoy D, Arrowsmith CH, Hajian T (2017). Targeting human SET1/MLL family of proteins. Protein Sci.

[50] Cercek A, Lumish M, Sinopoli J, Weiss J, Shia J, Lamendola-Essel M, El Dika IH, Segal N, Shcherba M, Sugarman R (2022). PD-1 blockade in mismatch repair-deficient, locally advanced rectal cancer. N Engl J Med.

[51] Wang CY, Yu GT, Gao C, Chen J, Li QL, Zhang L, Wu M, Sun ZJ, Li LY (2021). Genome-wide enhancer analysis reveals the role of AP-1 transcription factor in head and neck squamous cell carcinoma. Front Mol Biosci.

[52] Li XJ, Li QL, Ju LG, Zhao C, Zhao LS, Du JW, Wang Y, Zheng L, Song BL, Li LY (2021). Deficiency of histone methyltransferase SET domain-containing 2 in liver leads to abnormal lipid metabolism and HCC. Hepatology.

[53] Bejjani F, Evanno E, Zibara K, Piechaczyk M, Jariel-Encontre I (2019). The AP-1 transcriptional complex: local switch or remote command?. Biochim Biophys Acta Rev Cancer.

[54] Pei L, Liu H, Ouyang S, Zhao C, Liu M, Wang T, Wang P, Ye H, Wang K, Song C (2020). Discovering novel lung cancer associated antigens and the utilization of their autoantibodies in detection of lung cancer. Immunobiology.

[55] Zhu K, Lei PJ, Ju LG, Wang X, Huang K, Yang B, Shao C, Zhu Y, Wei G, Fu XD (2017). SPOP-containing complex regulates SETD2 stability and H3K36me3-coupled alternative splicing. Nucleic Acids Res.

[56] Wang Z, Chen J, Gao C, Xiao Q, Wang XW, Tang SB, Li QL, Zhong B, Song ZY, Shu HB (2021). Epigenetic dysregulation induces translocation of histone H3 into cytoplasm. Adv Sci (Weinh).

[57] Li H, Durbin R (2010). Fast and accurate long-read alignment with Burrows-Wheeler transform. Bioinformatics.

[58] Zhang Y, Liu T, Meyer CA, Eeckhoute J, Johnson DS, Bernstein BE, Nusbaum C, Myers RM, Brown M, Li W, Liu XS (2008). Model-based analysis of ChIP-Seq (MACS). Genome Biol.

[59] Dobin A, Davis CA, Schlesinger F, Drenkow J, Zaleski C, Jha S, Batut P, Chaisson M, Gingeras TR (2013). STAR: ultrafast universal RNA-seq aligner. Bioinformatics.

[60] Gel B, Serra E (2017). karyoploteR: an R/Bioconductor package to plot customizable genomes displaying arbitrary data. Bioinformatics.

[61] Ramirez F, Dundar F, Diehl S, Gruning BA, Manke T (2014). deepTools: a flexible platform for exploring deep-sequencing data. Nucleic Acids Res.

[62] Li QL, Lin X, Yu YL, Chen L et al. Genome-wide analysis of enhancer landscape in colorectal cancer subgroups. GSE156614, https://www.ncbi.nlm.nih.gov/geo/query/acc.cgi?acc=GSE156614.

[63] Chen L, Luo Z, Zhao C, Li Q et al. AOM/DSS colorectal cancer mouse transcriptome profile across 5 time point in the progression of colites to tumor [RNA-seq]. GSE178145, https://www.ncbi.nlm.nih.gov/geo/query/acc.cgi?acc=GSE178145.

[64] Chen L, Luo Z, Zhao C, Li Q et al. AOM/DSS colorectal cancer mouse transcriptome profile across 5 time point in the progression of colites to tumor [RNA-seq]. GSE178144, https://www.ncbi.nlm.nih.gov/geo/query/acc.cgi?acc=GSE178144.

[65] He X, Ohba S, Hojo H, McMahon AP. AP-1 family members act at DNA targets in conjunction with Sox9 to promote chondrocyte hypertrophy. GSE73372, https://www.ncbi.nlm.nih.gov/geo/query/acc.cgi?acc=GSE73372. 10.1242/dev.134502 PMC5004882 27471255

[66] Wang CY, Cai Z, Chen JD, Lin X, et al. CRC_h3k4m3Enhancer. GitHub. 2023. https://github.com/Wangchenyu5005/crc_h3k4m3Enhancer_1.git.

[67] Lin X, Wang CY. Cooperation of MLL1 and Jun in controlling H3K4me3 on enhancers. 2023. Zenodo. 10.5281/zenodo.10117358. 10.1186/s13059-023-03108-3 PMC10680327 38012744

[68] Lin X. Microscopy images used for quantification in Figure 7.rar. Figshare. 10.6084/m9.figshare.24523762.v1 (2023).

