# Supplementary material for: Cooperation of MLL1 and Jun in controlling H3K4me3 on enhancers in colorectal cancer
Source: Genome Biol. 2023 Nov 27;24:268. doi: 10.1186/s13059-023-03108-3 (PMC10680327; doi:10.1186/s13059-023-03108-3)
Supplement: Supplementary file 1 — Additional file 1: Supplementary Figure S1-S12. Fig. S1. Quality control of sequencing data. Fig. S2. Functional analysis of gain and lost Vm3Es in CRC. Fig. S3. Gene browser view of representative gain Vm3Es in CRC. Fig. S4. Validation of identified Vm3Es. Fig. S5. Inhibition of m3Es represses target gene expression. Fig. S6. Functions of the identified Vm3Es on cell proliferation and cell cycle. Fig. S7. Screen of methyltransferases regulating m3E target genes. Fig. S8. OICR-9429 treatment repressed the identified m3Es. Fig. S9. C646 treatment repressed histone modifications on the identified Vm3Es. Fig. S10. JUN regulates the identified Vm3Es in CRC. Fig. S11. JUN binds to H3K4me3 enhancer loci in the AOM-DSS induced CRC model. Fig. S12. OICR-9429 treatment repressed CRC. [file 13059_2023_3108_MOESM1_ESM.pdf]

Fig. S1

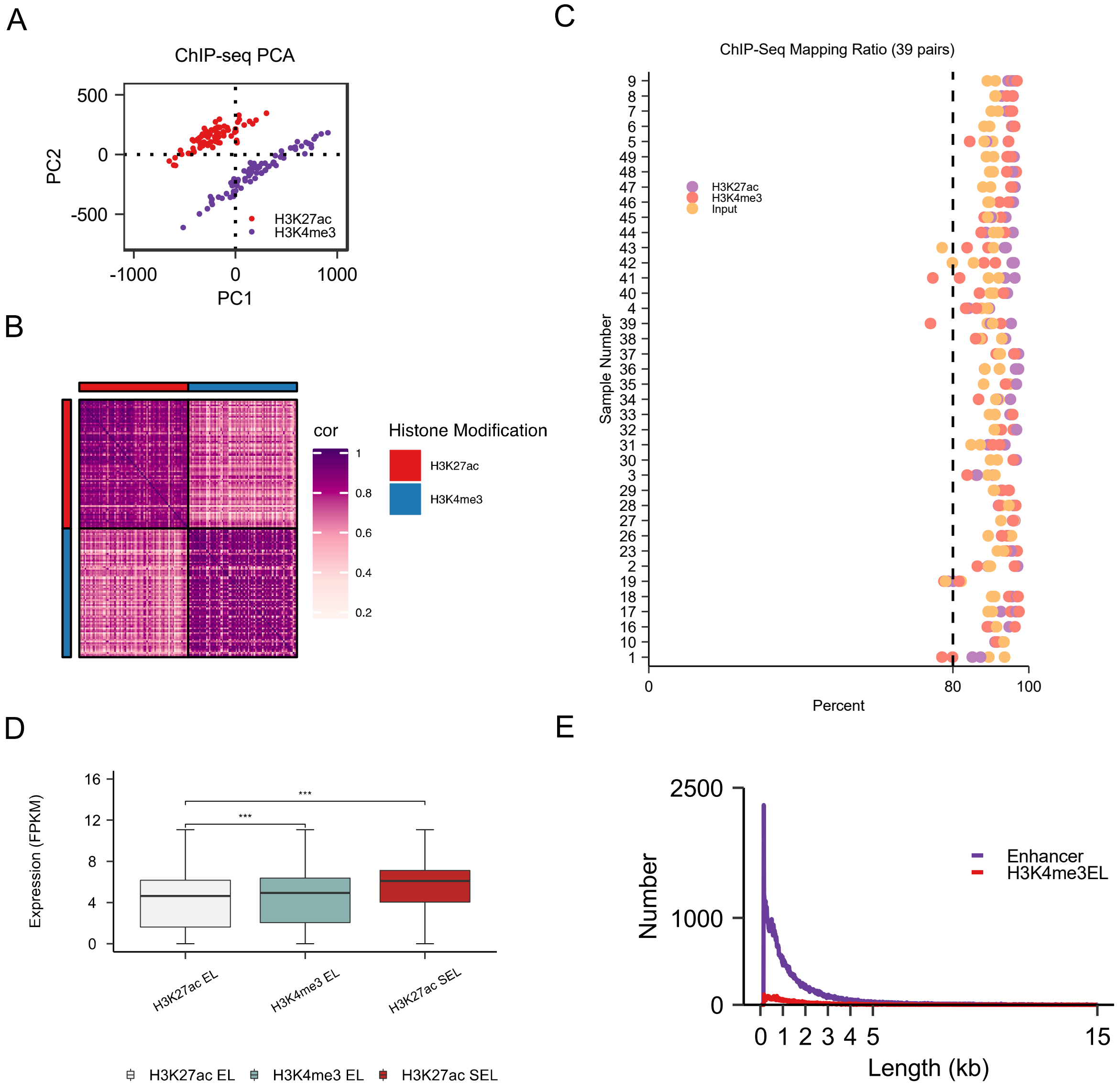

Fig. S1 Quality control of sequencing data. (A) PCA using H3K27ac and H3K4me3 on whole genome. **(B)** Heatmap shows the correlation between samples with different modifications. **(C)** Mapping ratio of ChIP-Seq 39 pairs. H3K27ac: purple, H3K4me3: orange, Input: yellow. **(D)** Gene expression associated with typical enhancers (white) , m3Es (green) and super enhancers (red). Statistical analysis was performed using a two-sided Student t test. p value was labelled on the corresponding items. ***p < 0.001. **(E)** The length distribution of typical enhancer (purple) and m3Es (red).

Fig. S2

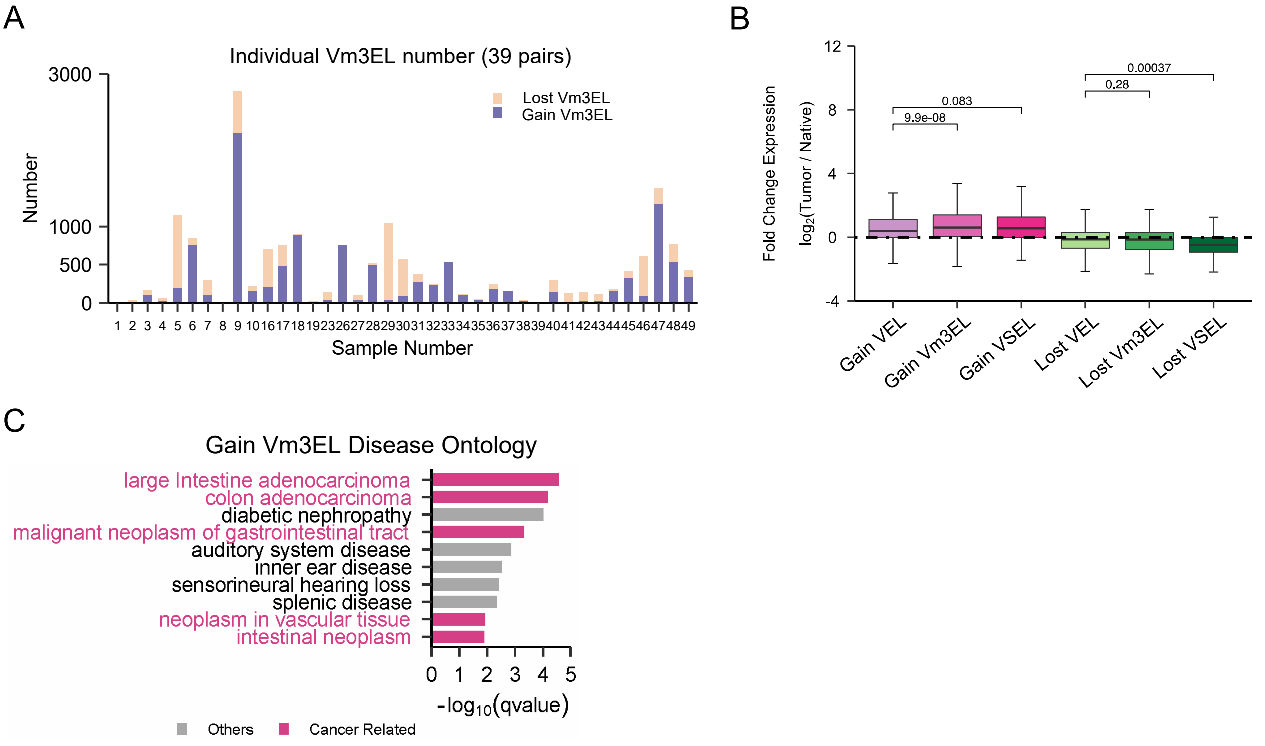

Fig. S2 Functional analysis of gain and lost Vm3Es in CRC. (A) Vm3Es (including gain and lost Vm3Es) number of each pair of samples. **(B)** Fold change of gene expression associated with gained (red) VELs, VSELs and Vm3Es (red), and lost (green) VELs, VSELs and Vm3Es. Statistical analysis was performed using a two-sided Student t test. p value was labelled on the corresponding items. **(C)** The human disease ontology which gained Vm3Es participated in enriched by GREAT (version 3.0.0). The red bar represents cancer related terms and the grey bar represents other terms.

Fig. S3

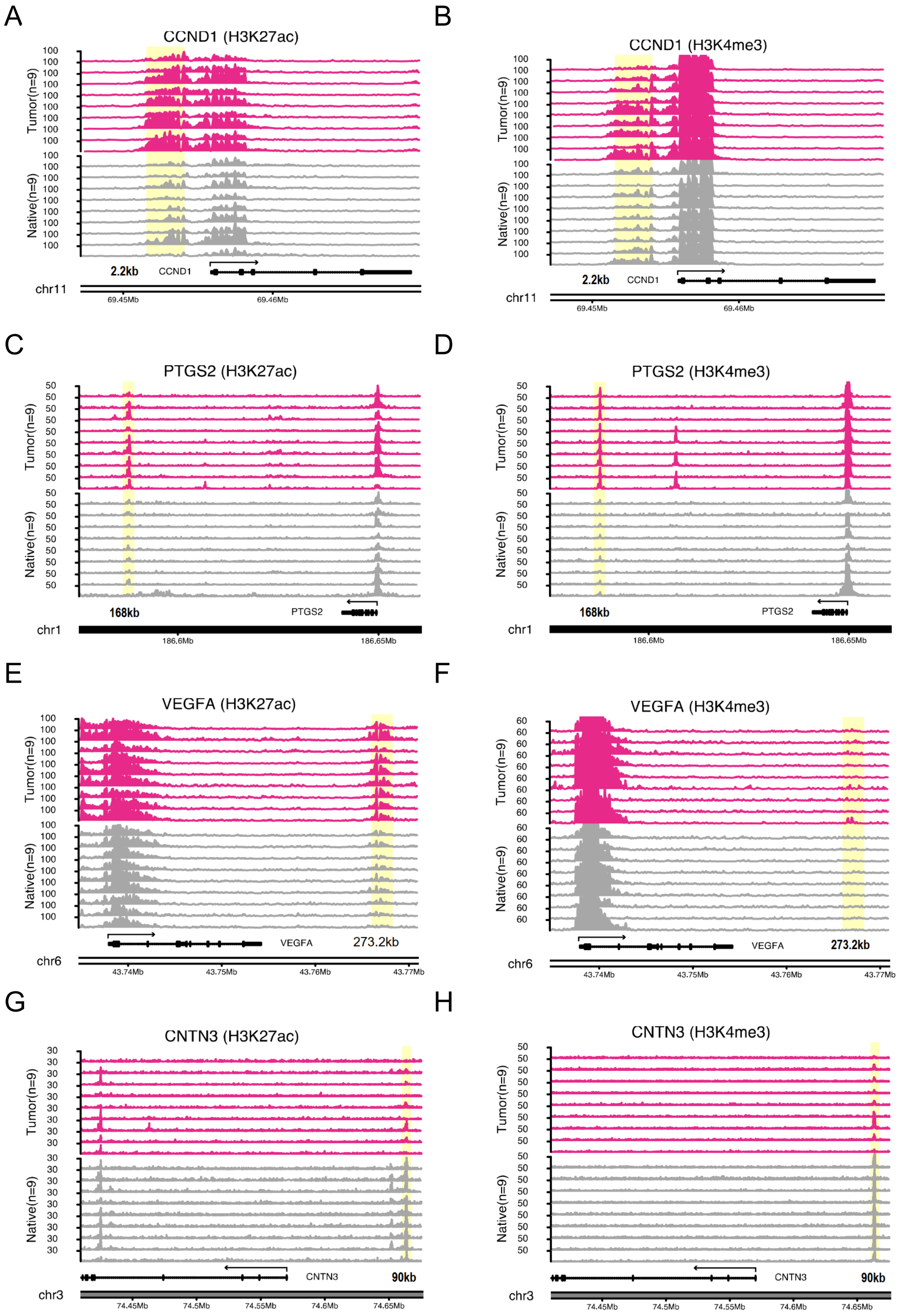

Fig. S3 Gene browser view of representative gain Vm3Es in CRC. (A&B) Representative H3K27ac (A) and H3K4me3 (B) tracks of gain Vm3E at *CCND1* gene loci. **(C&D)** Representative H3K27ac (C) and H3K4me3 (D) tracks of gain Vm3E at *PTSG2* gene loci. **(E&F)** Representative H3K27ac (E) and H3K4me3 (F) tracks of gain Vm3E at *VEGFA* gene loci. **(G&H)** Representative H3K27ac (E) and H3K4me3 (F) tracks of lost Vm3E at *CNTN3* gene loci. Vm3Es was highlighted in a yellow shade.

Fig. S4

A

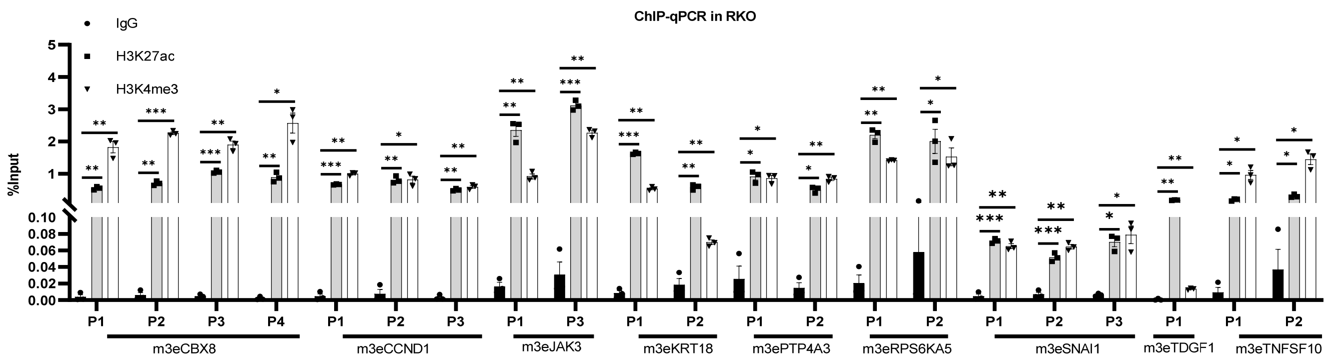

B

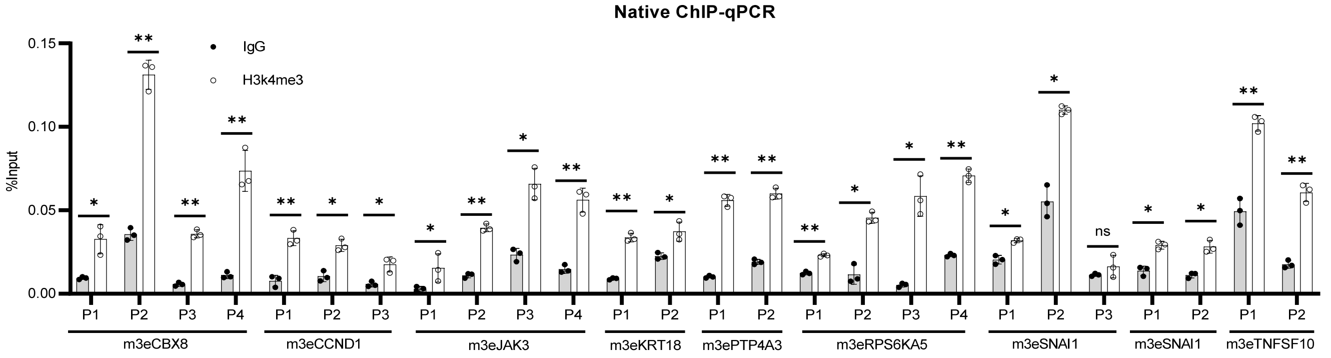

Fig. S4 Validation of identified Vm3Es. (A) ChIP-qPCR showing the H3K27ac and H3K4me3 level at m3Es in HCT116. **(B)** Native ChIP-qPCR showing the H3K4me3 level at m3Es in RKO.

Fig. S5

A

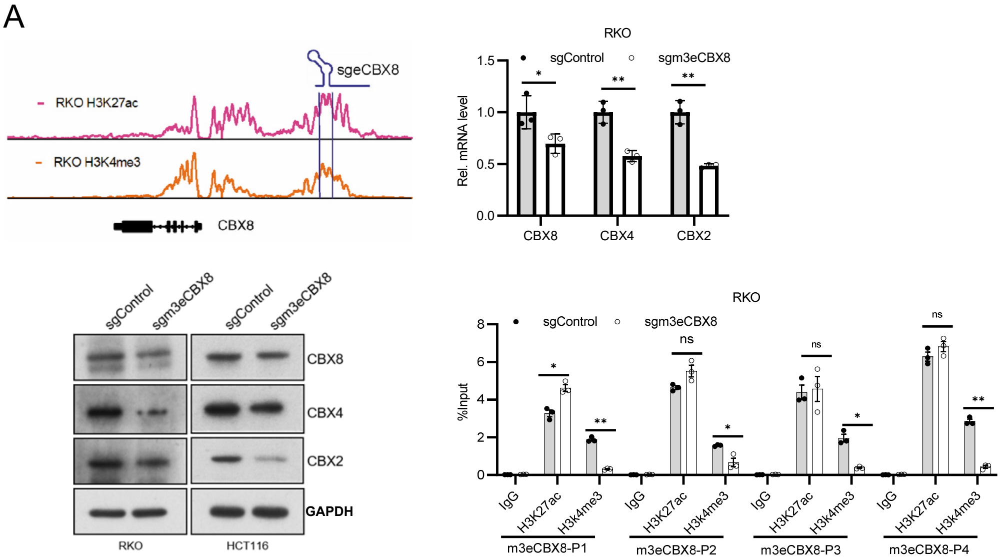

sgeCBX8
RKO H3K27ac
RKO H3K4me3
CBX8
RKO
sgControl
sgm3eCBX8
Rel. mRNA level
CBX8
CBX4
CBX2
sgControl
sgm3eCBX8
sgControl
sgm3eCBX8
CBX8
CBX4
CBX2
GAPDH
RKO
HCT116
RKO
%Input
IgG
H3K27ac
H3K4me3
m3eCBX8-P1
m3eCBX8-P2
m3eCBX8-P3
m3eCBX8-P4
ns

B

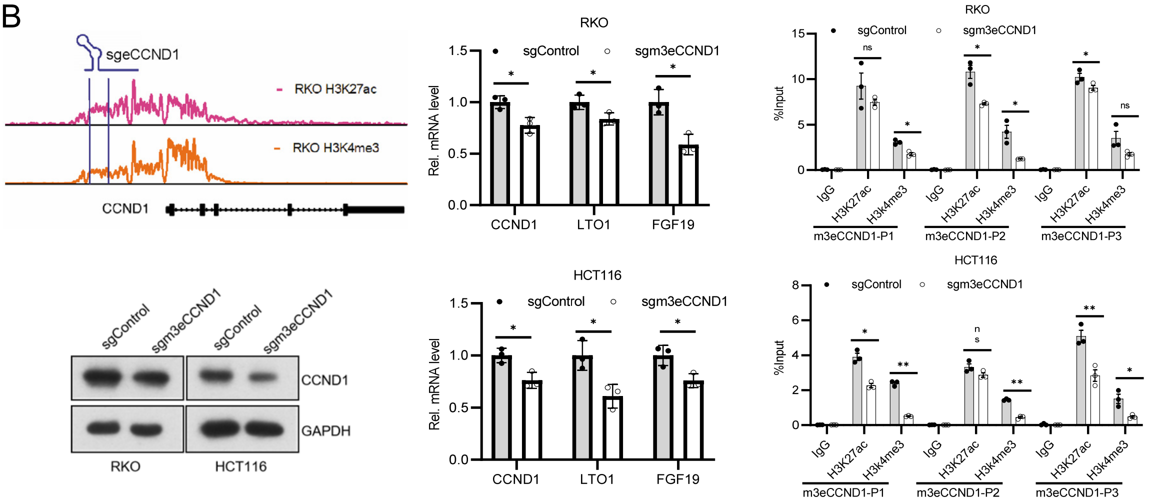

sgeCCND1
RKO H3K27ac
RKO H3K4me3
CCND1
RKO
sgControl
sgm3eCCND1
Rel. mRNA level
CCND1
LTO1
FGF19
%Input
IgG
H3K27ac
H3K4me3
m3eCCND1-P1
m3eCCND1-P2
m3eCCND1-P3
sgControl
sgm3eCCND1
sgControl
sgm3eCCND1
CCND1
GAPDH
RKO
HCT116
HCT116
ns

C

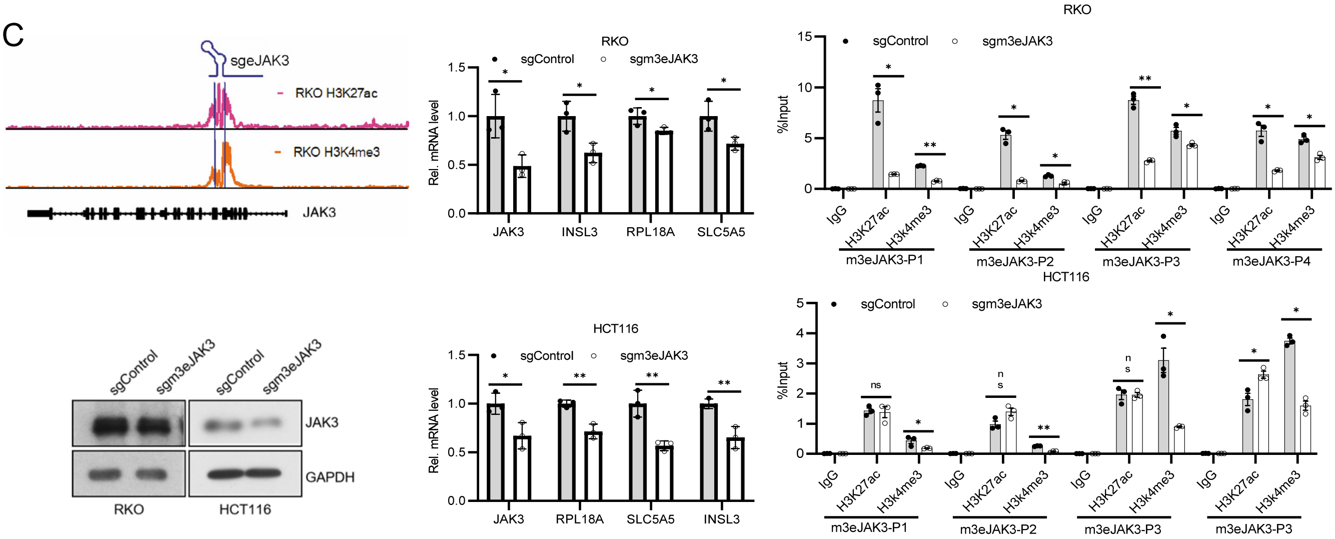

sgeJAK3
RKO H3K27ac
RKO H3K4me3
JAK3
RKO
sgControl
sgm3eJAK3
Rel. mRNA level
JAK3
INSL3
RPL18A
SLC5A5
%Input
IgG
H3K27ac
H3K4me3
m3eJAK3-P1
m3eJAK3-P2
m3eJAK3-P3
m3eJAK3-P4
HCT116
sgControl
sgm3eJAK3
sgControl
sgm3eJAK3
JAK3
GAPDH
RKO
HCT116
ns

Fig. S5 (Continued)

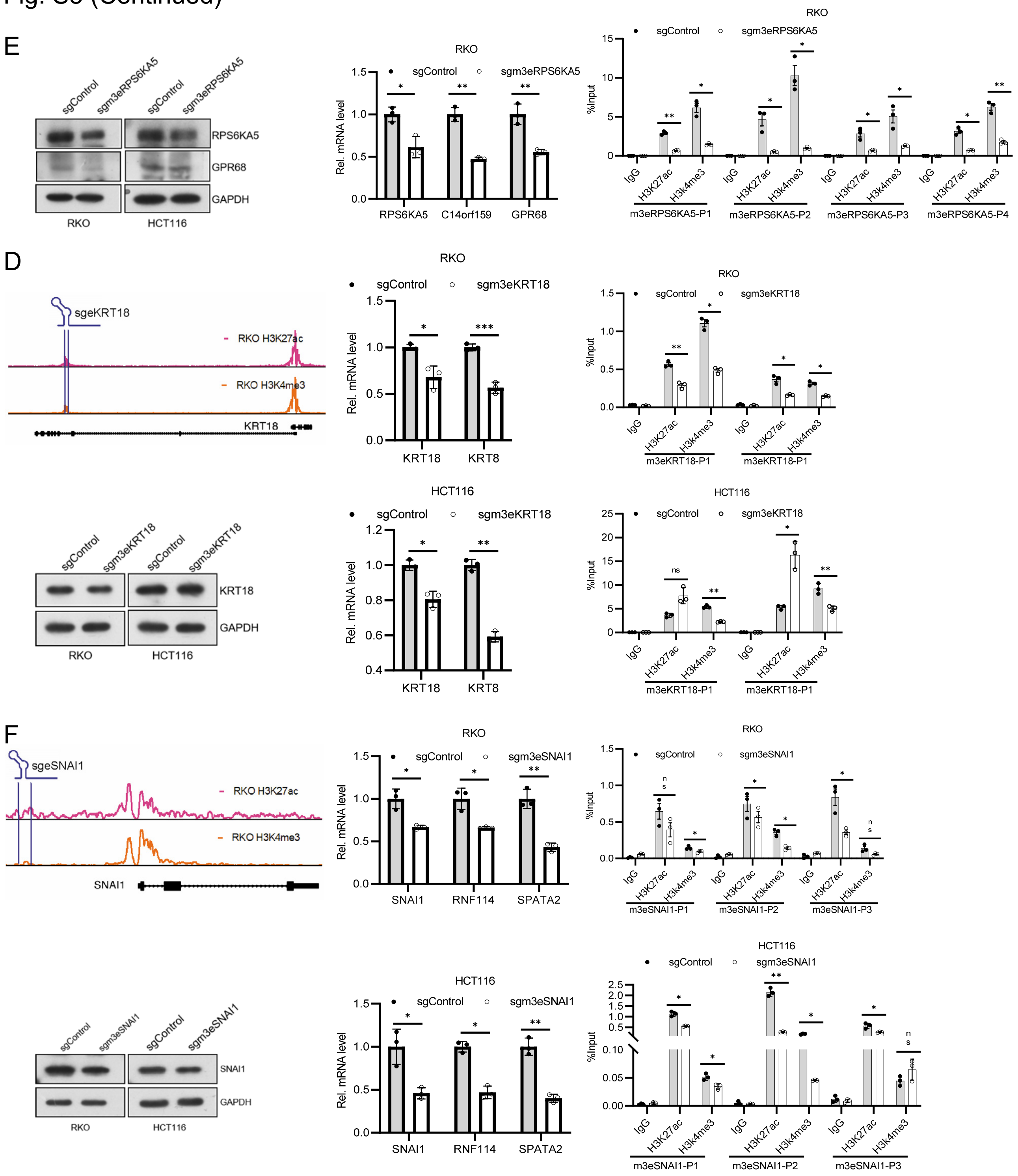

Fig. S5 (Continued)

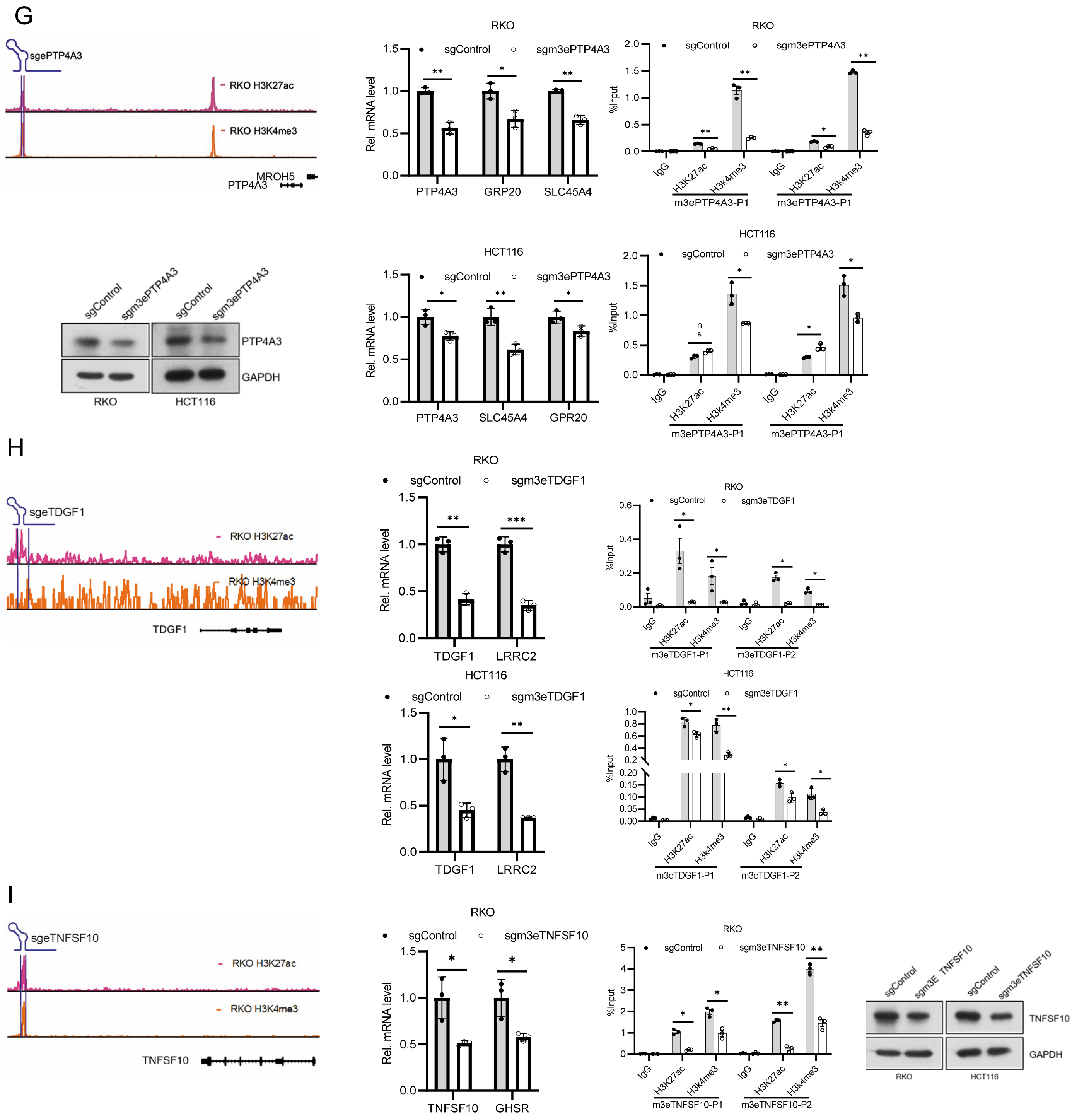

Fig. S5 Inhibition of m3Es represses target gene expression. The sketch maps of sgRNA design, target gene expression of mRNA and protein, histone modifications on the m3Es proximal to *CBX8* (A), *CCND1* (B), *JAK3* (C), *KRT18* (D), *RPS6KA5* (E), *SNAI1* (F), *PTP4A3* (F), *TDGF1* (H) and *TNFSF10* (I), in RKO and HCT116 cells . The indicated cells were transfected with multiple sgRNAs together and the antibiotic-selected pooled stable cells were used for study. * means p value < 0.05, ** for p value < 0.01.Data are represented as mean ± SD or SEM. *p*-values are calculated by two-tailed unpaired t test.

Fig. S6

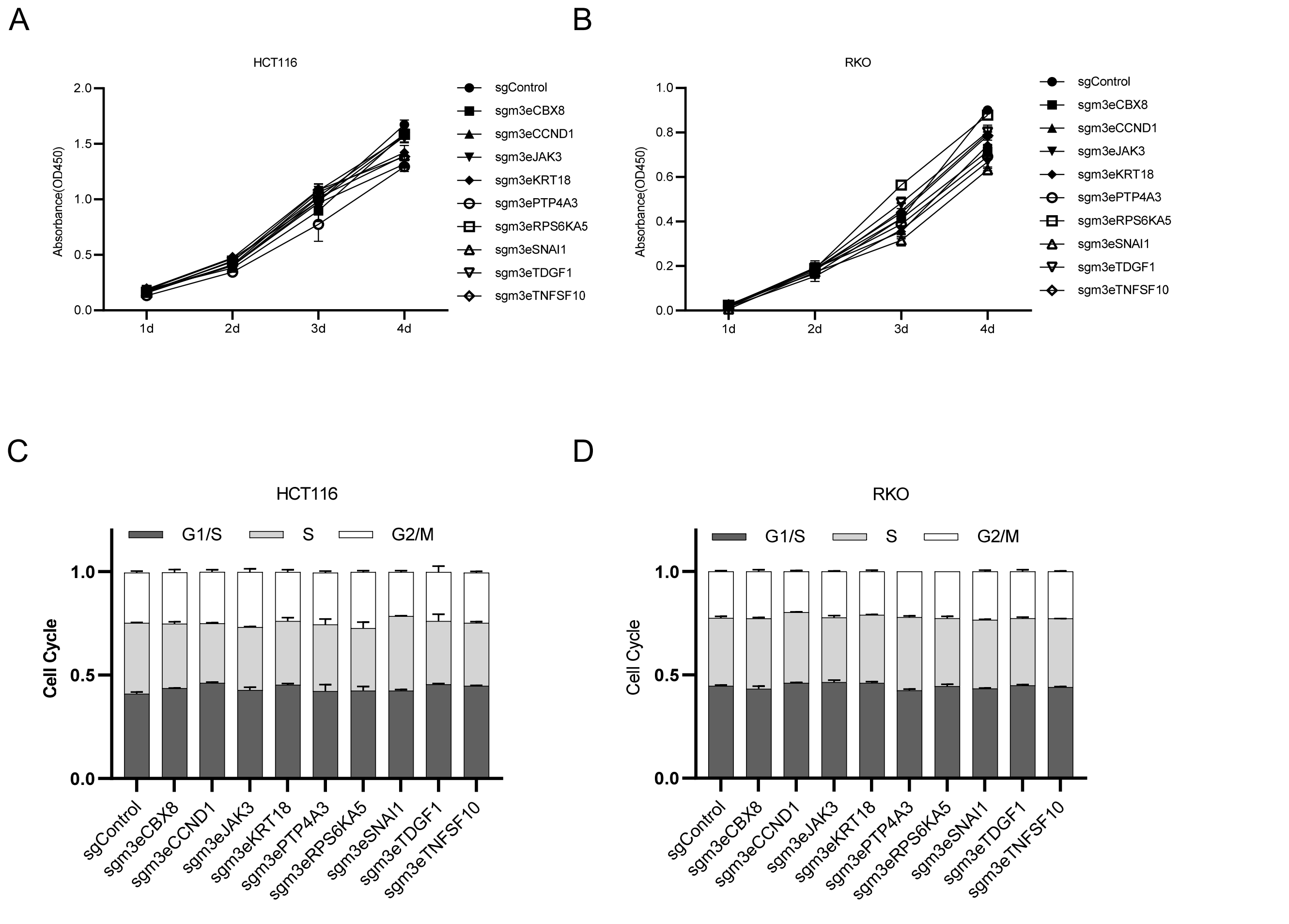

Fig. S6 Functions of the identified Vm3Es on cell proliferation and cell cycle. (A&B) Cell proliferation analyzed by CCK-8 assay of the above cell lines. **(C&D)** Cell cycle distributions analyzed by flowcytometry of stable cell lines with stable expression of the indicated CRISPR/sgRNAs.

Fig. S7

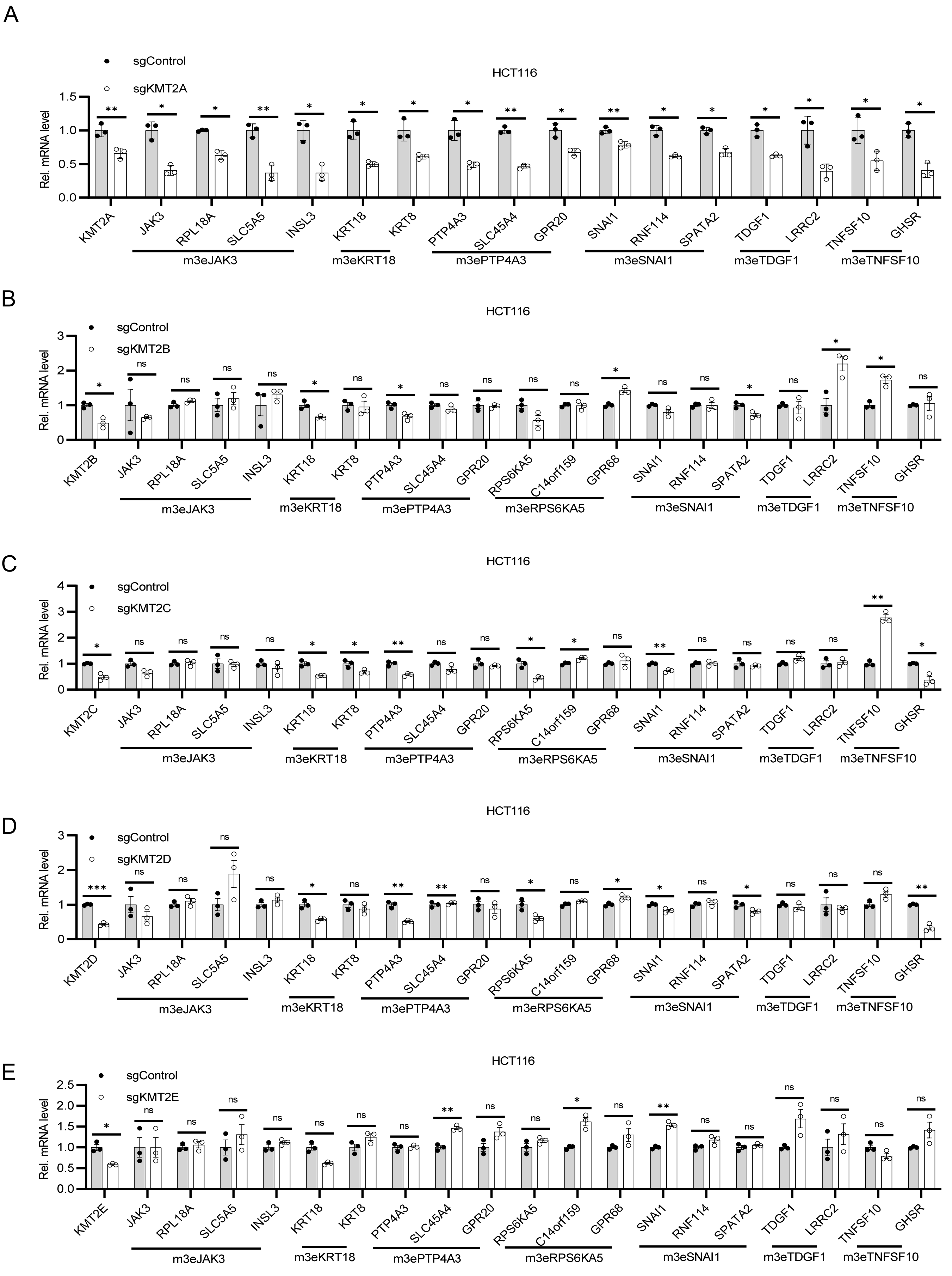
A
HCT116
sgControl
sgKMT2A
Rel. mRNA level
KMT2A
JAK3
RPL18A
SLC5A5
INSL3
KRT18
KRT8
PTP4A3
SLC45A4
GPR20
SNAI1
RNF114
SPATA2
TDGF1
LRRC2
TNFSF10
GHSR
m3eJAK3
m3eKRT18
m3ePTP4A3
m3eSNAI1
m3eTDGF1
m3eTNFSF10
B
HCT116
sgControl
sgKMT2B
Rel. mRNA level
KMT2B
RPS6KA5
C14orf159
GPR68
m3eRPS6KA5
C
HCT116
sgControl
sgKMT2C
Rel. mRNA level
KMT2C
D
HCT116
sgControl
sgKMT2D
Rel. mRNA level
KMT2D
E
HCT116
sgControl
sgKMT2E
Rel. mRNA level
KMT2E

Fig. S7 (continued)

F

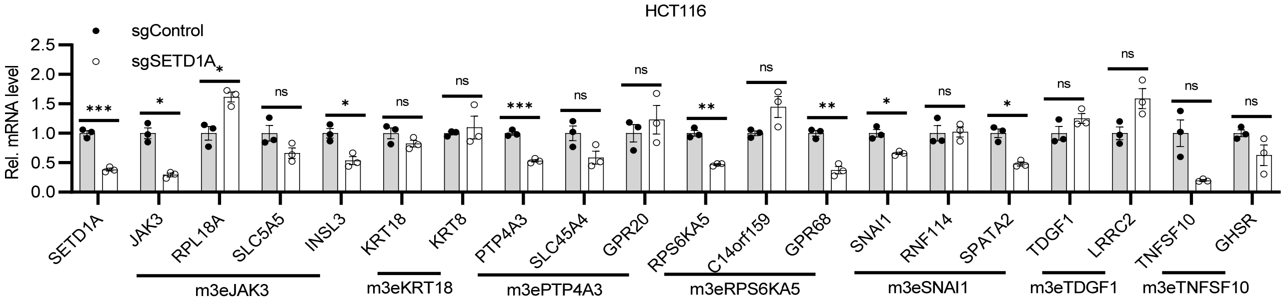

G

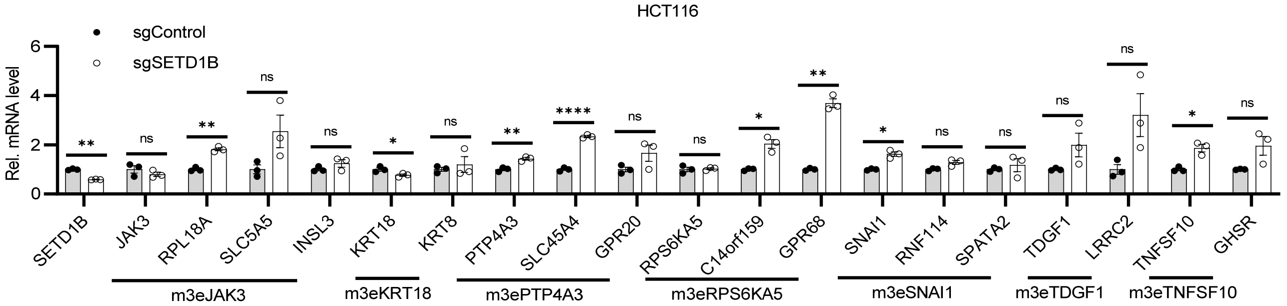

H

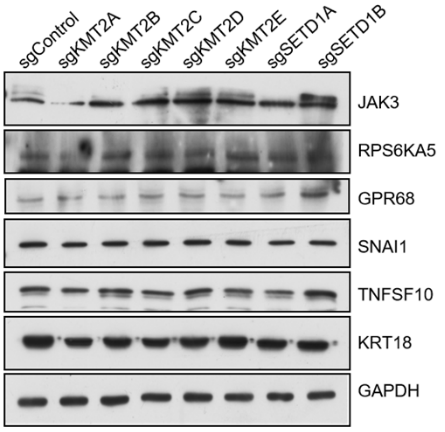

Fig. S7 Screen of methyltransferases regulating m3E target genes. Bar plot showing the relative mRNA level of enhancer loci adjacent genes in *KMT2A* (A), *KMT2B* (B), *KMT2C* (C), *KMT2D* (D), *KMT2E* (E), *SETD1A* (F) and *SETD1B* (G) KD HCT116 cell line. **(H)** Western blotting of the proximal genes for m3Es in *KMT2A-E*, *SETD1A&B* KD HCT116 cells.

Fig. S8

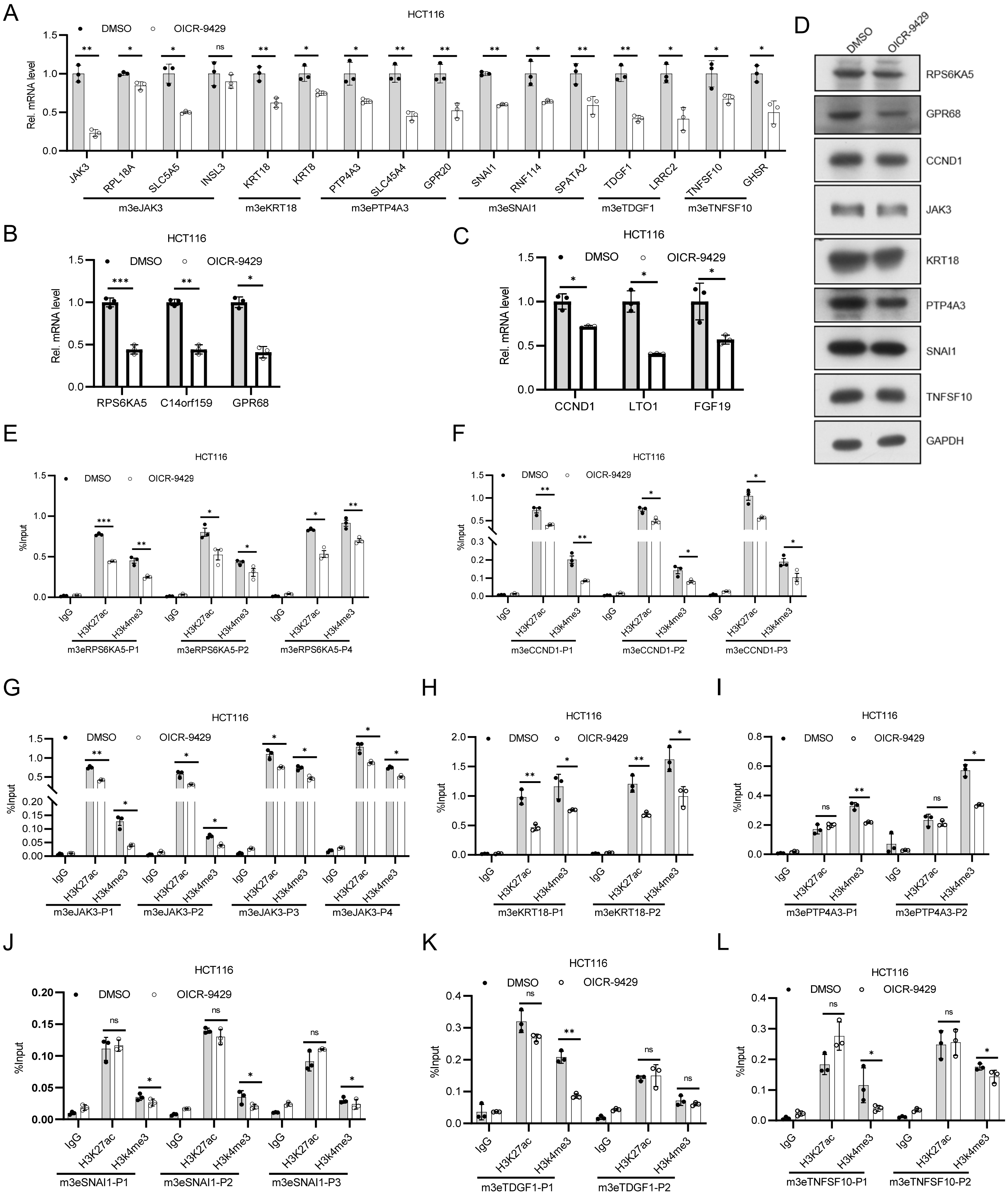

Fig. S8 OICR-9429 treatment repressed the identified m3Es. (A-C) Bar plot showing the relative mRNA level of the proximal genes for m3Es in HCT116 after OICR-9429 (10 μM) treatment for 48h. **(D)** Western blotting of the proximal genes for m3Es in HCT116 after OICR-9429 (10 μM) treatment for 48h. **(E-L)** ChIP-qPCR assessing H3K27ac and H3K4me3 level on m3Es of *RPS6KA5* (E), *CCND1* (F), *JAK3* (G), *KRT18* (H), *PTP4A3* (**I**), *SNAI1* (J), *TDGF1* (K) and *TNFSF10* (L) in HCT116 with OICR-9429 treatment (10 μM) for 48h.

Fig. S9

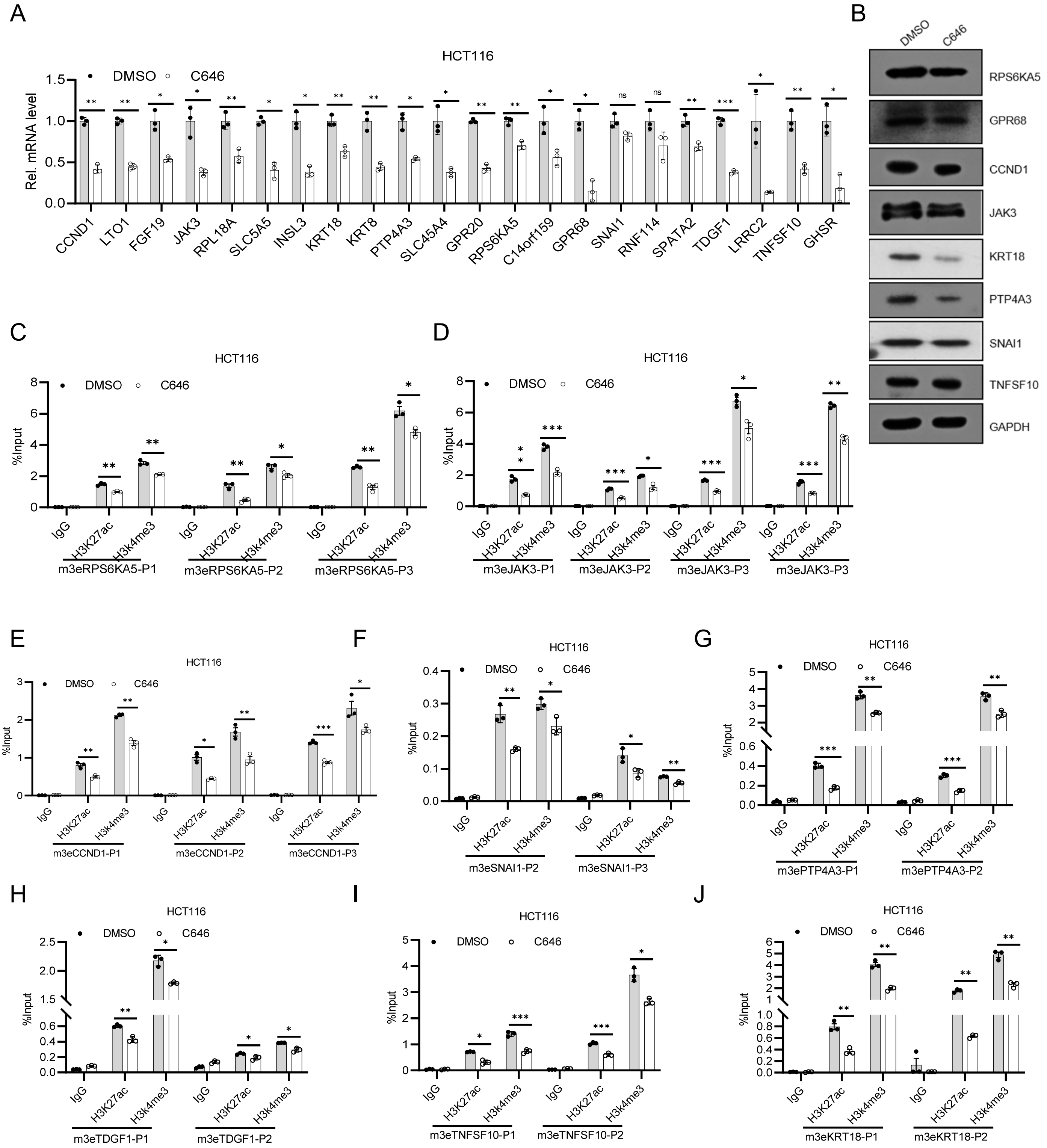

Fig. S9 C646 treatment repressed histone modifications on the identified Vm3Es. (A) Bar plot showing the relative mRNA level of the proximal genes for m3Es in HCT116 after C646 treatment (10 μM) for 12h. **(B)** Western blotting of the proximal genes for m3Es in HCT116 after OICR-9429 (10 μM) treatment for 48h. **(C-J)** ChIP-qPCR assessing H3K27ac and H3K4me3 level on the m3Es of *RPS6KA5* (C), *JAK3* (D), *CCND1* (E), *SNAI1* (F), *PTP4A3* (G), *TDGF1* (H), *TNFSF10* (I) and *KRT18* (J) in HCT116 with the treatment of C646 (10 μM) for 12h.

Fig. S10

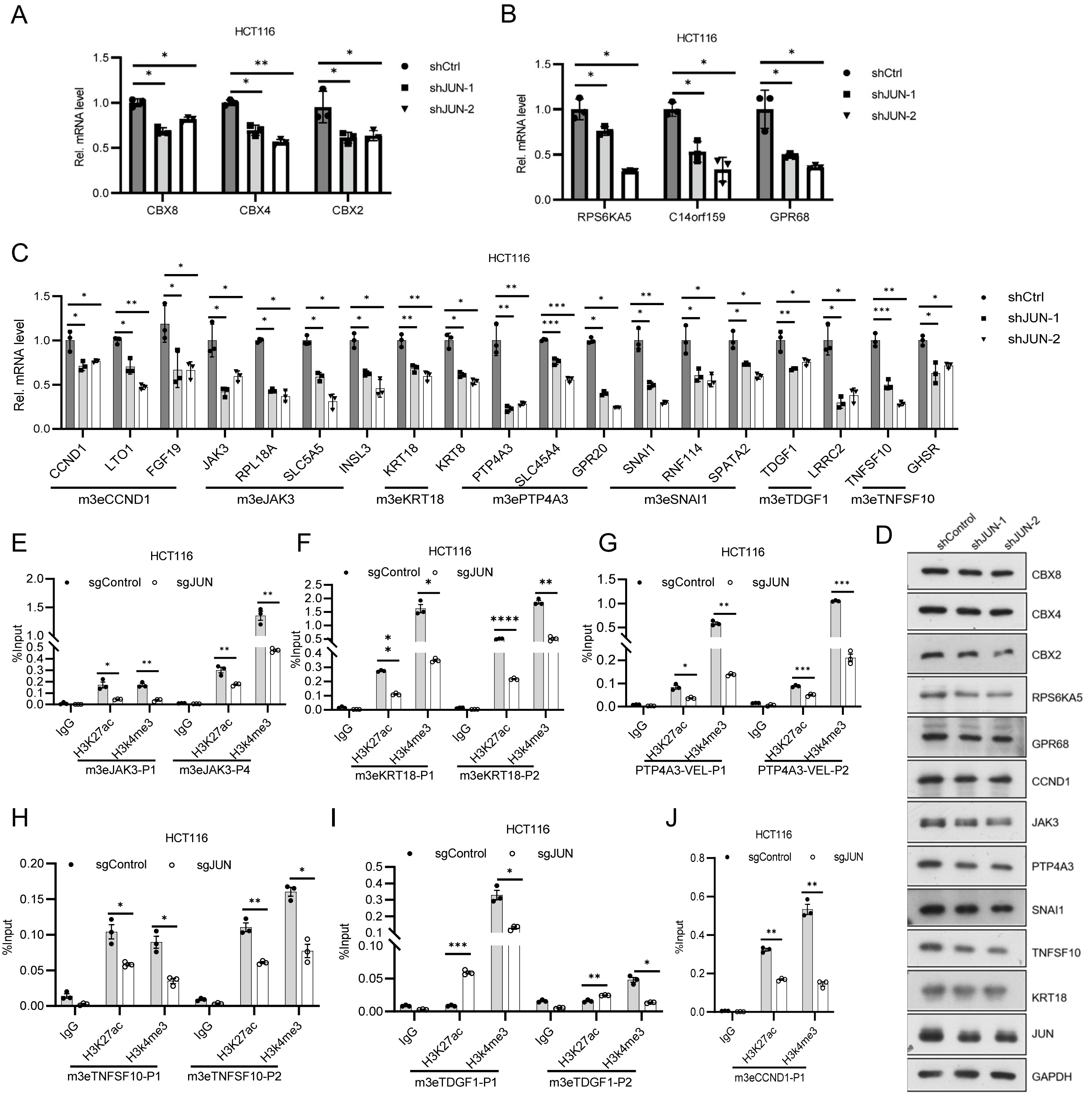

Fig. S10 JUN regulates the identified Vm3Es in CRC. (A-C) Bar plot showing the relative mRNA level of the proximal genes for m3Es in JUN KD HCT116 cells. **(D)** Western blotting of the proximal genes for for m3Es in JUN KD HCT116 cells. **(E-J)** ChIP-qPCR assessing H3K27ac and H3K4me3 level on the m3Es of *JAK3* (E), *KRT18* (F), *PTP4A3* (G), *TNFSF10* (H), *TDGF1* (I) and *CCND1* (J) in JUN KD cell line.

Fig. S11

A

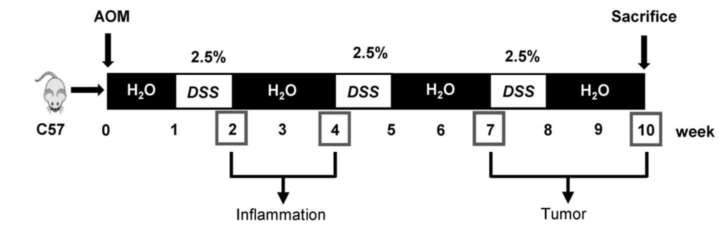

C

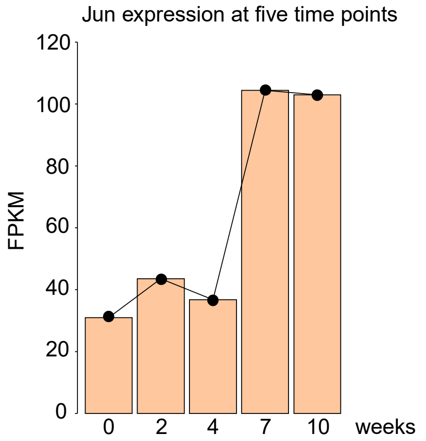

B

The number of enhancers

Time point	H3K27ac enhancer	H3K4me3 enhancer
Ctrl	13218	3519
2weeks	7902	2228
4weeks	13161	2434
7weeks	12258	1879
10weeks	24163	1692

D

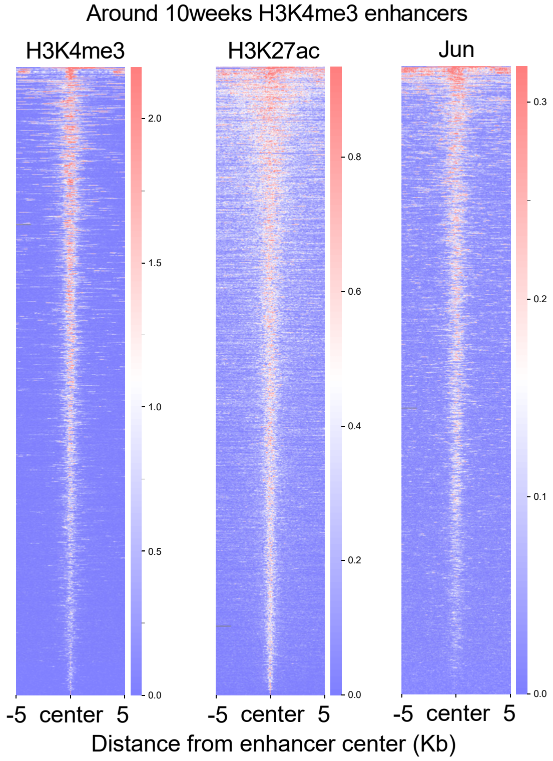

E

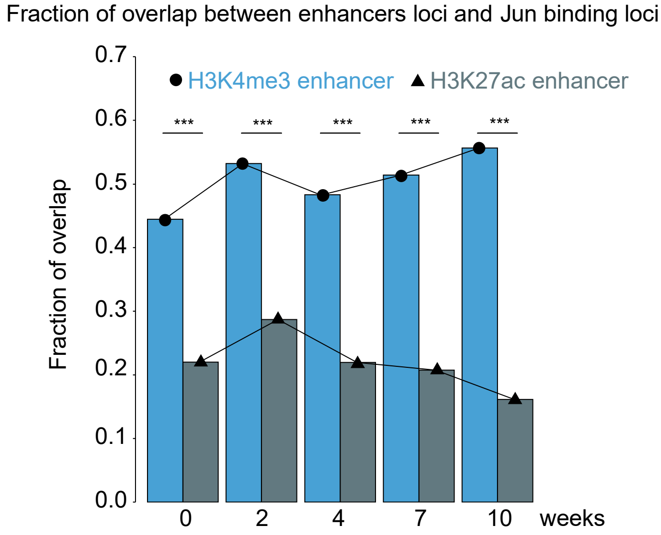

Fig. S11 JUN binds to H3K4me3 enhancer loci in the AOM-DSS induced CRC model. (A) Experimental workflow for studying the H3K4me3 enhancer of Colitis-Associated Cancer. 2- and 4-week represented the inflammation stage and 7- and 10-week for tumor stage. **(B)** The number of H3K27ac enhancers and H3K4me3 enhancers at five time points. **(C)** Jun expression at five time points. FPKM: Fragments Per Kilobase of transcript per Million fragments mapped. **(D)** Heatmaps of H3K4me3, H3K27ac and JUN signals (CPM) over 10weeks H3K4me3 enhancer loci. All rows are centered on the peaks center ± 5 kb. CPM: Counts Per Million. **(E)** JUN are more likely to show evidence of binding to H3K4me3 enhancer loci. χ^2 test of independence between JUN binding loci and two types of enhancer loci.

Fig. S12

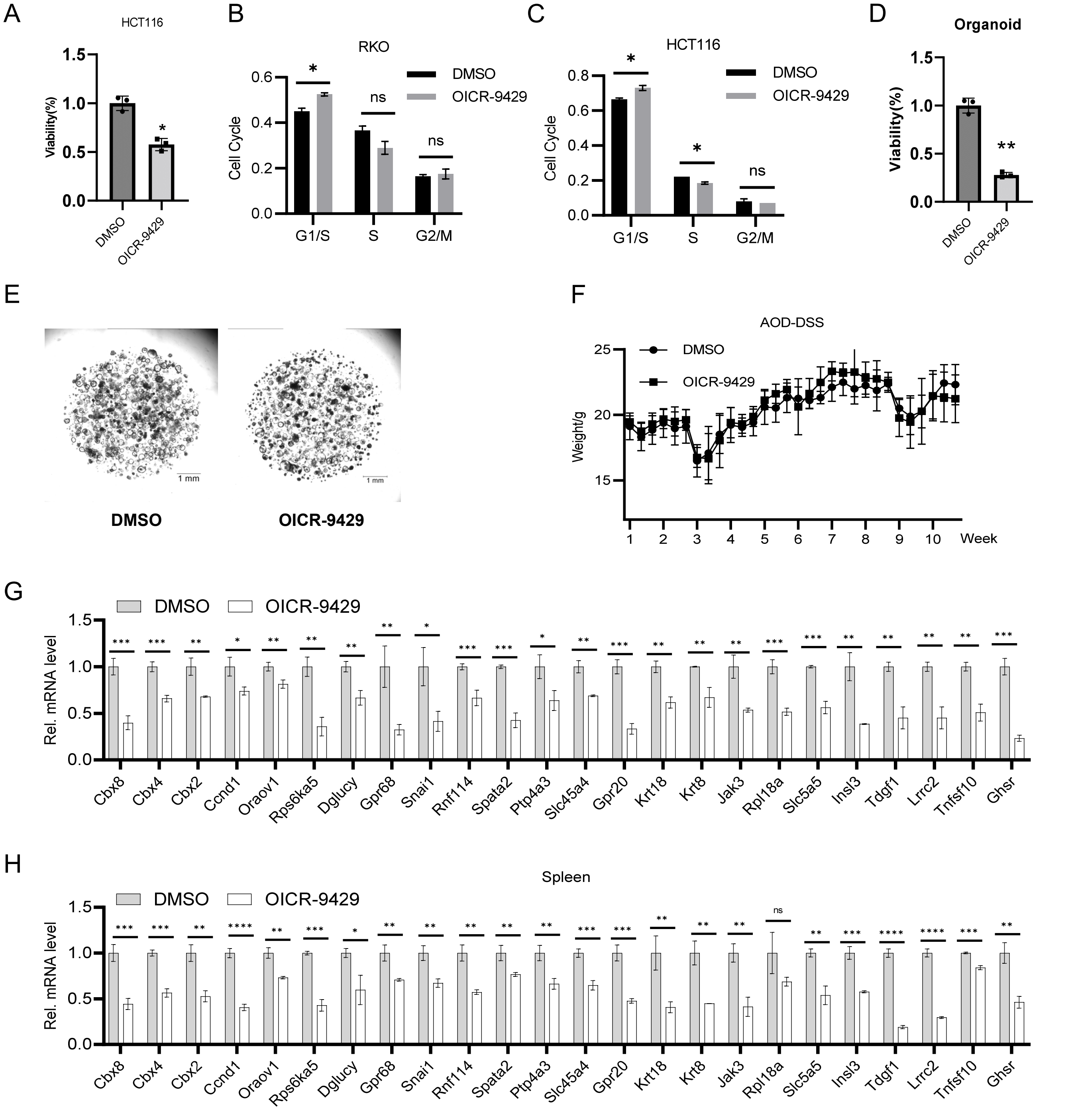

Fig. S12 OICR-9429 treatment repressed CRC. (A) Cell viability analyzed by CCK-8 assay of HCT116 with the treatment of OICR-9429 (80 μM) for 48h. **(B-C)** Cell cycle distributions analyzed by flowcytometry of HCT116(B) and RKO(C) with the treatment of OICR-9429(20uM) for 48h. **(D)** Viability of colorectal cancer organoid with the treatment of OICR-9429 (40 μM) for 7 days, measured by CCK-8 assay. **(E)** Colorectal cancer organoid morphology imaged by a light microscope after the treatment of OICR-9429 (40 μM) for 7 days. **(F)** Mean body weight increment of the mice subject to the AOM/DSS treatment during the experimental procedure. **(G&H)** Bar plot showing the relative mRNA level of the proximal genes for m3Es in intestine and spleen tissue of AOM-DSS induced CRC model with intraperitoneally injected with OICR-9429 (5 mg/kg, three time per week at the indicated time points).
