# Supplementary material for: Cooperation of MLL1 and Jun in controlling H3K4me3 on enhancers in colorectal cancer
Source: Genome Biol. 2023 Nov 27;24:268. doi: 10.1186/s13059-023-03108-3 (PMC10680327; doi:10.1186/s13059-023-03108-3)

Uncropped western blotting analysis

Uncropped western blotting and gel for Fig. 5

Fig. 5D

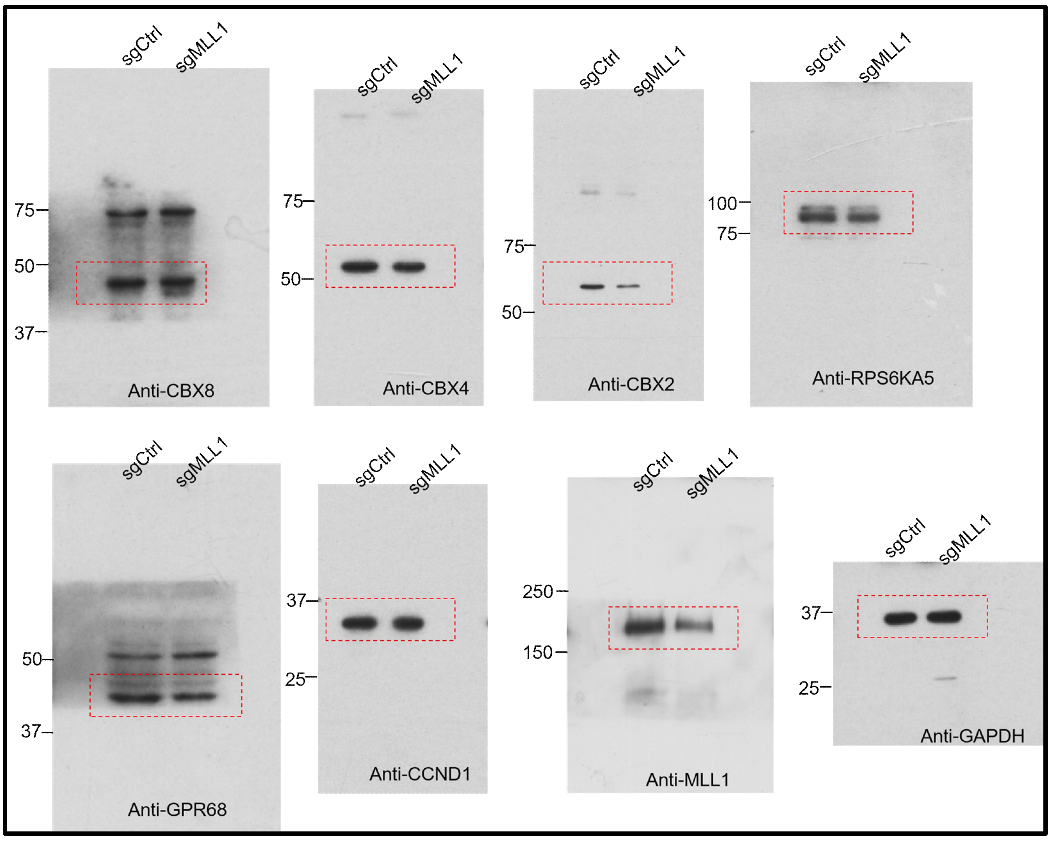

Fig. 5J

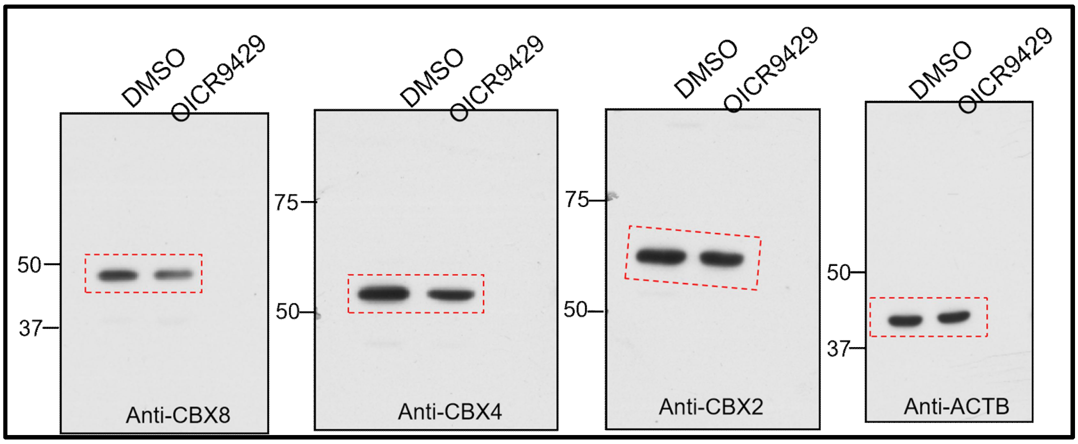

Fig. 5M

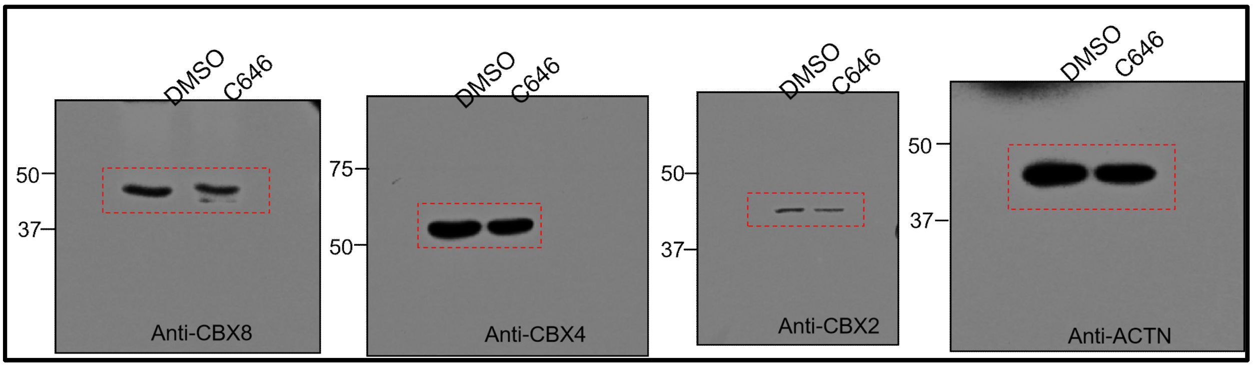

Uncropped western blotting and gel for Fig. 6

Fig. 6D

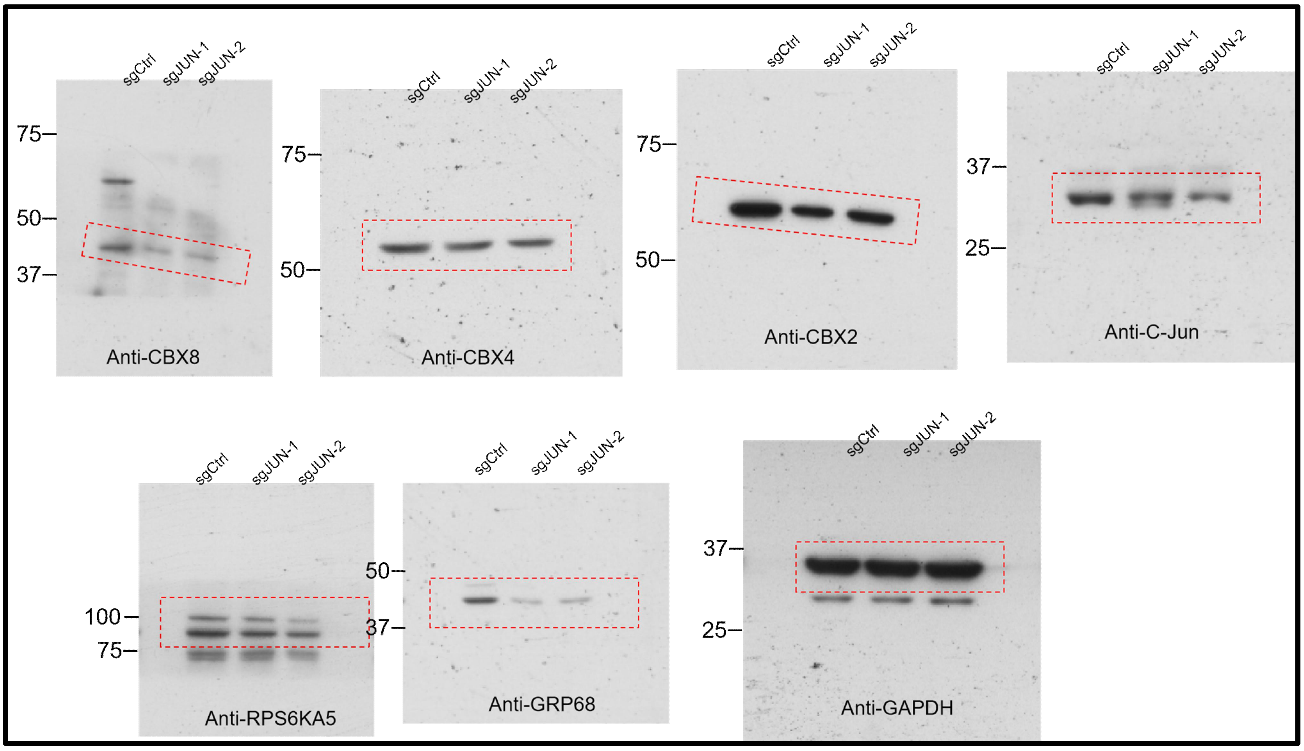

Fig. 6G

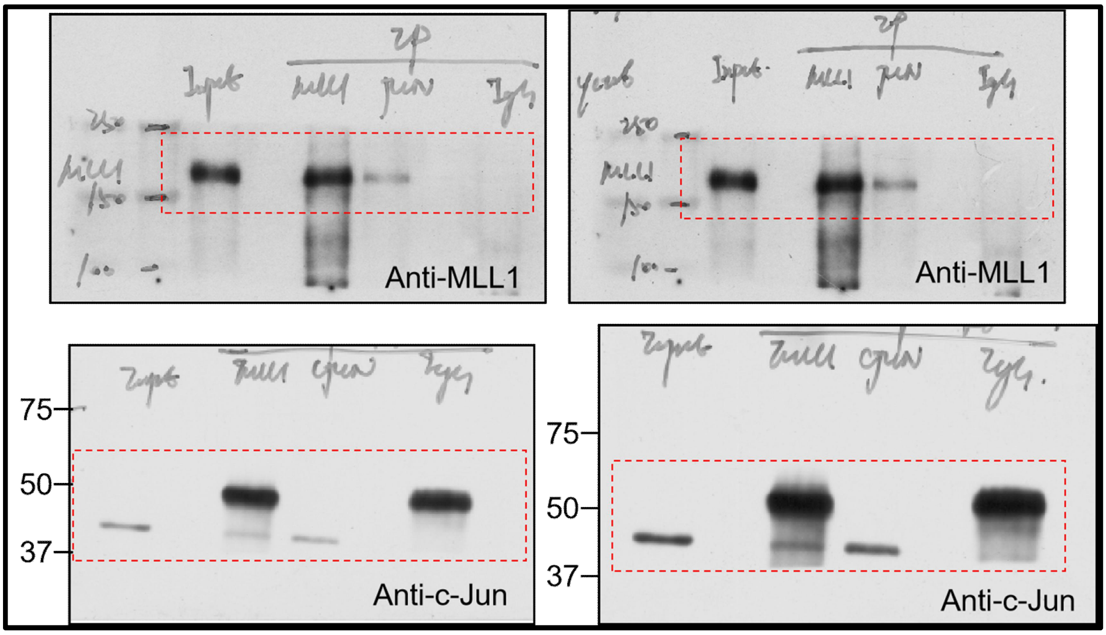

Uncropped western blotting and gel for Fig. S5

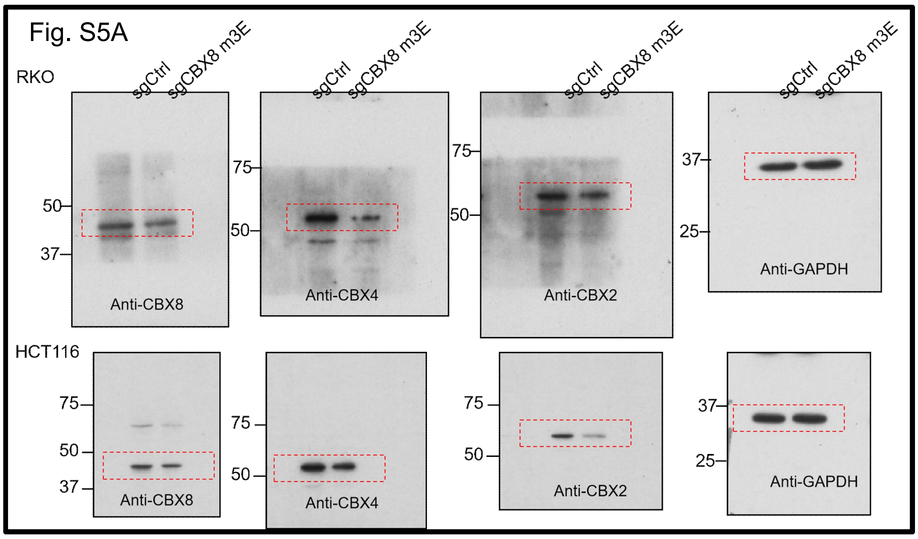

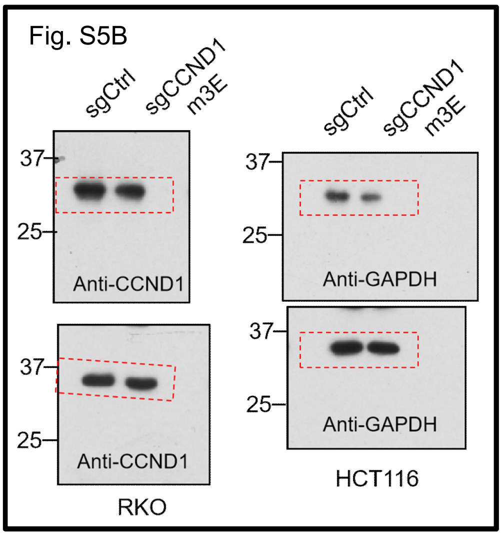

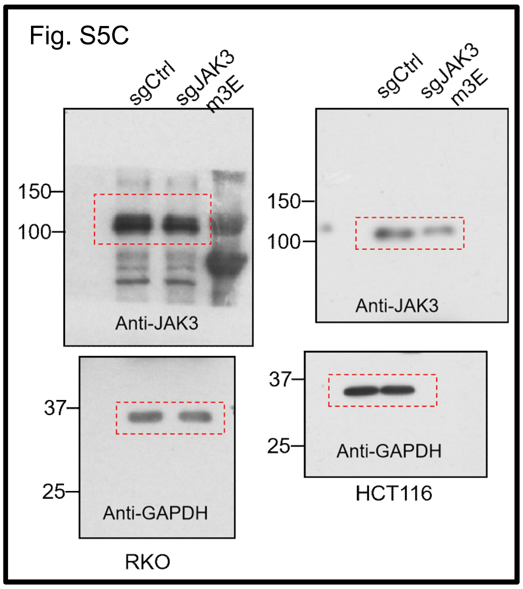

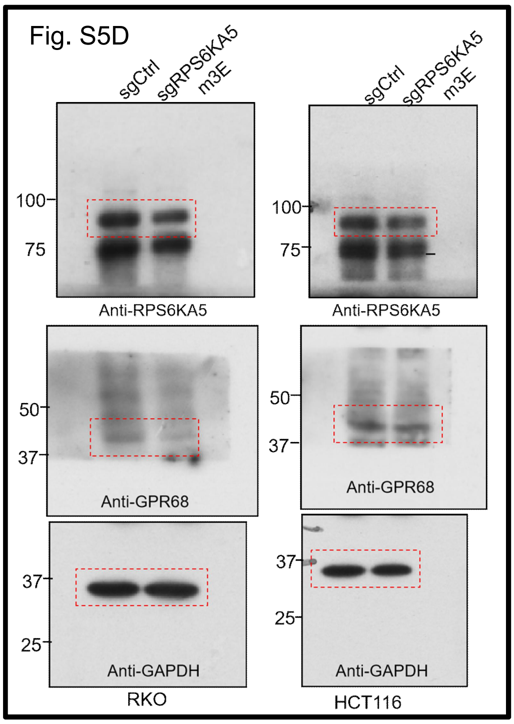

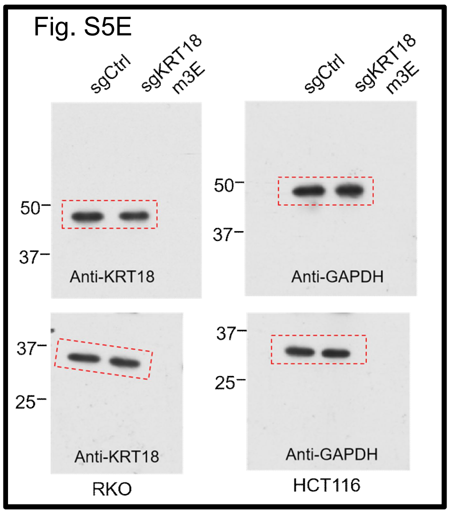

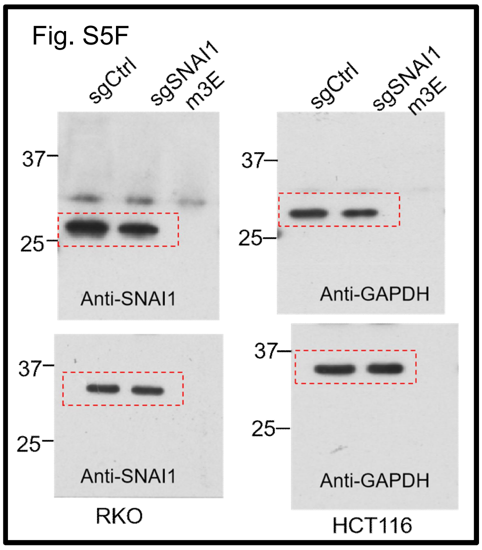

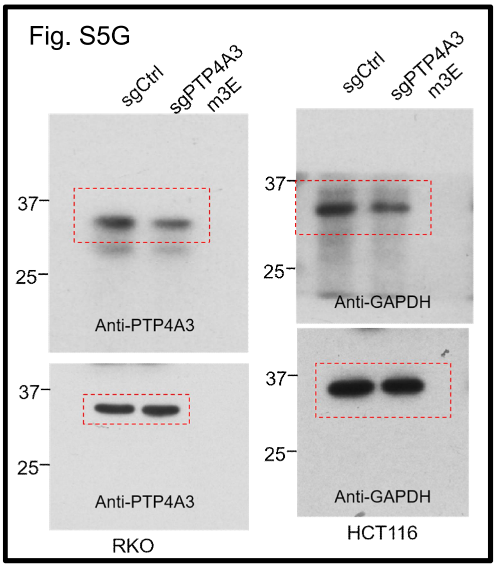

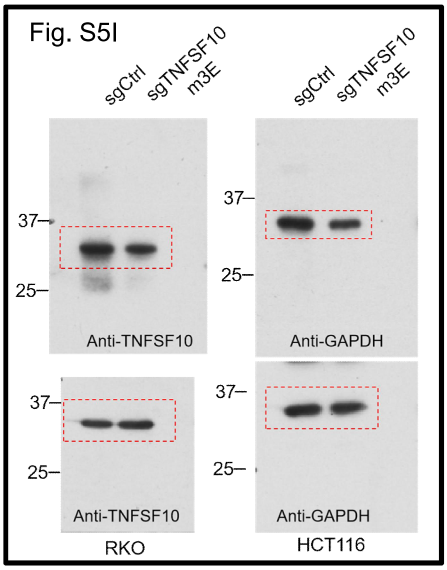

Uncropped western blotting and gel for Fig. S7

Fig. S7H

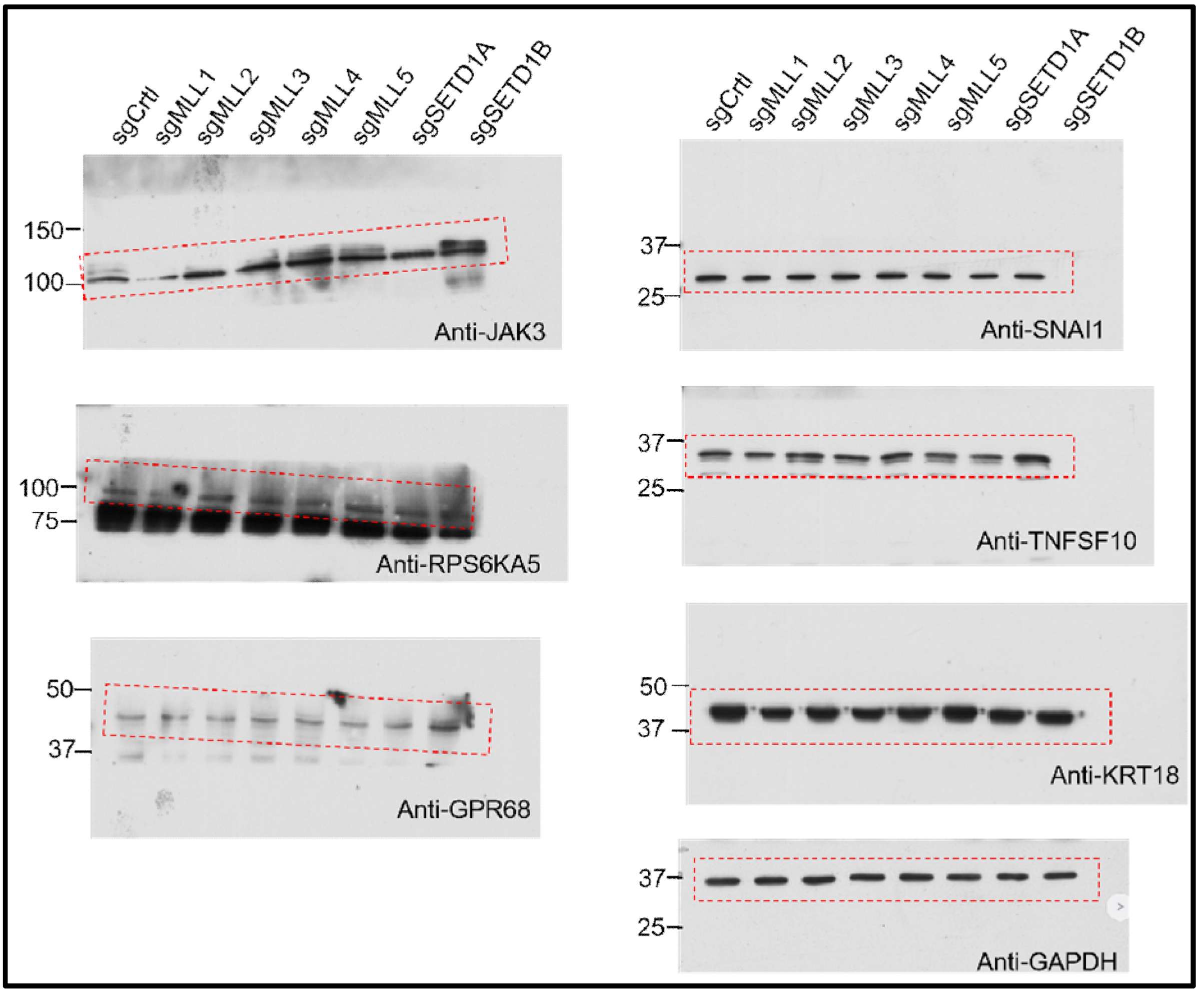

Uncropped western blotting and gel for Fig. S8

Fig. S8D

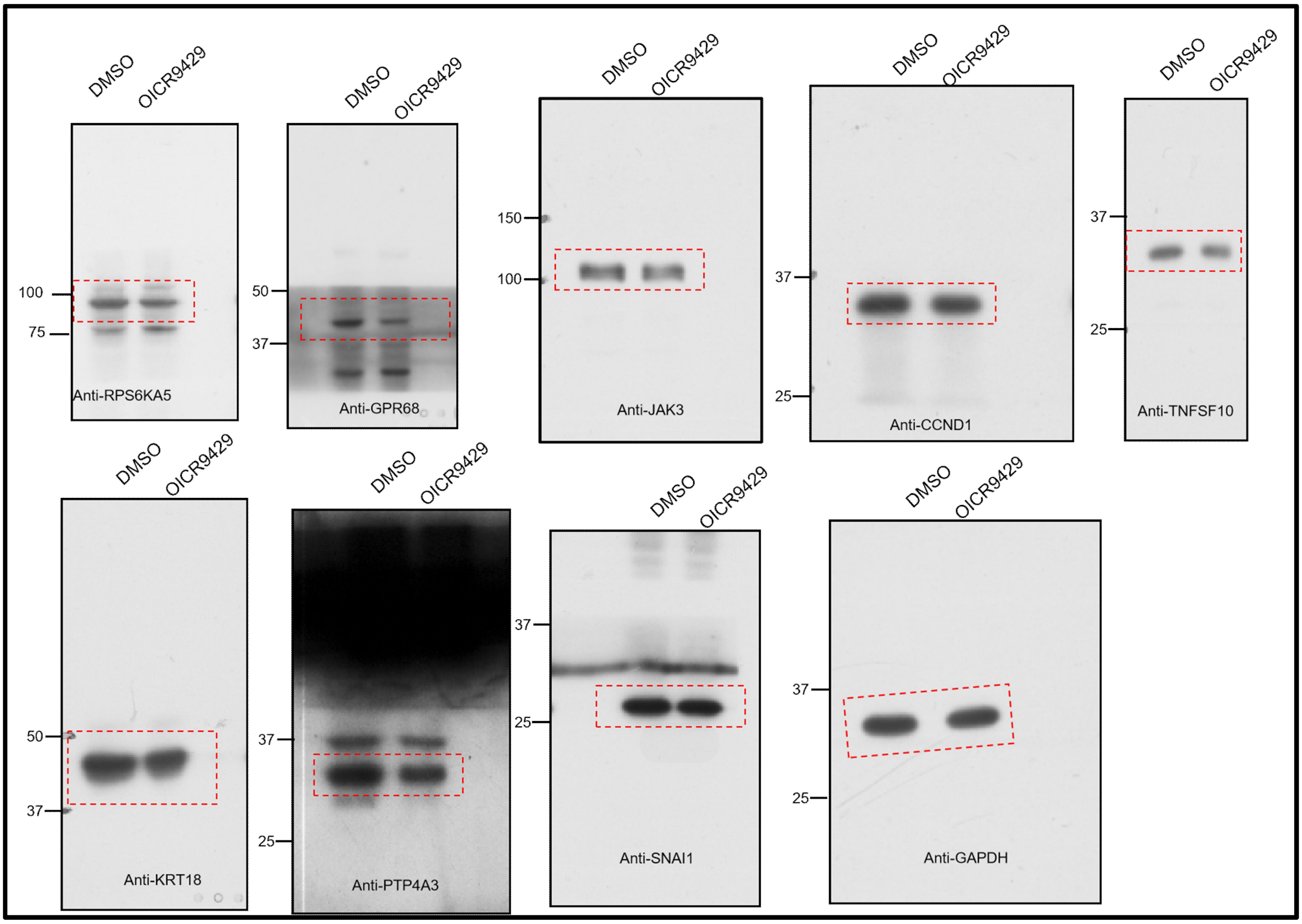

Uncropped western blotting and gel for Fig. S9

Fig. S9B

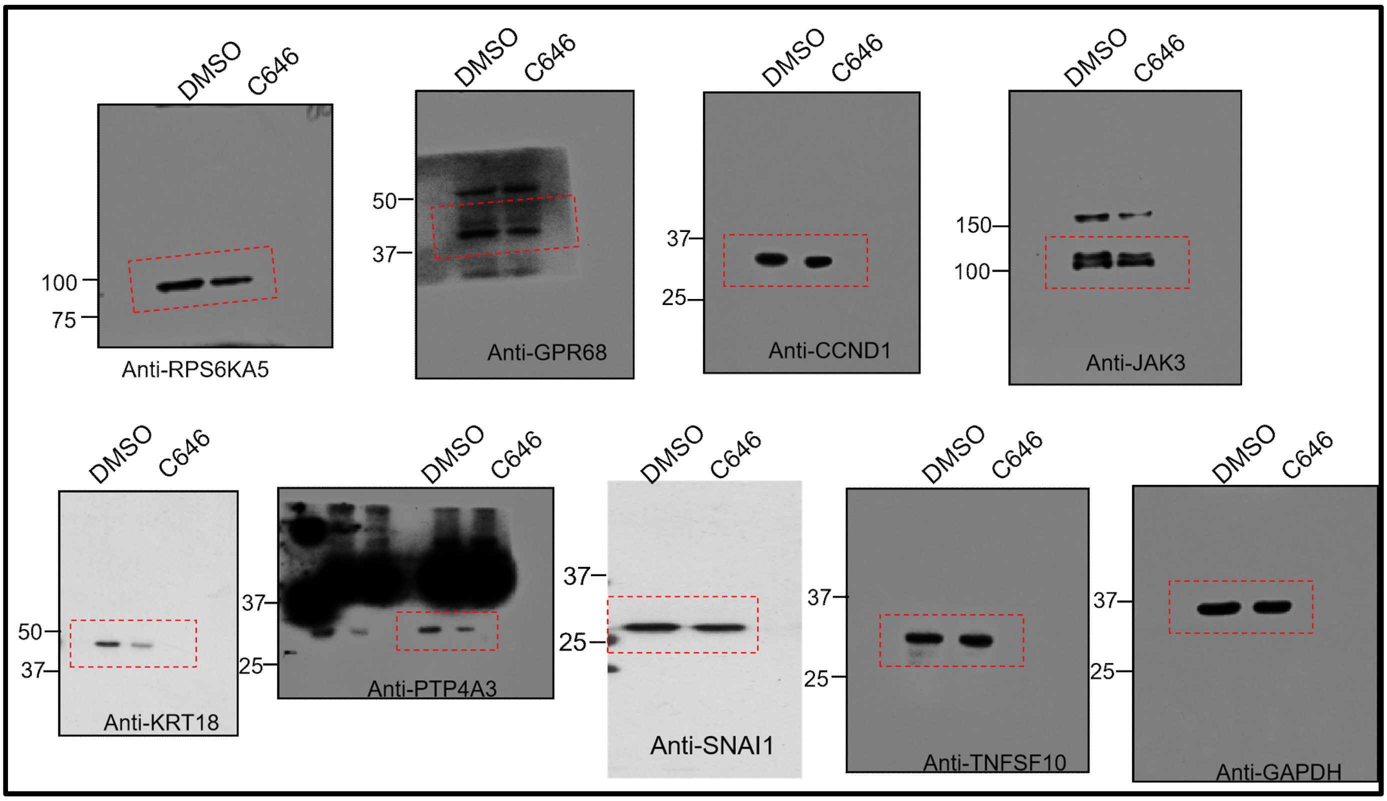

Uncropped western blotting and gel for Fig. S10

Fig. S10D

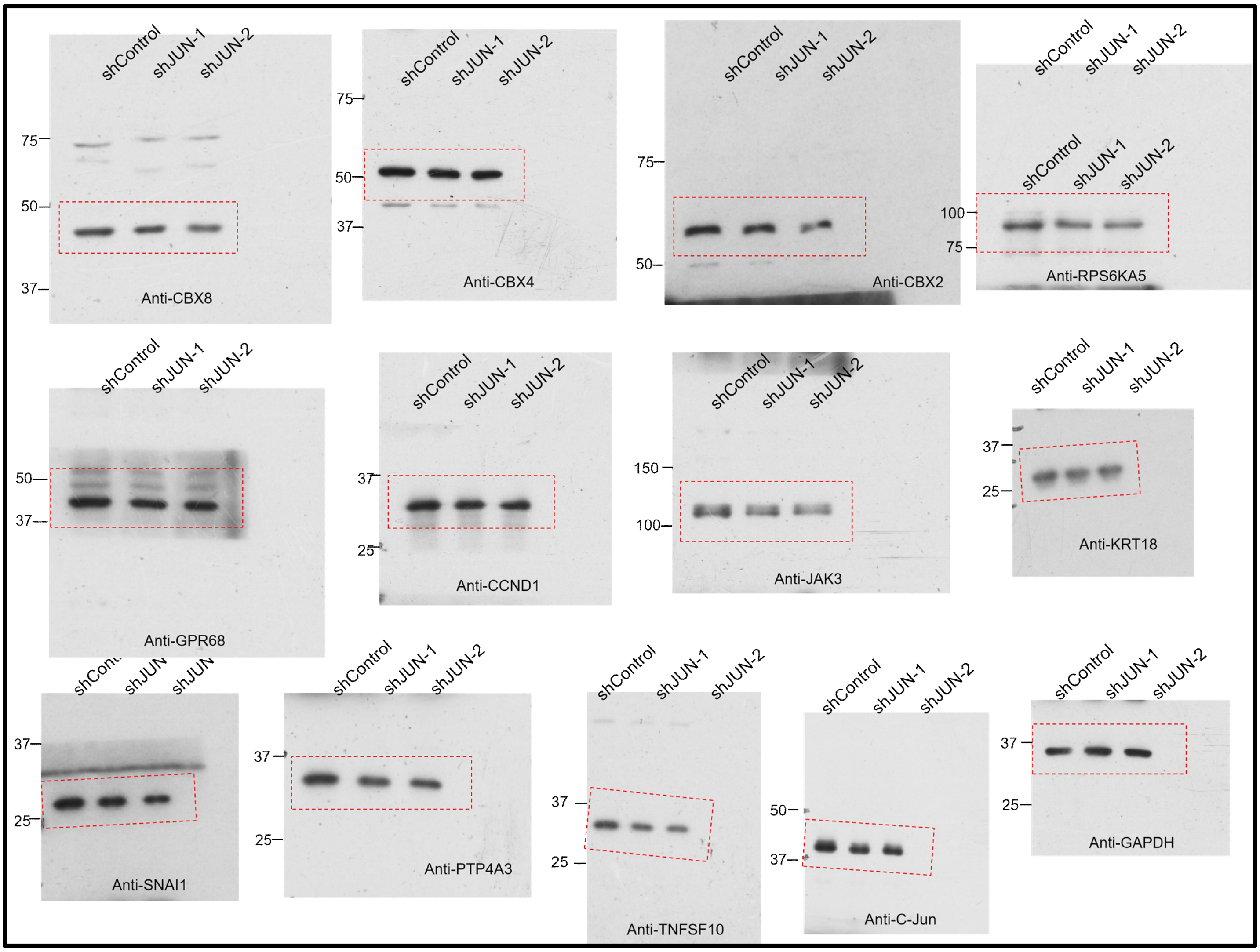

Supplement: Supplementary file 4 — Additional file 4. Uncropped images for the blots in Figure 5-6, Fig S5 and Fig S7-10. [file 13059_2023_3108_MOESM4_ESM.pdf]
