# Supplementary material for: Cooperation of MLL1 and Jun in controlling H3K4me3 on enhancers in colorectal cancer
Source: Genome Biol. 2023 Nov 27;24:268. doi: 10.1186/s13059-023-03108-3 (PMC10680327; doi:10.1186/s13059-023-03108-3)
Supplement: Supplementary file 5 — Additional file 5. Review history. [file 13059_2023_3108_MOESM5_ESM.docx]

Review History

**First round of review**

**Reviewer 1**

**Were you able to assess all statistics in the manuscript, including the appropriateness of statistical tests used?**

Yes, and I have assessed the statistics in my report.

**Comments to author:**

The authors investigated H3K4me3-enriched enhancers (m3Es) in colorectal cancer. They identified gained and lost m3Es that are enriched in immune response pathways and demonstrated that CBX8 and RPS6KA5 m3Es inhibit target gene expression and colorectal cancer. Mechanistically, they showed that MLL1 and AP1/JUN regulate m3Es activity, and the inhibitor of MLL1 also inhibits colorectal cancer in a mouse model. This work is of interest to researchers in the fields of epigenetics, transcription, and cancer and provides a unique resource for a specific type of enhancer (m3Es). I would support the publication of this work if some minor issues can be addressed, as listed below:

1. The authors defined H3K27ac/H3K4me3 peaks 2kb away from the TSS for m3Es identification. It would be helpful to clarify why 2kb was chosen instead of 3kb or 5kb and provide information on the length distributions of H3K27ac or H3K4me3 peaks. Additionally, it would be useful to explain how the authors separated the H3K27ac peaks at the TSS from the peaks at enhancers when there is overlap or if they are very large.

2. It would be interesting to see more information about the features of m3Es, such as overlap with alternative TSS, unannotated ncRNAs, or pseudogenes. What are the relationships with super-enhancers, and which types of RNA polymerases occupy them? Do they have higher levels of RNA transcription compared to other types of enhancers?

3. The authors identified different numbers of m3Es in different tumor tissues, and these enhancers are relatively small. It is unclear how the authors know that these enhancers are not variations of ChIP-seq data. For example, ChIP-seq data for normal and tumor tissues could be quite heterogeneous. Did the authors use spike-in or internal normalization for their ChIP-seq data?

4. It would be informative to conduct typical or super-enhancer gene ontology analysis and determine whether CBX8 and RPS6KA5 also regulate these enhancers. I am surprised that many m3Es have effects on colorectal tumors, as enhancers usually fine-tune gene expression in a spatial and temporal-dependent manner. Can the authors discuss why m3Es have strong physiological effects?

Minor:

The quality of Western blot data for MLL1 in Figure 6F is low.

Why did the authors perform JUN overexpression ChIP? Was it due to a lack of high-quality endogenous antibodies?

**Reviewer 2**

**Were you able to assess all statistics in the manuscript, including the appropriateness of statistical tests used?**

Yes, and I have assessed the statistics in my report.

**Comments to author:**

Lin et al. report the results of genomic and functional studies showing the lansdcape of H3K4me3 on enhancers in 43 pairs of colorectal cancer (CRC) samples, and that MLL1 and JUN regulate H3K4me3 activity on enhancers. The study also suggests a potential novel strategy for cancer treatment based on MLL1 inhibitor.
The study introduce new insights, that were not previously explored in the context of H3K4me3 activity in colorectal cancer that may be relevant for novel therapeutic strategies. However, a limitation of this study is that it does not identify the specific cell types within the tumor heterogeneity. Therefore, it is not possible to determine the percentage of non-tumor cells in the tumor sample and whether the increase in H3K4me3 binding sites is specific to a particular cell type within the tumor.

The specific comments as followed:
1. The authors defined active enhancers bases on H3K27ac peaks 2kb away form transcription start site (TSS). Recent publications have defined H3K27ac and H3K4me3 marks as active promoters, while H3K27ac and H3K4me1 marks are defined as active enhancers. Considering the proximity of the identified sites to the TSS in this study, wouldn't the sites identified be active promoters rather than enhancers? (Sharifi-Zarchi, A., et al. BMC Genomics 18, 964 (2017); Heintzman, N., et al. Nat Genet 39, 311-318 (2007))
2. Estudies examining the entire genome have indicated that AP-1 binds to promoter regions or regions close to promoters, but the recent findings suggest that its main effects on gene transcription occur when it binds to regulatory elements located at a distance from the gene's TSS, sometimes very far away (Bejjani F, et al. Biochim Biophys Acta Rev Cancer. 2019 1872(1):11-23). What would be the percentage of AP-1 binding to distal enhancers in this study? Would they also be enriched in immune response? Please comment on and discuss this aspect.
3. Have sites marked with H3K27ac and H3K4me3 been identified in this study that are distal to the TSS (greater than 2 kB from the TSS)?
4. The results do not show any western blot demonstrating altered abundance of the proteins encoded by the genes under study (e.g., Fig 5, Fig 6, S5, S7, S8, S9, S10). It would be important to demonstrate that the levels of the proteins encoding the target genes studied are also decreased.
5. Indicate the direction of transcription and distance to the TSS in supplementary Figure S3.
6. Include the protocol for native-chip in the methods section.
7. In the discussion, it is mentioned that JNK/JUN inhibitors were not effective due to the complex functions of JNK/JUN in multiple pathways. Considering the importance the authors suggest regarding the regulation of H3K4me3 activity by JUN, it would be relevant to investigate why this treatment has not been effective, such as whether the inhibitor has not affected the transcriptional activity of Jun, for example.

**Authors Response**

**Point-by-point responses to the reviewers’ comments:**

Reviewer #1: The authors investigated H3K4me3-enriched enhancers (m3Es) in colorectal cancer. They identified gained and lost m3Es that are enriched in immune response pathways and demonstrated that CBX8 and RPS6KA5 m3Es inhibit target gene expression and colorectal cancer. Mechanistically, they showed that MLL1 and AP1/JUN regulate m3Es activity, and the inhibitor of MLL1 also inhibits colorectal cancer in a mouse model. This work is of interest to researchers in the fields of epigenetics, transcription, and cancer and provides a unique resource for a specific type of enhancer (m3Es). I would support the publication of this work if some minor issues can be addressed, as listed below:

*Response: We appreciate for the positive comments.*

1. The authors defined H3K27ac/H3K4me3 peaks 2kb away from the TSS for m3Es identification. It would be helpful to clarify why 2kb was chosen instead of 3kb or 5kb and provide information on the length distributions of H3K27ac or H3K4me3 peaks. Additionally, it would be useful to explain how the authors separated the H3K27ac peaks at the TSS from the peaks at enhancers when there is overlap or if they are very large.

*Response: The procedures for “****Identification of H3K4me3-enriched enhancers****” have been described in the method section (line 505-514). We have added more description about m3E identification and data analysis in the method section (line 482-484). In general, all three parameters have been frequently used in different publications. In order not to miss important enhancers, we generally use peaks 2kb away from TSS for analysis in our previous and current studies [1, 2], which is also frequently used by other groups [3-5].*

*To identify the significant v3mEs between native and tumor tissue, we first identified active enhancers based on H3K27 peaks 2 kb away from transcription start site (TSS), and then overlapped them with H3K4me3 peaks 2 kb away from TSS. The above description was shown in the section of “Materials and methods - Identification of H3K4me3-enriched enhancers”. To further clarify it, we calculated the identified the percentages of the total m3Es and tumor-specific m3Es at difference to TSS (Response Fig. 1).* *The result indicates that no enhancers are located within 2 kb to TSS, around 10% m3Es between 2-3 kb, and more than* *60% are* *more than 10 kb away from TSS.*

**Response Fig. 1** The percentage of identified m3Es at difference distance to TSS. The purple lines represent all the identified m3Es, and the blue lines represent the significant tumour specific m3Es.

2. It would be interesting to see more information about the features of m3Es, such as overlap with alternative TSS, unannotated ncRNAs, or pseudogenes. What are the relationships with super-enhancers, and which types of RNA polymerases occupy them? Do they have higher levels of RNA transcription compared to other types of enhancers?

*Response: We have analyzed the relationship between m3Es with the mentioned elements. The results indicate that most of the identified m3Es do not overlap with alternative TSS, ncRNAs or pseudogenes; however, a portion of m3Es do overlap with them (Response Fig. 2&3). Especially for those overlapped with alternative TSS, it is possible some of them might function as alternative promoters for transcription variants. Unfortunately, based on the current data we have, it is really difficult to determine whether they function as enhancers or alternative promoters. It requires a large amounts of experiments for verification. Based on* *our experiments, at least most of the tested m3Es function as active enhancers and some of them regulates the transcription of multiple proximal genes (Fig.3 & Sup. Fig. S5). Therefore, we think most of the identified m3Es should have enhancer functions.*

**Response Fig. 2** Bar plot for the proportion of total m3Es (A) and significant Vm3Es (B) overlap with alternative TSS, ncRNAs, and pseudogenes.

**Response Fig. 4 Relationship between m3Es and super enhancers.** (A) Venn diagram to show the number of the identified m3Es overlapping with total super enhancers, and (B) variant m3Es overlapping with variant super enhancers. (C & D) Box plot to show the normalized eRNA expression of the indicated enhancers in. (E) Fold change eRNA expression associated with indicated enhancers

**Response Fig. 3** Overlapping of the H3K4me3 promoters with the identified total m3Es (left), or the significant Vm3Es (right).

*For m3Es and SEs, they are two groups of enhancers with different features. SEs are the combination of active enhancers close to each other which facilitates TF regulation; m3Es are defined by histone modifications and localizations. M3Es are usually much narrow than SEs, but a portion of them do overlap (Response Fig. 4A&B). They can both stimulate higher gene transcription compared with typical enhancers (Response Fig. 4C-E). Based on our observations, SEs maybe have higher activity towards transcription activation, but often not significant.*

*To determine the relationship between m3Es and RNA polymerases, we first used siRNAs to knockdown RNA polymerase I, II and III, and then examined the effects on the target genes of the identified m3Es. The results show that only RNA pol II knockdown caused significant decrease of the eRNAs transcribed from m3Es (Response Fig. 5). Then we utilized ChIP assay to study the binding of RNA Pol II on the m3Es, which indicate that RNA Pol II does bind to the tested m3Es (Response Fig. 6). Finally, we treated the cells with DRB and examined eRNAs transcribed from m3Es and found that RNA Pol II inhibition repressed the levels of the eRNAs (Response Fig. 7). All these together indicate that RNA Pol II binds to* *m3Es and* *regulates their functions.*

**Response Fig. 5** HCT116 and RKO cells were transfected with siRNAs of POLR2A (A), PLOR1A (B), POLR3A (C) for 48 h, and relative expression level of eRNAs transcribed by m3E enhancers were determined by qRT-PCR.

**Response Fig. 7** Bar plot showing the relative expression level of eRNAs transcribed by m3Es in HCT116 and RKO after DRB (50 μM) treatment for 2h.

**Response Fig. 6** ChIP-qPCR showing the POLR2A enrichment level on m3Es. GAPDH promoter are used as the positive and one site within centromere as negative control in HCT116 (up) and RKO (bottom).

3. The authors identified different numbers of m3Es in different tumor tissues, and these enhancers are relatively small. It is unclear how the authors know that these enhancers are not variations of ChIP-seq data. For example, ChIP-seq data for normal and tumor tissues could be quite heterogeneous. Did the authors use spike-in or internal normalization for their ChIP-seq data?

*Response: We used input from each sample for normalization, but did not add spike-in during ChIP-Seq analysis. The detailed procedures were described in the section of Materials and methods. We noticed that different numbers of variant m3Es exist in different pairs of tissues. The difference could be due to many causes, such as individual difference, different gene mutations, or discrepancy during sample collection. The exact reason should be determined in the future studies with more samples.*

4. It would be informative to conduct typical or super-enhancer gene ontology analysis and determine whether CBX8 and RPS6KA5 also regulate these enhancers. I am surprised that many m3Es have effects on colorectal tumors, as enhancers usually fine-tune gene expression in a spatial and temporal-dependent manner. Can the authors discuss why m3Es have strong physiological effects?

*Response: We have published the gene ontology analysis about gain VELs (typical enhancers) identified in the current study in our previous publication [2], which was shown here in Response Fig. 8. The result of biological processes enriched in gain VSELs (super-enhancers) is shown in Response Fig. 9.*

**Response Fig. 8** Gene function analysis for the proximal genes of the gain and lost variant enhancers in CRC.

**Response Fig. 9** Gene function analysis for the proximal genes of the gain and lost variant super enhancers in CRC.

*We do not get why it is necessary to show whether CBX8 and RPS6KA5 regulate these enhancers,* *since CBX8 and RPS6KA5 are just target genes of the identified m3Es. Especially, RPS6KA5 is considered as a cytoplasmic protein involved in metabolism and cancer, but not in chromatin regulation. CBX8 is a protein containing chromo domain, which may bind methylated lysine in histones; but its exact biochemical function needs to be determined. Here we just showed the H3K27ac and H3K4me3 tracks for the two genes (Response Fig. 10).* *We will be pleased to present more information if required.*

*The reprogramming of active enhancers during tumorigenesis and metastasis has been recently shown in several publications by different groups [2, 4-7]. I agree that enhancers usually fine-tune gene expression in a spatial and temporal-dependent manner, while certain enhancers may play critical roles in development and diseases.* *During tumorigenesis, activation of signaling pathways causes subsequent activation of transcription factors in cells, which then binds to their specific enhancers and regulate downstream gene expression. Sometimes, one enhancer regulates the expression of multiple genes, which multiplies the effect and promotes tumorigenesis. Though numerous enhancers exit, they are not equal in certain processes. Among them, m3Es and SEs have relative higher activity to promote transcription, and are highly possible to have important functions during tumorigenesis and metastasis. The tumor-specific active m3Es and SEs may become new targets for diagnosis and drug treatment. We have added some of the above in to discussion (line 308-312).*

Minor:

The quality of Western blot data for MLL1 in Figure 6F is low.

*Response: We have repeated the experiment, and replaced the figure (Response Fig. 11).*

**Response Fig. 11** Immunoprecipitation of endogenous MLL1 and JUN.

Why did the authors perform JUN overexpression ChIP? Was it due to a lack of high-quality endogenous antibodies?

**Response Fig. 10** The genome browser view to show the ChIP-seq density of H3K27ac on enhancers in their proximal genes, *CBX8* (up) and *RPS6KA5* (bottom).

*Response: Yes, we had some problems with the JUN antibody in ChIP assay. During revision, we have successfully found a workable antibody and performed ChIP assay with JUN* *endogenous protein. The data indicated the same conclusion as before (Response Fig. 12).*

**Response Fig. 12** ChIP-qPCR showing JUN enrichment on the tested m3Es in HCT116 (up) and RKO (bottom), with an antibody recognizing phosphorylated JUN. A centromere site was used as the negative control.

Reviewer #2: Lin et al. report the results of genomic and functional studies showing the lansdcape of H3K4me3 on enhancers in 43 pairs of colorectal cancer (CRC) samples, and that MLL1 and JUN regulate H3K4me3 activity on enhancers. The study also suggests a potential novel strategy for cancer treatment based on MLL1 inhibitor.

The study introduce new insights, that were not previously explored in the context of H3K4me3 activity in colorectal cancer that may be relevant for novel therapeutic strategies. However, a limitation of this study is that it does not identify the specific cell types within the tumor heterogeneity. Therefore, it is not possible to determine the percentage of non-tumor cells in the tumor sample and whether the increase in H3K4me3 binding sites is specific to a particular cell type within the tumor.

*Response: We appreciate for the reviewer’s positive comments. About the mentioned limitation, we also agree that it is important to identify cell-type specific active enhancers in tumor and other tissues. It could be partially solved using single cell ATAC-Seq, but it remains debate whether the results of ATAC-Seq can nicely reflect the global profiles of active enhancers. The ideal experiment to solve the problem is single cell ChIP-Seq analysis. However, it is still a big challenge in the field to do it with tissues. We are collaborating with a company to further develop the assay with CRC tumors, but it will take a quite long time to get the result. To address the reviewer’s concern, since we have already confirmed the enhancer activity in cultured CRC cell lines, we deduce that the identified m3Es might be active in CRC tumor cells. So, we took the cultured organoids derived from CRC patient* *tumor cells and found positive H3K27ac and H3K4me3 peaks on the identified m3Es (Response Fig. 13), indicating these m3Es are active in CRC organoids. Since CRC organoids are considered from CRC cancer stem cells, these m3Es are probably active in CRC tumor cells. While, we still did not find a way to culture organoids from patient normal tissues, so our experiments were lack of a negative control here.*

**Response Fig. 13** Cut&Tag qPCR showing the H3K27ac and H3K4me3 level at m3Es in tumoroids derived from clinical colorectal cancer tissues. *GAPDH* promoter is used as positive control and a centromere site as negative control.

*Then we found one recent report about chromatin state analysis using CRC organoids [8]. We analyzed their data with our ChIP-Seq data in normal adjacent tissues. The results indicate that the H3K4me3 and H3K27ac level on the identified m3Es are highly correlated among CRC organoids, but not with those in the adjacent tissues (Response Fig. 14), supporting that the identified m3Es can be used to distinguish CRC organoids from normal tissues, and the modification levels on them are higher in CRC organoids than normal tissues.*

**Response Fig. 14** Correlation of H3K4me3 (A) or H3K27ac (B) signal on the regions of m3Es in tumoroids derived from clinical CRC tissues and native tissue.

*Then we used the mouse CRC model for further analysis. We established mouse AOM/DSS CRC model [1] and successfully cultured organoids from mouse colon normal and tumor* *tissues. Unfortunately, the organoids from normal tissues were growing very slowly and we did not get enough samples for analysis within the limited revision period. At the same time, we tried to isolate intestinal crypts from normal mice, and we successfully isolated normal crypts according to previous publications [9, 10]. Then, we performed Cut-Tag followed by real time PCR with normal crypts and tumor organoids. The results indicate that H3K4me3 and H3K27ac on the m3Es for Cbx8, Ccnd1, Krt18, Snai1, Tdgf1, Cep72, Hils1, and Gga have higher activity in tumor organoids than that in crypts (Response Fig. 15); H3K4me3 on the m3E for Tngsf10 is higher in tumor organoids, but not H3K27ac; and both modifications have no significant difference on Gapdh promoter. These indicate that the tested m3Es are specifically activated in CRC tumor cells.*

*Taken together, the above analyses indicate that the identified m3Es are probably specifically activated in CRC tumor cells.*

**Response Fig. 15** CRC was induced with AOM/DSS in mice and organoids were cultured from tumor tissues. Crypts were isolated from normal mouse intestine. Cut&Tag qPCR was performed to show H3K27ac and H3K4me3 on the tested m3Es. *Gapdh* promoter as positive control and a centromere site as negative control.

The specific comments as followed:

1. The authors defined active enhancers bases on H3K27ac peaks 2kb away form transcription start site (TSS). Recent publications have defined H3K27ac and H3K4me3 marks as active promoters, while H3K27ac and H3K4me1 marks are defined as active enhancers. Considering the proximity of the identified sites to the TSS in this study, wouldn't the sites identified be active promoters rather than enhancers? (Sharifi-Zarchi, A., et al. BMC Genomics 18, 964 (2017); Heintzman, N., et al. Nat Genet 39, 311-318 (2007))

*Response: I agree that H3K27ac and H3K4me1 marks are used to define as active enhancers. In the recent practices, it is found that the active enhancers predicted with H3K27ac alone are very similar to that with two modifications, so many studies are using H3K27ac for enhancer prediction [2, 11]. H3K27ac and H3K4me3 are used as marks as active promoters, when they are located to TSS; but actually scientists have observed that H3K27ac and H3K4me3 are located in the intergenic regions, and several studies have shown that these loci function as* *enhancers and have higher activity towards transcription compared with the typical enhancers [6, 12]. We have performed one analysis to calculated the percentage of the identified m3Es at the different distance to TSS, which shows that most of the identified m3Es are much farther away than 2 kb to TSS, and 60% m3Es are more than 10 kb away (Response Fig. 1). So we are confident that most of the identified m3Es are active enhancers in CRC cells.*

2. Estudies examining the entire genome have indicated that AP-1 binds to promoter regions or regions close to promoters, but the recent findings suggest that its main effects on gene transcription occur when it binds to regulatory elements located at a distance from the gene's TSS, sometimes very far away (Bejjani F, et al. Biochim Biophys Acta Rev Cancer. 2019 1872(1):11-23). What would be the percentage of AP-1 binding to distal enhancers in this study? Would they also be enriched in immune response? Please comment on and discuss this aspect.

*Response: We downloaded the genome wide data of JUN-binding sites from GEO database, and overlapped the JUN-binding sites with our enhancer profiles. The results indicate that 5.2% of the identified m3Es are overlapped with cJun sites from GSM1208639, and 7.4% are overlapped with that from GSM1239467 (Response Fig. 16&17). GO analysis for the target genes of the above enhancers and m3Es showed that they are enriched in multiple immune pathways, such as NF-κB pathway, T cell and B cell differentiation, and interleukin pathways (Response Fig. 18). We have added the mentioned reference added more discussion in the manuscript (line 300-305).*

**Response Fig. 16** Venn diagram to show the numbers of the identified enhancers (H3K27acELs, A&B) and m3Es (H3K4me3ELs, C&D) overlap with c-JUN binding site (GSM1208639, GSM1239467).

**Response Fig. 17** The heatmap showing the normalized c-JUN ChIP-seq data (GSM1208639, GSM1239467) at total enhancers (H3K27acELs) and m3Es (H3K4me3ELs).

**Response Fig. 18** Gene ontology assay to show the enriched biological processes for the proximal genes to the c-JUN sites overlapped with active enhancers (A) and m3Es (B)

Have sites marked with H3K27ac and H3K4me3 been identified in this study that are distal to the TSS (greater than 2 kB from the TSS)?

*Response: As we have shown in the response to comment 1, all the enhancers are located more than 2 kb to TSS, around 10% m3Es between 2-3 kb, and more than 60% are more than 10 kb away (Response Fig. 1).*

3. The results do not show any western blot demonstrating altered abundance of the proteins encoded by the genes under study (e.g., Fig 5, Fig 6, S5, S7, S8, S9, S10). It would be important to demonstrate that the levels of the proteins encoding the target genes studied are also decreased.

*Response: We have tried our best to get the antibodies for most of the proteins and performed* *western blotting. The data are shown in Fig. 5, 6, S5, &Response Fig 19&20. Since the number of potential target genes is quite large, it is almost impossible for us to get good antibodies for all of them. I hope the reviewer could understand our difficulty.*

**Response Fig. 19** Revised western blotting images for Figure 5D, 5J, 5M, 6C, S8D, S9B & S10D.

**Response Fig. 20** Revised western blotting images for Figure S5 & S7H.

4. Indicate the direction of transcription and distance to the TSS in supplementary Figure S3.

*Response: We have revised the figure, and replaced the original one (Response Fig. 21).*

**Response Fig. 21 The revised images for Fig. S3.**

5. Include the protocol for native-chip in the methods section.

*Response: We have included the protocol as suggested (line 405-418).*

6. In the discussion, it is mentioned that JNK/JUN inhibitors were not effective due to the complex functions of JNK/JUN in multiple pathways. Considering the importance the authors suggest regarding the regulation of H3K4me3 activity by JUN, it would be relevant to investigate why this treatment has not been effective, such as whether the inhibitor has not affected the transcriptional activity of Jun, for example.

*Response: We used one inhibitor for JUN, T-5224, which inhibits the binding between JUN and DNA. In fact, the treatment to cell lines showed the similar result as that with siRNA transfection, indicating T-5224 can repress the activity of the identified m3Es (Response Fig. 22). However, when we treat the mice carrying tumors, the drug did not show significant effects (Response Fig. 23). Then we checked the literature and found that other groups used a much higher dose in the similar experiments (higher than 100 mg/kg in mice) [13, 14]. We think the dose is too high and it may have very little clinical value, then we did not pursue further studies and chose not to show the related result in the manuscript.*

**Response Fig. 22 The effects of c-Jun inhibitor T-5224 in cells. (A&B)** The mRNA level of the proximal genes of *CBX8* and *RPS6KA5* m3Es after T-5224 treatment. **(C&D)** ChIP-qPCR assessing H3K27ac and H3K4me3 level on m3Es of *CBX8* (C) and *RPS6KA5* (D) after T-5224 (20 μM) treatment for 48h. **(E)** Cell viability analyzed by CCK-8 assay in HCT116 with T-5224 (20 μM) for 48h. **(F)** Viability of colorectal cancer organoid with T-5224 treatment (80 μM) for 7 days, measured by CCK-8 assay. **(G)** Cell cycle distributions analyzed by flow cytometry of HCT116 with T-5224 treatment (20 μM) for 48h.

**Response Fig. 23 The effects of T5224 in CRC mouse models. (A)** Xenograft experiments in nude mice were performed with HCT116 with/without T-5224 (5 mg/kg, once every two days). The tumors growth curve, volume and weight are shown. **(B)** The representative image for colons with/without T-5224 (5 mg/kg). Tumors were measured, and the total tumor number and size for each animal was plotted in a line for the DMSO and T-5224 groups

***Reference***

*1.	Chen L, Luo Z, Zhao C, Li Q, Geng Y, Xiao Y, Chen MK, Li L, Chen ZX, Wu M:* ***Dynamic Chromatin States Coupling with Key Transcription Factors in Colitis-Associated Colorectal Cancer.*** *Adv Sci (Weinh) 2022****:****e2200536.*

*2.	Li QL, Lin X, Yu YL, Chen L, Hu QX, Chen M, Cao N, Zhao C, Wang CY, Huang CW, et al:* ***Genome-wide profiling in colorectal cancer identifies PHF19 and TBC1D16 as oncogenic super enhancers.*** *Nat Commun 2021,* ***12:****6407.*

*3.	Hnisz D, Abraham BJ, Lee TI, Lau A, Saint-Andre V, Sigova AA, Hoke HA, Young RA:* ***Super-enhancers in the control of cell identity and disease.*** *Cell 2013,* ***155:****934-947.*

*4.	Ye B, Fan D, Xiong W, Li M, Yuan J, Jiang Q, Zhao Y, Lin J, Liu J, Lv Y, et al:* ***Oncogenic enhancers drive esophageal squamous cell carcinogenesis and metastasis.*** *Nat Commun 2021,* ***12:****4457.*

*5.	Bal E, Kumar R, Hadigol M, Holmes AB, Hilton LK, Loh JW, Dreval K, Wong JCH, Vlasevska S, Corinaldesi C, et al:* ***Super-enhancer hypermutation alters oncogene expression in B cell lymphoma.*** *Nature 2022,* ***607:****808-815.*

*6.	Shen H, Xu W, Guo R, Rong B, Gu L, Wang Z, He C, Zheng L, Hu X, Hu Z, et al:* ***Suppression of Enhancer Overactivation by a RACK7-Histone Demethylase Complex.*** *Cell 2016,* ***165:****331-342.*

*7.	Loven J, Hoke HA, Lin CY, Lau A, Orlando DA, Vakoc CR, Bradner JE, Lee TI, Young RA:* ***Selective inhibition of tumor oncogenes by disruption of super-enhancers.*** *Cell 2013,* ***153:****320-334.*

*8.	Della Chiara G, Gervasoni F, Fakiola M, Godano C, D'Oria C, Azzolin L, Bonnal RJP, Moreni G, Drufuca L, Rossetti G, et al:* ***Epigenomic landscape of human colorectal cancer unveils an aberrant core of pan-cancer enhancers orchestrated by YAP/TAZ.*** *Nat Commun 2021,* ***12:****2340.*

*9.	Stine RR, Sakers AP, TeSlaa T, Kissig M, Stine ZE, Kwon CW, Cheng L, Lim HW, Kaestner KH, Rabinowitz JD, Seale P:* ***PRDM16 Maintains Homeostasis of the Intestinal Epithelium by Controlling Region-Specific Metabolism.*** *Cell Stem Cell 2019,* ***25:****830-845 e838.*

*10.	Xian L, Georgess D, Huso T, Cope L, Belton A, Chang YT, Kuang W, Gu Q, Zhang X, Senger S, et al:* ***HMGA1 amplifies Wnt signalling and expands the intestinal stem cell compartment and Paneth cell niche.*** *Nat Commun 2017,* ***8:****15008.*

*11.	Yuan J, Jiang YY, Mayakonda A, Huang M, Ding LW, Lin H, Yu F, Lu Y, Loh TKS, Chow M, et al:* ***Super-Enhancers Promote Transcriptional Dysregulation in Nasopharyngeal Carcinoma.*** *Cancer Res 2017,* ***77:****6614-6626.*

*12.	Hu D, Gao X, Cao K, Morgan MA, Mas G, Smith ER, Volk AG, Bartom ET, Crispino JD, Di Croce L, Shilatifard A:* ***Not All H3K4 Methylations Are Created Equal: Mll2/COMPASS Dependency in Primordial Germ Cell Specification.*** *Mol Cell 2017,* ***65:****460-475 e466.*

*13.	Liu R, Tan J, Shen X, Jiang K, Wang C, Zhu G, Xing M:* ***Therapeutic targeting of FOS in mutant TERT cancers through removing TERT suppression of apoptosis via regulating survivin and TRAIL-R2.*** *Proc Natl Acad Sci U S A 2021,* ***118****.*

*14.	Kamide D, Yamashita T, Araki K, Tomifuji M, Tanaka Y, Tanaka S, Shiozawa S, Shiotani A:* ***Selective activator protein-1 inhibitor T-5224 prevents lymph node metastasis in an oral cancer model.*** *Cancer Sci 2016,* ***107:****666-673.*

**Second round of review**

**Reviewer 1**

I appreciate the response and the revised manuscript provided by the authors. Based on the improvements made, I am now fully satisfied with the study and support the publication of this exciting work.

**Reviewer 2**

I would like to express my appreciation for the author's diligence in addressing all the concerns I have raised earlier. However, I would like to draw your attention to one particular point in figure 6. It appears that Jun silencing is relatively weak, as there is hardly any difference in Jun protein levels between control and Jun-silenced cells. Furthermore, the behaviour of genes affected by Jun silencing varies depending on the Jun silencer used. Therefore, I suggest performing quantitative PCR to measure Jun mRNA levels and confirm the success of Jun gene silencing. Apart from this, after thorough evaluation, I believe the manuscript is ready for acceptance and publication.

**Authors Response**

**Point-by-point responses to the reviewers’ comments:**

Reviewer #1: I appreciate the response and the revised manuscript provided by the authors. Based on the improvements made, I am now fully satisfied with the study and support the publication of this exciting work.

*Response: We appreciated the reviewer for his/her efforts in improving our study.*

Reviewer #2: I would like to express my appreciation for the author's diligence in addressing all the concerns I have raised earlier. However, I would like to draw your attention to one particular point in figure 6. It appears that Jun silencing is relatively weak, as there is hardly any difference in Jun protein levels between control and Jun-silenced cells. Furthermore, the behaviour of genes affected by Jun silencing varies depending on the Jun silencer used. Therefore, I suggest performing quantitative PCR to measure Jun mRNA levels and confirm the success of Jun gene silencing. Apart from this, after thorough evaluation, I believe the manuscript is ready for acceptance and publication.

*Response: We appreciate the reviewer for the consideration of our work. About the result in Fig. 6, we have repeated the experiment and replaced the images with better results (Response Fig. 1). Thank you for pointing out our defect.*

**Third round of review**

**Reviewer 2**

The authors have satisfactorily answered the question raised in the previous version of the manuscript. Therefore, I have no further comments.
